# Cancer-Associated Fibroblast-Derived GDF15 Induces Oxidative Stress and Neutrophil Infiltration in Head and Neck Squamous Cell Carcinoma through the PI3K/AKT/STAT3 Axis Cascade

**DOI:** 10.34133/research.0901

**Published:** 2025-09-30

**Authors:** Zhijie Zhao, Huabao Cai, Zhenzhen Zhao, Xiaojing Wang, Wenyang Nie, Fu Zhao, Yisheng Chen, Yanyu Ding, Zhiwen Luo, Zhiheng Lin, Yantao Ding

**Affiliations:** ^1^ Department of Plastic and Reconstructive Surgery, Shanghai Ninth People’s Hospital, Shanghai Jiao Tong University School of Medicine, Shanghai 200011, China.; ^2^ Department of Neurosurgery, The First Affiliated Hospital of Anhui Medical University, Hefei 230032, China.; ^3^ Center for Scientific Research of Anhui Medical University, Anhui Medical University, Hefei 230032, China.; ^4^ Institute of Health and Medicine, Hefei Comprehensive National Science Center, Hefei, Anhui 230601, China.; ^5^ First Clinical Medical College, Shandong University of Traditional Chinese Medicine, Jinan 250014, China.; ^6^ Department of Rheumatology and Immunology, Tongren Hospital, School of Medicine, Shanghai Jiao Tong University, Shanghai 200336, China.; ^7^ School of Traditional Chinese Medicine, Jinan University, Guangzhou 510632, China.; ^8^ Fujian Key Laboratory of Toxicant and Drug Toxicology, Medical College, Ningde Normal University, Ningde 352100, China.; ^9^ Department of Immunology, School of Basic Medicine, Anhui Medical University, Hefei 230032, China.; ^10^ Department of Sport Medicine, Huashan Hospital, Fudan University, Shanghai 200040, China.; ^11^ Department of Gynecology, Longhua Hospital, Shanghai University of Traditional Chinese Medicine, Shanghai 200032, China.; ^12^ Department of Dermatology, The First Affiliated Hospital, Anhui Medical University, Hefei 230032, China.; ^13^ Key Laboratory of Dermatology (Anhui Medical University), Ministry of Education, Hefei 230032, China.

## Abstract

**Background:** Head and neck squamous cell carcinoma (HNSCC) is a malignant tumor of the oral mucosal epithelium. The high incidence of recurrence and metastasis presents substantial challenges for treatment, underscoring the complex molecular landscape underlying the disease. The purpose of this work is to clarify how HNSCC tumor cells and cancer-associated fibroblasts (CAFs) interact. **Methods:** Spatial transcriptome sequencing and single-cell RNA sequencing had been employed to describe the biological characteristics of CAFs in HNSCC. The biological connection between CAFs and tumor cells was verified by molecular interaction experiments. In addition, the regulatory effect of CAFs on oxidative stress in tumor cells and the phenotypic conversion of neutrophils were explored through a coculture system, a knockdown/overexpression method, flow cytometry, and animal experiments. Finally, potential small-molecule inhibitors were screened by molecular dynamics simulation and validated through in vitro and in vivo assays. **Results:** Growth differentiation factor 15 (GDF15) promoted tumor cell growth and invasion by enhancing PCNA clamp associated factor (PCLAF) transcription through interferon regulatory factor 5 modulation. Its interaction with the receptor GDNF family receptor alpha like (GFRAL) triggered chronic inflammatory signaling via the phosphatidylinositol-3 kinase/protein kinase b/signal transducer and activator of transcription 3 pathway, which led to oxidative stress imbalance and contributed to tumor progression and the development of drug resistance. Moreover, GDF15 activated the extracellular signal-regulated kinase 1/2 pathway through tumor necrosis factor-α, thereby facilitating neutrophil infiltration and promoting lung metastasis in HNSCC. Notably, risperidone (SM-2) emerged as a potential inhibitory regulator capable of disrupting the cascade effects mediated by the GDF15–GFRAL axis, underscoring its therapeutic relevance. **Conclusion:** This study identifies the GDF15–GFRAL signaling axis as a critical regulator of oxidative stress and immune evasion in HNSCC and demonstrates that the novel small-molecule SM-2 effectively targets this pathway, highlighting its potential as a promising therapeutic strategy.

## Introduction

With a 5-year survival rate of less than 50%, head and neck squamous cell carcinoma (HNSCC) ranks as the sixth most common cancer globally and is a serious threat to human health. It is a malignant tumor that develops in the mucosal epithelium of the oral cavity, pharynx, and larynx [1]. The traditional approach to treating HNSCC involves a mix of chemotherapy, radiation, and surgery. In recent years, the combination of programmed cell death protein 1/programmed cell death-ligand 1 inhibitors with other immune checkpoint blockade therapies and conventional cancer treatments has demonstrated enhanced therapeutic efficacy, reduced treatment-related morbidity, and improved safety profiles [2,3]. However, this multimodal treatment strategy is largely ineffective against HNSCC, a type of recurrent and metastatic tumor [4]; the overall remission rates of patients remain low [5,6].

Fibroblasts within the tumor microenvironment (TME) exhibit marked phenotypic and functional heterogeneity. Several studies have demonstrated that cancer-associated fibroblasts (CAFs) contribute to the growth of tumors via several pathways during various phases of the tumor’s life cycle [7–9]. CAFs have long been studied as a potential therapeutic target for HNSCC and are a crucial component of the TME. However, the specific molecular mechanisms by which CAFs reshape the HNSCC TME remain largely unexplained, and the lack of specific biomarkers for CAFs has hindered their clinical application. Our goal in this work was to investigate the interactions between tumor cells and CAFs in the HNSCC TME. In order to provide a theoretical framework and cell-based insights for the development of molecular targeted therapeutics for HNSCC, our objective was to clarify the possible molecular pathways driving their interaction. Our research is crucial for directing HNSCC treatment and pharmacological development.

## Results

### Identification of a subtype of fibroblasts with active proliferation and enhanced stemness in HNSCC

**Fig. 1.**
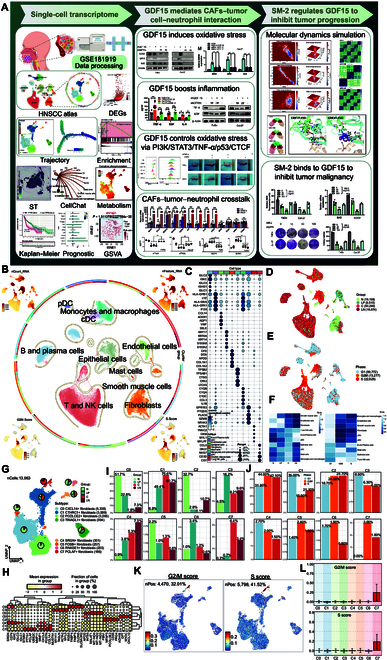
Visualization of fibroblast subtypes in head and neck squamous cell carcinoma (HNSCC). (A) Overall flowchart of this study. (B) The circular plot visualizes the clustering distribution of cells within HNSCC, with cells colored based on their respective categories. Double circular orbits (from outer to inner) sequentially represent cell types and sample tissue sources and were stained according to the cell categories. Starting from the upper left corner and proceeding clockwise, the 4 illustrations display the nCount-RNA and nFeature-RNA for all cells, along with the scores for the S phase and G2/M phase of the cells. (C) For various cell types, the bubble plot shows the differential expression of the top 5 most important marker genes; the size of the bubbles denotes the percentage of expression, while the color of the bubbles indicates the intensity of expression. (D and E) Various cell types’ distributions across sample tissue sources and cell cycle phases are shown using UMAP plots. (F) The heatmaps show that we calculated the Ro/e values for each cell type in different sample tissue sources and at different cell cycle phases, in order to quantify the distribution deviations of these cells. (G) A total of 13,963 high-quality fibroblasts from HNSCC were clustered into 8 distinct clusters. (H) Bubble plot illustrates how the top 5 marker genes expressed differently in various fibroblast subtypes. (I and J) Bar graph visualization illustrates the proportion of fibroblast subtypes in various sample tissue sources and cell cycle phase. (K and L) UMAP plots and bar graphs display the cell phase G2/M and cell phase S scores of fibroblast subtypes. DEG, differentially expressed genes; GSVA, gene set variation analysis; ST, spatial transcriptome.

We assembled single-cell sequencing datasets from 32 patients, encompassing 4 distinct stages: normal tissue (N), precancerous leukoplakia (LP), primary carcinoma (C), and lymph node metastases (LN). Figure 1A illustrates the general workflow of this study. Following rigorous quality control and unsupervised clustering, 95,677 high-quality cells were retained to illustrate the evolution of single-cell landscapes during HNSCC progression. These cells were classified into specific cell types according to distinct gene expression markers (Fig. 1B), including B cells and plasma cells, conventional dendritic cells (cDC), endothelial cells, epithelial cells, fibroblasts, mast cells, monocytes and macrophages, plasmacytoid dendritic cells, smooth muscle cells, T cells, and natural killer cells. uniform manifold approximation and projection (UMAP) plots revealed that epithelial cells exhibited elevated nCount-RNA and nFeature-RNA expressions, indicating active cell proliferation. The bubble plot illustrated the expression of marker genes across various cell types in HNSCC. As depicted in Fig. 1C, fibroblasts prominently expressed classic markers, including complement factor D, decorin, apolipoprotein d, collagen type I alpha 1 chain, and collagen type I alpha 2 chain. Figure 1D and E display the organization type and cell cycle phase distributions of all cells. Fibroblasts were distributed in all 4 tissue types, and the majority of fibroblasts reside in the G1 phase, indicative of a pre-proliferative state. The Ro/e (ratio of observed to expected cell numbers) distribution preference diagrams were consistent with these findings (Fig. 1F).

As one of the most crucial stromal components, fibroblasts play an indispensable role in cancer progression owing to their functional diversity. Based on cluster-specific gene expression profiles, we identified 8 distinct fibroblast subtypes (Fig. 1G and H). These subtypes were defined by elevated expression of characteristic marker genes and included C0 (CXCL14^+^), C1 (CTHRC1^+^), C2 (PCOLCE2^+^), C3 (TINAGL1^+^), C4 (SRGN^+^), C5 (FOSB^+^), C6 (RNASE1^+^), and C7 (PCLAF^+^) fibroblasts.

**Fig. 2.**
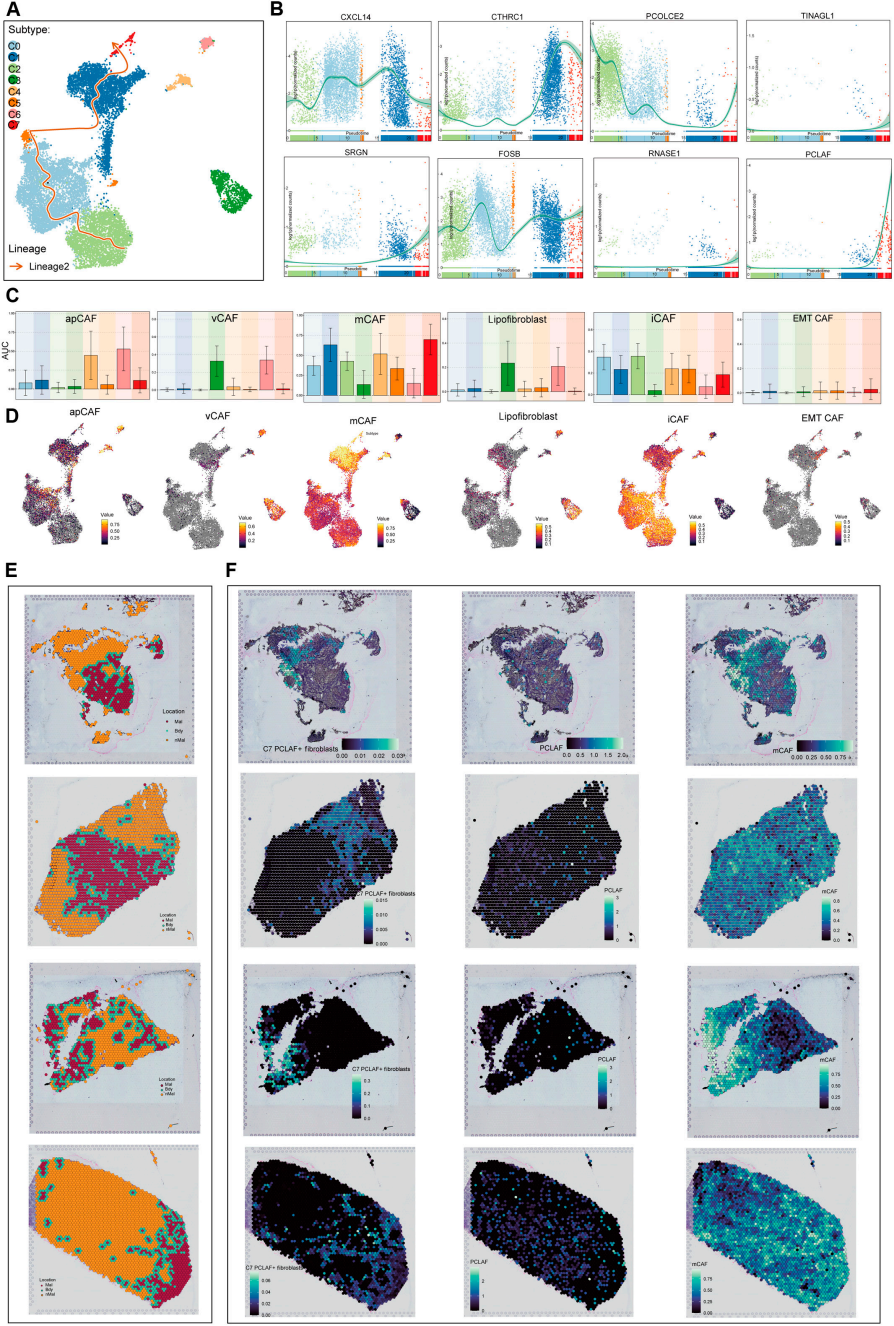
The heterogeneous graph of fibroblast subtypes in HNSCC. (A) The UMAP plot illustrates the trajectory and distribution of lineage 2 cell differentiation trajectory fitted through Slingshot across all fibroblasts. The direction of differentiation (from naïve to mature) is represented by arrows. Lineage 2: C2 PCOLCE2^+^ fibroblasts→C0 CXCL14^+^ fibroblasts→C5 FOSB^+^ fibroblasts→C1 CTHRC1^+^ fibroblasts→C7 PCLAF^+^ fibroblasts. (B) Scatterplots were utilized to show the variation in the distribution of marker genes across the 8 fibroblast subtypes in lineage 2, ordered by the suggested pseudotime. (C and D) The bar graphs and UMAP plots illustrate the AUCell scores of vascular-cancer-associated fibroblast (vCAF)-, lipofibroblast-, matrix CAF (mCAF)-, inflammatory CAF (iCAF)-, antigen-presenting CAF (apCAF)-, and mesenchymal CAF (EMT-CAF)-related genes in 8 subtypes of fibroblasts. (E) Tissue slides were annotated by malignant spots (Mal, red), boundary spots (Bdy, blue), and nonmalignant spots (nMal, orange). (F) TransferData illustrates the spatial transcriptomic landscape of the C7 PCLAF^+^ fibroblasts in different samples, gene PCLAF, and mCAF-related genes in HNSCC.

The sources of the samples and cycle phase distribution of all fibroblasts are illustrated in Fig. S1A and B and Fig. 1I and J. While most cells in the TME were in the G1 phase overall, certain fibroblast subtypes were enriched in the S and G2/M phases, suggesting heightened proliferative states. C0 CXCL14^+^ fibroblasts and C2 PCOLCE2^+^ fibroblasts constituted a substantial proportion in normal tissues and may represent normal fibroblasts. In contrast, C1 CTHRC1^+^ fibroblasts and C7 PCLAF^+^ fibroblasts predominantly originated from tumor tissues (C and LN), suggesting that they may be malignant fibroblasts. C3 TINAGL1^+^ fibroblasts and C6 RNASE1^+^ fibroblasts were primarily derived from the LP group and may act as intermediate cells in the transition from normal fibroblasts to CAFs. Most fibroblasts are distributed in all 3 cycle stages. In particular, fibroblasts of the C7 subtype, characterized by elevated PCNA clamp-associated factor (PCLAF) expression, exhibited an increased proportion of cells in the S phase (DNA synthesis) and G2/M phase (mitotic preparation and execution), indicating that this population is in a highly proliferative state (Fig. 1K and L). Furthermore, we sought to verify the association between C7 PCLAF^+^ fibroblasts and cell cycle activity. As shown in Fig. S1C and Fig. 2F (first panel), robust cell type decomposition was applied to infer the spatial distribution of the C7 subtype, revealing preferential enrichment in regions with elevated G2/M and S phase scores.

Stemness features are generally associated with enhanced proliferative potential [10]. To this end, we used AUCell and CytoTRACE to assess the stemness expression of various fibroblast subtypes. As illustrated in Fig. S1D to F, C7 PCLAF^+^ fibroblasts exhibited pronounced stemness characteristics, suggesting that they possess substantial differentiation potential, a low degree of differentiation, and elevated malignancy, which confirmed that they might be CAFs as previously mentioned.

### Characterization of the biological properties of C7 PCLAF^+^ fibroblasts

To better elucidate the roles of fibroblast subtypes during HNSCC progression, we performed enrichment analysis and differentially expressed genes among the fibroblast subtype (Fig. S1G). According to the aforementioned findings, C7 PCLAF^+^ fibroblasts showed enrichment in pathways linked to mitotic sister chromatid separation, regulation of sister chromatid segregation, chromosome separation, and regulation of chromosome separation.

Normal fibroblasts may undergo transformation into CAFs through certain pathways, exhibiting increased malignant features and metabolic activity. C7 PCLAF^+^ fibroblasts exhibit markedly activated oxidative phosphorylation and pyrimidine metabolism signatures (Fig. S1H to K). Consistently, these metabolic pathways are also highly activated in tumor samples. As one of the important sources of cellular energy, oxidative phosphorylation is a crucial mechanism by which most cancer cells meet their energy demands and serves as a key factor promoting their survival and invasion [11]. In addition, tumor chemotherapy resistance has also been found to be associated with imbalances in pyrimidine metabolism and ubiquitination [12,13]. These findings further support that C7 PCLAF^+^ fibroblasts are closely related to tumors.

To further elucidate the lineage differentiation order of fibroblast subtypes during HNSCC progression, we utilized Slingshot to infer the developmental trajectory of fibroblasts. In line with previous observations, the differentiation trajectory of fibroblasts originated from the C2 and C0 subtypes, primarily derived from normal tissues. The C1 and C7 subtypes derived from tumor tissues were mainly located at the terminal stage of lineage 2 (Fig. 2A and Fig. S2A). These results indicated that lineage 2 may reflect the trajectory of fibroblast transformation from normal to malignant states. Furthermore, the expression changes of marker genes for each subtype in the differentiation lineage corresponded to the distribution of subtype in the pseudotime trajectory (Fig. 2B and Fig. S2B).

To further interpret the biological implications of this lineage transformation, we sought to investigate the functional heterogeneity among these fibroblast subtypes. Research indicates that fibroblasts in the TME can be categorized into 6 distinct subtypes based on varying gene expression and functional characteristics [14–16]. These subtypes include vascular CAF (vCAF), lipofibroblast, matrix CAF (mCAF), inflammatory CAF (iCAF), antigen-presenting CAF (apCAF), and mesenchymal CAF (EMT-CAF). To explore the functional preferences of different fibroblast subtypes of HNSCC, we characterized fibroblasts by AUCell scoring. The biological characteristics of C7 PCLAF^+^ fibroblasts exhibited a stronger inclination toward mCAF and EMT-CAF profiles. Notably, elevated mCAF scores were also observed in tumor samples (C and LN) (Fig. 2C and D and Fig. S2C). These findings suggest that C7 PCLAF^+^ fibroblasts may contribute critically to extracellular matrix remodeling within the TME, a role further supported by spatial transcriptomic analysis. The results of tumor boundary analysis demonstrated that C7 PCLAF^+^ fibroblasts were closely related to malignant tumor cells (Fig. 2E). C7 PCLAF^+^ fibroblasts that were enriched in regions were focused on the regions with high expression of mCAF signature genes (Fig. 2F).

### TGFB1 in tumor cells negatively regulated PCLAF expression in fibroblasts

Given their prominent spatial localization and mCAF-like characteristics, we investigated whether C7 PCLAF^+^ fibroblasts engage in specific intercellular communication with other cell types in the TME. On this basis, we inferred the global communication landscape between fibroblast subtype and other cell types (Fig. S2D). Further, we analyzed the interaction between tumor cells and C7 PCLAF^+^ fibroblasts and found that compared with other fibroblast subtypes, the interaction between C7 PCLAF^+^ fibroblasts and tumor cells was relatively stronger, especially when C7 PCLAF^+^ fibroblasts acted as signal senders. Meanwhile, the transforming growth factor beta (TGF-β) signaling pathway played an active role in the communication between C7 PCLAF^+^ fibroblasts and tumor cells (Fig. S2E to H). This crosstalk was mainly mediated by the TGFB1-(TGFBR1+TGFBR2) receptor–ligand pair and occurs in a paracrine manner (Fig. S2I and J). In the TGF-β signaling pathway, C7 PCLAF^+^ fibroblasts tended to act as “receivers” while tumor cells acted as “senders” (Fig. S2K).

To explore the expression patterns of TGFB1 and PCLAF in HNSCC, we first analyzed their expression levels in clinical samples. Analyses of samples demonstrated that PCLAF and TGFB1 expressions were much higher in tumor (T) than in adjacent normal (N) tissues (Figs. S3A to C and S4A to D). There are clear architectural differences between tumor and normal tissues, and PCLAF has prominent stromal expression in the TME (Fig. S3D). To determine whether PCLAF levels are increased in CAFs, we isolated CAFs and normal fibroblasts (NFs), and confirmed their identity using immunofluorescence staining for alpha-smooth muscle actin and fibroblast activation protein alpha (Fig. S3E). Interestingly, PCLAF was primarily up-regulated in CAFs, while TGFB1 up-regulation was predominantly detected in tumor cells (FaDu and Tu212) (Figs. S3F to H and S4E). Consistently, when PCLAF was overexpressed in fibroblasts, their morphology becomes more invasive (Fig. S5A). When TGFB1 expression is up-regulated in tumor cells, PCLAF protein expression in CAFs is reduced, and their invasion and migration abilities are inhibited (Fig. S5B to H). These findings suggest that TGFB1 derived from tumor cells negatively regulates PCLAF expression in fibroblasts, thereby modulating HNSCC malignant behavior.

To determine the clinical value of PCLAF^+^ fibroblasts, we further constructed a prognostic model for the HNSCC patient cohort (Fig. S6A to D). Based on the activity levels of 12 key genes in the C7 PCLAF^+^ fibroblast subtype, HNSCC patients have been divided into 2 categories: a high-PFRS (PCLAF^+^ fibroblast risk score) group and a low-PFRS group. The findings revealed that patients in the high-PFRS group exhibited significantly reduced overall survival (Fig. S6E to H). The gene set variation analysis enrichment analysis results for differentially genes comparing the high-PFRS group and low-PFRS group are provided in Fig. S6I to K. The results showed that the low-PFRS group was closely related to immune regulation and other aspects.

### GDF15 induced up-regulation of PCLAF expression through the transcription factor IRF5

To further explore the transcriptional mechanisms underlying PCLAF up-regulation in HNSCC, we first identified interferon regulatory factor 5 (IRF5) as a potential transcription factor for PCLAF using the JASPAR database. To investigate the transcriptional regulation of PCLAF by IRF5, we analyzed the human PCLAF promoter region and identified 3 predicted IRF5 binding sites (TFBS1 to TFBS3) located upstream of the transcription start site (Fig. S7A). After transfection of 3×flag-tagged IRF5 vector, IRF5 was successfully overexpressed (Fig. S7B and C). Compared with the pGL3-Basic+pcDNA3.1–3xflag group, the luciferase activity of the h-PCLAF (Full)+pcDNA3.1–3xflag group and h-PCLAF (Full)+h-IRF5 group was enhanced, and the h-PCLAF (Full)+h-IRF5 group was enhanced more significantly (Fig. S7D). These data imply that IRF5 directly interacts with the PCLAF promoter and has a function in PCLAF transcription. Mutational analysis of the binding sites demonstrated decreased luciferase activity in the h-PCLAF (Mut1)+pcDNA3.1–3xflag and h-PCLAF (Mut2)+pcDNA3.1–3xflag groups compared to the h-PCLAF (Full)+pcDNA3.1–3xflag group, while the h-PCLAF (Mut3)+pcDNA3.1–3xflag group showed no change. Similarly, luciferase activity was reduced in the h-PCLAF (Mut1)+h-IRF5 and h-PCLAF (Mut2)+h-IRF5 groups compared to the h-PCLAF (Full)+h-IRF5 group, with no change observed in the h-PCLAF (Mut3)+h-IRF5 group. These results show that IRF5 binds to the PCLAF promoter’s TFBS1 and TFBS2, regulating PCLAF transcription. chromatin immunoprecipitation quantitative polymerase chain reaction (CHIP-qPCR) assays further confirmed that IRF5 directly binds to the PCLAF promoter in both FaDu and Tu212 cells (Fig. S7E and F). These data demonstrate that IRF5 directly targets the PCLAF promoter and acts as a transcriptional activator in HNSCC. Additionally, the study revealed that the IRF5-specific inhibitor IRF5-IN-1 markedly down-regulated PCLAF expression and promoted apoptosis in HNSCC cells. Importantly, the addition of recombinant IRF5 protein (HY-P70573) reversed this effect (Fig. S8A and B).

Macrophage inhibitory factor-1, also known as growth differentiation factor 15 (GDF15), belongs to the TGF-β superfamily and is linked to the activation of many tumor-promoting pathway [17–19], but has not been studied in depth in HNSCC. To investigate whether GDF15 directly regulates IRF5, we performed coimmunoprecipitation experiments in HNSCC cells and confirmed a direct interaction between the 2 proteins (Fig. S8C). Nuclear fraction analysis further demonstrated that IRF5 predominantly localized to the nucleus in HNSCC tumor tissues compared to normal controls, and GDF15 overexpression enhanced this nuclear enrichment, whereas GDF15 knockdown reduced nuclear IRF5 levels (Fig. S8D). Consistently, both mRNA and protein expression of IRF5 were significantly up-regulated in HNSCC and further enhanced by GDF15 overexpression, while GDF15 silencing markedly suppressed IRF5 levels (Fig. S8E and F). These results suggest that GDF15 positively regulates IRF5 at both transcriptional and posttranscriptional levels. These findings support a model in which GDF15 promotes IRF5 nuclear localization and transcriptional activity to drive PCLAF expression and inhibit apoptosis in HNSCC cells.

### GDF15 interacted with its receptor GFRAL to enhance oxidative stress in tumor cells and mediate the malignant behavior of HNSCC

**Fig. 3.**
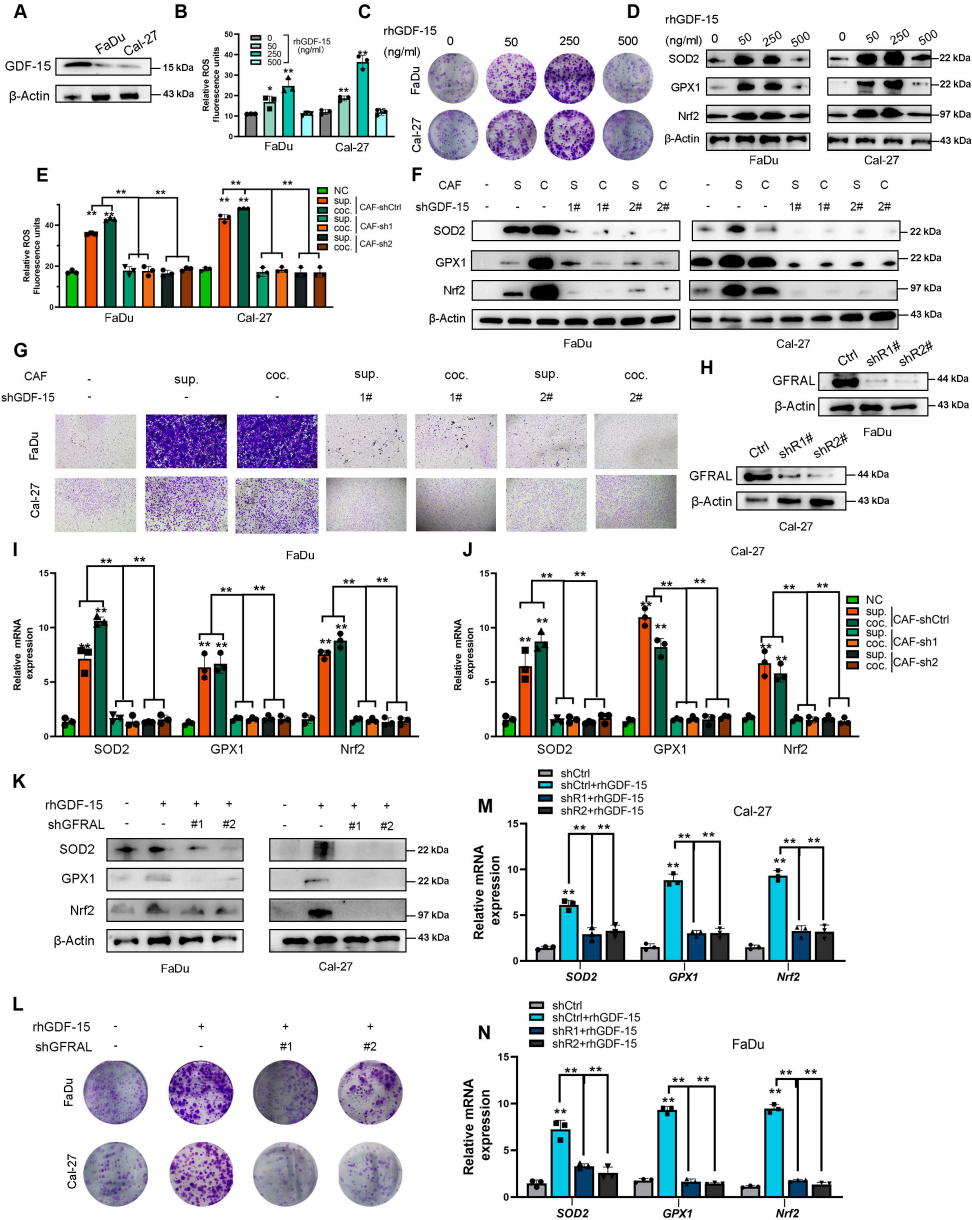
GDF15 secreted by CAFs promoted oxidative stress and malignancy in HNSCC cells via the GFRAL. (A) Protein expression of GDF15 in FaDu and Cal-27 cells. (B) Relative intracellular reactive oxygen species (ROS) levels in FaDu and Cal-27 cells treated with various concentrations of recombinant human GDF15 protein (rhGDF15) (0, 50, 250, and 500 ng/ml). (C) Representative images of colony formation assays following rhGDF15 treatment in FaDu and Cal-27 cells. (D) Western blot (WB) detection of SOD2, GPX1, and Nrf2 protein levels in response to rhGDF15 treatment. (E) Quantification of ROS levels in FaDu and Cal-27 cells cultured in CAF-conditioned medium (sup.) or cocultured with CAFs (coc.), with or without GDF15 knockdown (sh1 and sh2) in CAFs. (F) WB analysis of SOD2, GPX1, and Nrf2 expression in cells treated as in (E). (G) Transwell assays evaluate cell migration in FaDu and Cal-27 cells under the indicated conditions. (H) WB shows GFRAL expression in FaDu and Cal-27 cells with or without GFRAL knockdown (shR1# and shR2#). (I and J) qRT-PCR analysis of SOD2, GPX1, and Nrf2 mRNA expression in FaDu (I) and Cal-27 (J) cells treated as indicated in (E). (K) WB of antioxidant proteins in FaDu and Cal-27 cells treated with rhGDF15 ± GFRAL knockdown. (L) Colony formation assay under the same conditions as in (K). (M and N) qRT-PCR quantification of oxidative stress marker genes in Cal-27 (M) and FaDu (N) cells after GFRAL knockdown and rhGDF15 stimulation. Data are presented as mean ± SD from 3 independent experiments. **P < 0.01 by one-way analysis of variance with post hoc test.

**Fig. 4.**
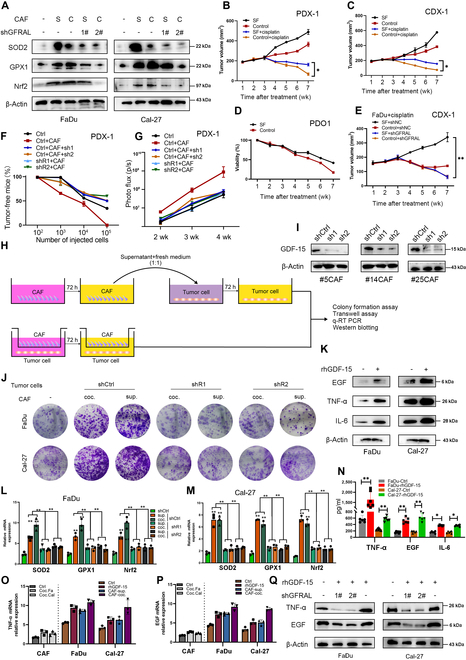
GDF15 elevated oxidative stress and promoted secretion of TNF-α, EGF, and IL-6. (A) WB analysis of oxidative stress markers (SOD2, GPX1, and Nrf2) in FaDu and Cal-27 cells cocultured with CAFs or their supernatants, with or without GFRAL knockdown (1# and 2#). (B and C) Tumor volume curves in PDX-1 and CDX-1 models show effects of sodium fluoride (SF)-induced oxidative stress and cisplatin treatment. (D) PDO1 cell viability over time with or without SF treatment. (E) Tumor volume of CDX mice injected with FaDu cells under the indicated conditions. (F) Tumor regression rates were assessed by limiting dilution in PDX-1 mice injected with FaDu cells or GFRAL-knockdown FaDu cells cocultured with CAFs or GDF15-deficient CAFs. (G) In vivo bioluminescent imaging (photonic flux) of tumor progression in PDX-1 mice across 2, 3, and 4 weeks. (H) Experimental schematic of coculture and conditioned medium transfer systems between CAFs and tumor cells. (I) WB shows GDF15 expression in CAF clones with shRNA knockdown. (J) Representative images of colony formation assays with FaDu and Cal-27 cells cultured with CAFs (coc.) or CAF supernatants (sup.), in the presence or absence of GFRAL knockdown. (K) WB analysis of EGF, TNF-α, and IL-6 expression in FaDu and Cal-27 cells following rhGDF15 stimulation. (L and M) qRT-PCR quantification of SOD2, GPX1, and Nrf2 mRNA expression in FaDu and Cal-27 cells under different CAF coculture conditions. (N) Enzyme-linked immunosorbent assay quantification of TNF-α, EGF, and IL-6 protein levels in cell culture supernatants. (O and P) qRT-PCR analysis of TNF-α and EGF mRNA expression in HNSCC cells with or without GDF15 stimulation or CAF coculture. (Q) WB shows that GFRAL knockdown suppressed rhGDF15-induced TNF-α and EGF expression in FaDu and Cal-27 cells. Data represent mean ± SD from 3 independent experiments. *P < 0.05, **P < 0.01 by one-way analysis of variance or unpaired *t* test as appropriate.

Based on the above results, we further calculated the expression level of GDF15 in HNSCC cells in single-cell data. Initially, we performed InferCNV analysis on epithelial cells in the sample to obtain tumor cells (Fig. S9A) and annotated 8 different tumor cell subtypes (Fig. S9B). Next, according to the expression level of GDF15, we divided HNSCC tumor cells into high GDF15 tumor cells and low GDF15 tumor cells. In particular, Fig. S9C and D show that most tumor cells exhibited low GDF15 expression. This was consistent with our experimental results (Fig. 3A). However, it was worth mentioning that we found that GDF15 showed higher expression in CAFs (Fig. 4I).

Recent studies have linked GDF15 to oxidative stress and drug resistance in cancer cells. However, analysis of oxidative-stress-related pathways showed that tumor cells with high GDF15 expression only exhibited a weak oxidative stress advantage (Fig. S9E to G). This suggested that the GDF15-mediated enhancement of HNSCC oxidative stress activity might originate from exogenous signals rather than GDF15 secreted by tumor cells themselves. To explore this issue, we treated GDF15-deficient HNSCC cells with different concentrations of recombinant human GDF15 protein (rhGDF15). The results showed that the oxidative stress and clonogenic ability of HNSCC cells increased in a concentration-dependent manner, with the most significant effect at a concentration of 250 ng/ml, and reversed at a concentration of 500 ng/ml (Fig. 3B to D). Next, we evaluated the paracrine effect of CAF-derived GDF15 on tumor cells. HNSCC cells treated with CAF medium or cocultured with CAFs exhibited enhanced oxidative stress and migration abilities, whereas these abilities were significantly reduced after silencing of GDF15 in CAFs (Fig. 3E to G, I, and J).

To explore the role of GDF15 receptor GDNF family receptor alpha like (GFRAL) in this mechanism, we established and validated a GFRAL knockdown HNSCC cell line (Fig. 3H). The results showed that silencing GFRAL significantly reversed the increased oxidative stress levels and HNSCC cell proliferation induced by rhGDF15 (Fig. 3K to N). Consistent with this, coculture of HNSCC cells with CAFs led to increased oxidative stress levels, which were reduced after knockdown of GFRAL (Fig. 4A). These results suggested that CAF-derived GDF15 enhanced oxidative stress and malignant phenotypes of HNSCC cells through GFRAL-dependent activation of antioxidant response genes.

### GDF15 elevated oxidative stress and increased secretion of TNF-α, EGF, and IL-6, contributing to poor tumor prognosis

To evaluate the impact of oxidative stress on tumor progression and therapy resistance in vivo, we employed 3 independent mouse models: patient-derived xenograft (PDX), cell-derived xenograft (CDX), and patient-derived organoid (PDO). In the PDX model, pretreatment with sodium fluoride (SF) to induce oxidative stress increased tumor burden and diminished cisplatin responsiveness (Fig. 4B). A similar trend was observed in the CDX model using Cal-27 cells (Fig. 4C). In the PDO model, SF treatment enhanced tumor cell viability over time (Fig. 4D). Importantly, GFRAL knockdown reversed SF-induced chemoresistance and reduced tumor volume (Fig. 4E). In vivo limiting dilution assays revealed that GDF15 silencing in CAFs markedly reduced tumor-initiating capacity in PDX-1 mice, as evidenced by higher tumor-free survival (Fig. 4F). Bioluminescence imaging confirmed a lower tumor burden in mice injected with GDF15-deficient CAFs (Fig. 4G). Similar results were obtained by knocking down GFRAL in tumor cells.

A schematic illustration of the CAFs–tumor coculture or supernatant transfer system is shown in Fig. 4H. Western blot (WB) validated successful GDF15 knockdown in multiple CAF clones (Fig. 4I). Notably, knockdown of the GFRAL gene in HNSCC cells significantly reduced the clonogenicity and oxidative stress of tumor cells induced by coculture with CAFs or treatment with CAF supernatant (Fig. 4J, L, and M). Mechanistically, rhGDF15 stimulation induced secretion of tumor necrosis factor-α (TNF-α), epidermal growth factor (EGF), and interleukin-6 (IL-6) in HNSCC cells (Fig. 4K and N). Further studies revealed that adding rhGDF15 to HNSCC cell cultures or coculturing with CAFs or their supernatants markedly increased the secretion of TNF-α and EGF (Fig. 4O and P). Finally, GFRAL knockdown in tumor cells inhibited GDF15-induced TNF-α and EGF expression, confirming GFRAL as the mediator of GDF15 downstream signaling (Fig. 4Q). These data established a paracrine CAFs–tumor loop where the GDF15–GFRAL axis governed oxidative stress, cytokine secretion, and tumor cell proliferation.

### GDF15 regulated intracellular oxidative stress via the PI3K/AKT/STAT3/TNF-α/p53/CTCF pathway

**Fig. 5.**
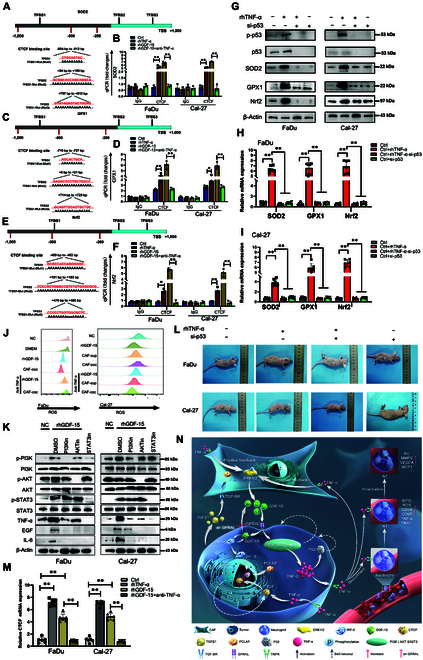
GDF15 regulated intracellular oxidative stress through the PI3K/AKT/STAT3/TNF-α/p53/CTCF pathway. (A, C, and E) Schematic diagrams of the predicted CTCF binding sites in the promoter regions of SOD2 (A), GPX1 (C), and Nrf2 (E). Three transcription factor binding sites (TFBS1 to TFBS3) are indicated relative to the transcription start site, with sequences for wild-type and mutant sites shown. (B, D, and F) ChIP-qPCR quantification of CTCF enrichment on SOD2 (B), GPX1 (D), and Nrf2 (F) promoters in FaDu and Cal-27 cells following treatment with control, recombinant human TNF-α protein (rhTNF-α), rhGDF15, or rhGDF15+anti-TNF-α. (G) WB analysis of p53 and oxidative-stress-related proteins in FaDu and Cal-27 cells under indicated treatments. (H and I) qRT-PCR quantification of SOD2, GPX1, and Nrf2 mRNA levels in FaDu (H) and Cal-27 (I) cells treated as above. (J) Flow cytometry analysis of intracellular ROS levels in FaDu and Cal-27 cells under the indicated conditions: rhGDF15, CAF supernatant (CAF-sup.), CAF coculture (CAF-coc.), and anti-TNF-α treatment. (K) WB revealed that GDF15 modulated TNF-α, EGF, and IL-6 expression via the PI3K/AKT/STAT3 pathway. (L) Tumor growth images of FaDu and Cal-27 xenografts treated with rhTNF-α or rhTNF-α+si-p53. (M) qRT-PCR analysis of CTCF mRNA expression in FaDu and Cal-27 cells under rhGDF15, rhTNF-α, or rhGDF15+anti-TNF-α conditions. Data are presented as mean ± SD of 3 independent experiments. *P < 0.05, **P < 0.01 by one-way analysis of variance or *t* test. (N) Mechanism diagram reveals that GDF15 derived from CAFs induces oxidative stress in HNSCC through the PI3K/AKT/STAT3 signaling axis.

To investigate how GDF15-induced oxidative stress was transcriptionally regulated, we first analyzed the promoter regions of SOD2, GPX1, and Nrf2 and identified multiple predicted binding sites for CTCF (Fig. 5A, C, and E). ChIP-qPCR assays confirmed enhanced enrichment of CTCF at the promoter regions of SOD2, GPX1, and Nrf2 upon treatment with rhGDF15 or TNF-α (Fig. 5B, D, and F). Notably, TNF-α blockade attenuated the CTCF binding induced by rhGDF15 treatment, suggesting that TNF-α was involved in the recruitment of CTCF to the promoters of oxidative stress response genes.

Next, we explored whether p53, a known regulator of both CTCF and oxidative stress, was implicated in this regulatory pathway. Results showed that TNF-α stimulation up-regulated p53, SOD2, GPX1, and Nrf2, while p53 knockdown attenuated these effects (Fig. 5G). Consistently, quantitative reverse transcription polymerase chain reaction (qPT-PCR) results demonstrated that TNF-α-mediated induction of antioxidant genes was attenuated by p53 knockdown (Fig. 5H and I), highlighting a p53-dependent mechanism.

To further examine the upstream cues leading to TNF-α expression and reactive oxygen species (ROS) elevation, we quantified intracellular ROS levels using flow cytometry. Treatment with rhGDF15 or conditioned media from CAFs increased ROS production in both FaDu and Cal-27 cells, while TNF-α neutralization markedly reversed this effect (Fig. 5J). WB analysis revealed that rhGDF15 activated the phosphatidylinositol-3 kinase/protein kinase B/signal transducer and activator of transcription 3 (PI3K/AKT/STAT3) pathway, accompanied by the up-regulation of TNF-α, EGF, and IL-6, which was abolished by pharmacological inhibitors (Fig. 5K).

In vivo experiments confirmed that TNF-α treatment enhanced tumor growth in both FaDu and Cal-27 xenograft models, and this effect was dependent on p53 (Fig. 5L). Finally, qRT-PCR analysis showed that rhGDF15 significantly increased CTCF mRNA expression, which was dependent on TNF-α signaling (Fig. 5M). WB confirmed increased nuclear accumulation of CTCF in response to rhGDF15 and recombinant human TNF-α protein (rhTNF-α), which was abolished by anti-TNF-α treatment (Fig. S10A). Together, these results indicated that CAF-derived GDF15 activated PI3K/AKT/STAT3 signaling, leading to TNF-α secretion and p53/CTCF-dependent transcription of antioxidant genes that contribute to tumor cell oxidative stress adaptation. The mechanism diagram of the above findings is shown in Fig. 5N.

### GDF15 promoted neutrophil recruitment and pro-tumorigenic activation via TNF-α-dependent paracrine signaling

Next, we explored the downstream inflammatory consequences of GDF15–TNF-α signaling. To ensure the validity of neutrophil-based functional assays, we first examined neutrophil viability ex vivo. Annexin V/propidium iodide staining showed that freshly isolated neutrophils remained largely viable (>50%) up to 24 h in culture (Fig. S10C). As shown in Fig. S10B, rhGDF15 treatment significantly up-regulated multiple proinflammatory and neutrophil-recruiting factors, including MCP1, CCL5, VEGFA, VEGFB, TGFB1, IL-6, IL-8, IL-10, and CSF3, compared to control. Notably, rhTNF-α also increased the expression of VEGFA, VEGFB, and TGFB1, though to a lesser extent than rhGDF15. In contrast, the expression levels of MPO, ELA2, ARG1, and ICAM1 remained unchanged in all treatment groups, indicating that rhGDF15 and rhTNF-α have selective effects on tumor-promoting cytokine expression. Neutrophil chemotaxis assays demonstrated that rhTNF-α markedly enhanced neutrophil migration toward HNSCC-conditioned media (Fig. S10D). This chemotactic activity was significantly reduced by TNFR1 and TNFR2 blockade (Fig. S10E). A comprehensive workflow illustrating the coculture, conditioned medium transfer, and neutrophil functional assays is provided in Fig. S10F. Flow cytometric analysis showed that the expression of neutrophil activation markers CD154 and CD95 was increased in HNSCC cells after exposure to GDF15 or CAF culture medium, which was reversed by TNF-α neutralization (Fig. S10G). WB confirmed that, mechanistically, CAF-derived GDF15-induced extracellular signal-regulated kinase 1/2 (ERK1/2) phosphorylation in HNSCC cells was dependent on TNF-α and EGF signaling (Fig. S10H).

To further evaluate how GDF15 affects neutrophil function, we treated neutrophils with conditioned tumor cell supernatants. We found that treatment with tumor cell supernatants conditioned with rhGDF15 or CAF-conditioned medium reduced neutrophil secretion of sTRAIL and MCP1, which are typically associated with neutrophil-mediated antitumor immunity. Notably, treatment with anti-TNF-α antibody restored the secretion of both factors (Fig. S10I), suggesting that GDF15 suppressed neutrophil immune function in a TNF-α-dependent manner. In contrast, VEGFA secretion was significantly increased in neutrophils, and TNF-α blockade abolished this increase (Fig. S10I). These findings suggested that GDF15 reprogrammed neutrophils into a pro-tumorigenic state by suppressing cellular immunity and enhancing angiogenic factors in a TNF-α-dependent manner.

In vivo bioluminescence imaging showed that rhGDF15 stimulation increased tumor burden in FaDu xenografts coinjected with mesenchymal stem cells (MSCs), and similar results were observed in GDF15 knockout animals (Fig. S10J). In addition to increased tumor growth, coinjection of neutrophils with MSCs also promoted lung metastasis of tumors in mice compared with other groups (Fig. S10K). Together, these results suggested that GDF15 promoted neutrophil recruitment and activation through a TNF-α-dependent paracrine loop, ultimately promoting tumor progression.

### Irinotecan and risperidone were regulators of GDF15–GFRAL signaling

**Fig. 6.**
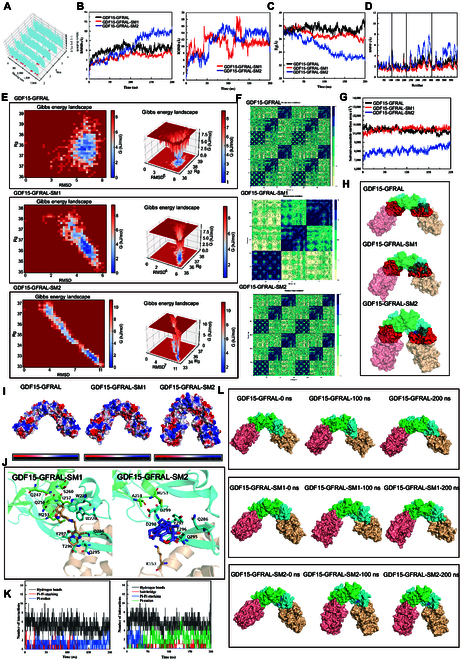
Molecular dynamics simulation of GDF15–GFRAL. (A) Food and Drug Administration drug docking energy map. (B) Root mean square deviation variations of GDF15–GFRAL within GDF15, GFRAL, GDF15–GFRAL–SM-1, and GDF15–GFRAL–SM-2 systems (left). Root mean square deviation fluctuations of ligands relative to the protein in GDF15–GFRAL–SM-1 and GDF15–GFRAL–SM-2 systems (right). (C and D) Temporal changes in radius of gyration (Rg) and root mean square fluctuation (RMSF) for GDF15–GFRAL, GDF15–GFRAL–SM-1, and GDF15–GFRAL–SM-2. (E) Free energy landscapes (FELs) of GDF15–GFRAL, GDF15–GFRAL–SM-1, and GDF15–GFRAL–SM-2. The color gradient from blue to red indicated increasing free energy; densely clustered blue points represent the lowest-energy states, corresponding to the most stable protein conformations. (F) Dynamic cross-correlation matrices of GDF15–GFRAL, GDF15–GFRAL–SM-1, and GDF15–GFRAL–SM-2. Lighter colors denote stronger negative correlations, whereas darker colors indicate stronger positive correlations. (G) Solvent-accessible surface area (SASA) analyses for GDF15–GFRAL, GDF15–GFRAL–1261SM-1, and GDF15–GFRAL–SM-2. Upon protein–protein interaction, amino acid residues at the interface became closer, reducing solvent exposure; thus, a smaller SASA indicated tighter binding. (H) Binding interface between GDF15 and GFRAL with interaction regions highlighted in red. (I) Electrostatic potential maps of GDF15–GFRAL, GDF15–GFRAL–SM-1, and GDF15–GFRAL–SM-2 complexes. (J) Binding conformations of SM-1 and SM-2 within the GDF15–GFRAL complex. (K) Interaction force distribution maps of SM-1 and SM-2 with GDF15–GFRAL. (L) Structural evolution of GDF15–GFRAL, GDF15–GFRAL–SM-1, and GDF15–GFRAL–SM-2 proteins throughout the simulation.

To investigate the molecular dynamics properties of GDF15–GFRAL, we utilized docking energy maps obtained for Food and Drug Administration-approved small-molecule drugs (Fig. 6A). As shown in Fig. 6B (left), GDF15–GFRAL, GDF15–GFRAL–irinotecan (SM-1), and GDF15–GFRAL–risperidone (SM-2) all underwent marked conformational changes before reaching equilibrium around 150 ns. Further analysis (Fig. 6B, right) revealed that SM-1 reached a stable interaction mode with the protein after 160 ns, while SM-2 reached a stable bound conformation after approximately 150 ns. Furthermore, the radius of gyration (Fig. 6C) decreased after SM-1 and SM-2 bound, indicating a decrease in the overall internal mobility of the protein. Root mean square fluctuation plots (Fig. 6D) revealed a marked decrease in the flexibility of GDF15 (chains A and B) upon ligand binding. SM-1 binding also reduced the flexibility of GFRAL (chains C and D), while SM-2 binding appeared to increase its flexibility. After 200 ns of simulation, all 3 systems converged to a relatively low-energy, structurally stable state (Fig. 6E). Furthermore, SM-1 and SM-2 binding markedly enhanced the motional correlation between GDF15 and GFRAL (Fig. 6F), indicating that small-molecule binding promotes a tighter protein–protein interaction.

The solvent-accessible surface area plot (Fig. 6G) showed that SM-1 binding did not markedly alter the GDF15–GFRAL interface, while SM-2 binding led to a tighter interaction interface (Fig. 6H). Compared to the other systems, SM-2 binding led to a slight accumulation of negative charge on the surface of the GDF15–GFRAL–SM-2 complex. The altered surface charge distribution at the GDF15–GFRAL interaction interface (Fig. 6I) suggested that small-molecule binding modulated the binding mode between the 2 proteins. Comparison of the bound states (Fig. 6J) revealed that SM-2 enhances the GDF15–GFRAL interaction by contacting residue K153 (chain D). molecular mechanics/generalized Born surface area calculations indicated that the binding free energies of SM-1 and SM-2 to the GDF15–GFRAL complex were −73.59 and −67.69 kcal/mol, respectively. Interaction spectrum analysis (Fig. 6K) demonstrated that SM-1 was primarily stabilized by hydrogen bonds and π–π stacking interactions, while SM-2 was primarily stabilized by hydrogen bonds, salt bridges, and π–cation interactions. Figure 6L shows the temporal structural evolution of the protein system during the simulation, demonstrating its gradual stabilization over time.

### SM-2 inhibited the malignant behavior of tumor cells

To functionally dissect pharmacological modulators of the GDF15 axis, we tested 2 small molecules in cocultured FaDu and Cal-27 HNSCC cells. SM-1 treatment significantly increased GDF15 protein levels (Fig. S11A), accompanied by elevated intracellular ROS (Fig. S11B) and increased transcription of oxidative stress response genes Nrf2 and SOD2 (Fig. S11C). Functionally, SM-1 enhanced colony-forming ability in both cell lines in a dose-dependent manner (Fig. S11D and E) and promoted cell migration under both CAF conditions (Fig. S11F). However, when GDF15 was silenced in CAFs, the pro-migratory effect of SM-1 was significantly attenuated, indicating that SM-1 exerts its tumor-promoting effects through a GDF15-dependent mechanism.

In contrast, SM-2 treatment down-regulated GDF15 expression in cocultured tumor cells (Fig. S11G), suppressed ROS levels (Fig. S11H), and reduced expression of Nrf2 and SOD2 (Fig. S11I). SM-2 also potently inhibited clonogenicity (Fig. S11J and K) and tumor cell migration under both CAF conditions (Fig. S11L). Importantly, the inhibitory effects of SM-2 on migration were largely reversed by supplementation with rhGDF15, further confirming the central role of GDF15 in mediating the observed phenotypes.

These findings identify SM-1 as a GDF15 activator that exacerbates oxidative stress and malignant behaviors, and SM-2 as a GDF15 suppressor with therapeutic potential to mitigate tumor progression.

## Discussion

Even though breakthroughs in immune checkpoints have markedly improved the survival rate of HNSCC patients, more patients are unable to benefit from current treatments due to poor risk management and limited personalized treatment strategies [20]. Fibroblasts are incredibly adaptable and multipurpose cells that actively aid in the spread of cancer by interacting intricately with other TME cell types [21]. This study used multi-omics technologies to fully characterize the dynamic changes and biological characteristics of fibroblasts during the evolution of normal tissue into HNSCC, providing a wealth of insights into the functional characteristics and molecular mechanisms driving fibroblast–tumor cell interactions, thereby revealing potential therapeutic targets. Our study especially underscores the potential application of GDF15 in HNSCC, particularly its regulatory role in oxidative stress within tumor cells.

We identified, for the first time, a subtype of fibroblasts in HNSCC characterized by high expression of PCLAF. As a classic proliferating cell nuclear antigen clamp-associated factor, previous studies have linked PCLAF to malignant proliferation in tumor tissues [21]. PCLAF can interact with various cell cycle regulators (such as cyclin-dependent kinases), which accelerate the S-phase entry of tumor cells and bypass cell cycle checkpoints to promote rapid tumor growth [22]. In addition, PCLAF is involved in DNA damage repair by interacting with proliferating cell nuclear antigen [23], which is advantageous for cancer cells’ resistance and survival in genotoxic treatments like radiation and chemotherapy. This PCLAF^+^ fibroblast subtype also showed a high degree of stemness, which may be related to PCLAF promoting self-renewal by enhancing the nuclear translocation of β-catenin and its transcriptional activity [24,25]. This suggests a role for PCLAF in tumor–stroma crosstalk and emphasizes its potential as a target for therapy for disrupting the supportive microenvironment.

Interestingly, our studies revealed that a subtype of fibroblasts with high PCLAF expression tended to display stromal fibroblastic features. Previous studies have found that CAFs expressing high levels of PCLAF can enhance the synthesis of collagen and fibronectin, extracellular matrix components, contributing to a supportive niche for tumor cells [26]. Furthermore, PCLAF may control matrix metalloproteinase secretion, promoting invasion and metastasis by facilitating extracellular matrix remodeling and breakdown [27].

It is worth mentioning that we observed that TGFB1 treatment could inhibit PCLAF expression in tumor cells. This may be related to the fact that TGFB1 induces growth inhibitory responses through suppressor of mothers against decapentaplegic (SMAD)-dependent and nonclassical signaling pathways, interfering with the expression of cell cycle regulatory factors including PCLAF [28,29]. However, due to experimental limitations, we were unable to further explore whether this effect was mediated by transcriptional repression, protein destabilization, or posttranscriptional regulation. Future studies using SMAD knockdown, promoter detection, or pathway-specific inhibitors are necessary to clarify the mechanistic association between TGFB1 and PCLAF. It is worth noting that although our data showed that the TGF-β family member GDF15 can enhance PCLAF transcriptional activity by regulating IRF5, we did not directly assess whether IRF5 activation (e.g., by phosphorylation or nuclear localization) is functionally dependent on GDF15 signaling. Further mechanistic studies are needed to determine whether GDF15 regulates IRF5 activity through classical activation pathways (e.g., TBK1/IKK-mediated phosphorylation or nuclear shuttling).

As a key marker of oxidative stress, GDF15 possesses independent prognostic value in cancer and cardiovascular and aging-related diseases [18,30,31]. However, the regulatory role between GDF15 and oxidative stress in HNSCC is still unclear. By integrating omics approaches and experimental design, the research team confirmed for the first time that GDF15 promotes the secretion of proinflammatory factors TNF-α and IL-6 via the classical PI3K/AKT/STAT3 pathway, which subsequently induces the up-regulation of P53 and CTCF expression. CTCF, a transcription factor for various oxidative stress markers, enhances the transcriptional activity and expression levels of SOD2, GPX1, and NRF2. ROS buildup and increased oxidative stress in HNSCC cells result from this, thereby accelerating tumor proliferation and invasion and promoting tumor chemotherapy resistance. It is important to point out that the fold enrichment of CTCF binding at some genomic loci appears to be modest (<5-fold), which may reflect the relatively weak occupancy of CTCF at distal regulatory elements compared to its canonical promoter targets. In addition, due to the limited chromatin material obtained from fluorescence-sorted CAF subtypes, we were unable to perform ChIP assays in parallel against established positive control loci (e.g., H19 or MYC) and to perform immunoglobulin G controls and input quantification at each locus. Although normalization was performed using input DNA and negative control regions, the lack of a complete immunoprecipitation control may reduce interpretability. Future studies with higher input material, including genome-wide CTCF analysis with stringent internal controls, should be performed to further validate binding specificity. Previous studies have identified p53 response elements in the promoters of SOD2 and GPX1, suggesting a functional interaction between p53 and these oxidative stress genes [32–34]. However, the binding of p53 to the promoter regions of these genes was not directly assessed in this study. Promoter-luciferase assays and chromatin binding assays (e.g., electrophoretic mobility shift assay and ChIP-seq) are needed in the future to verify direct transcriptional regulation between the two. Although our study did not directly explore the stimulatory factors that drive GDF-15 expression, it still innovatively explored how GDF15 affects tumor cell oxidative stress in HNSCC through the PI3K/AKT/STAT3 pathway, providing a new perspective for understanding the biological significance of GDF15 in this context. In the future, studies combining cytokine stimulation in CAFs and regulation of the unfolded protein response pathway will help to elucidate the dynamic regulatory mechanisms of GDF15 in the TME.

The activation of the classical inflammatory pathway PI3K/AKT/STAT3 establishes a foundation for the excessive secretion of pro-inflammatory factors. A patient’s tumor burden eventually rises as a result of prolonged chronic inflammation, which causes an imbalance in antioxidant levels and an excess of oxygen species that are reactive in nature. This indicates that the overactivation of oxidative stress in tumor cells is closely linked to a persistent inflammatory imbalance. One of the innate immune system’s essential components, the role of neutrophils in malignant tumors has garnered considerable attention in recent years [35,36]. The investigation revealed that elevated expression of GDF15 promoted neutrophil immune infiltration, ultimately facilitating the invasion and metastasis of HNSCC cells. This regulatory mechanism may be associated with the TNF-α-mediated ERK1/2 signaling pathway. It is also important to note that we only used CD95 and CD154 to assess neutrophil activation, and these markers may not be sufficient to fully describe N1/N2 polarization. Furthermore, while our results suggest that CAF-derived signals are able to enhance neutrophil recruitment, we also acknowledge that chemokines secreted by tumor cells, such as CXCL1 and IL-8, may also play a role. Previous studies have shown that HNSCC tumor cells produce high levels of chemokines and promote myeloid cell infiltration [37,38]. Although these factors were not assessed in our current model due to experimental limitations, future studies combining chemokine analysis and neutralization experiments will be critical to distinguish the specific contributions of CAFs and tumor cells to neutrophil chemotaxis.

Finally, we report for the first time the dual regulatory roles of 2 small-molecule compounds, SM-1 and SM-2, in modulating the GDF15–GFRAL signaling pathway. Specifically, SM-1 enhances the stability of the GDF15–GFRAL complex, thereby activating a GDF15-mediated, tumor-promoting oxidative stress response. This compound may serve as a valuable chemical probe for investigating the physiological functions of GDF15. In contrast, SM-2 functions as a novel small-molecule inhibitor that disrupts GDF15–GFRAL interactions at both structural and functional levels. By remodeling the interfacial electrostatic landscape and altering conformational dynamics, SM-2 attenuates CAF-mediated paracrine signaling and the associated pro-tumorigenic oxidative stress. These properties position SM-2 as a promising lead compound for therapeutic targeting of GDF15-driven malignancies. Further studies are warranted to assess its in vivo efficacy and pharmacokinetics, as well as to pursue structure-based optimization to enhance its potency and specificity.

## Conclusion

This work clarifies GDF15’s critical function in fibroblast–tumor cell interactions in 3 key ways: First, GDF15 promotes the growth and invasion of HNSCC cells by up-regulating PCLAF expression via IRF5. Second, GDF15 binds to its receptor GFRAL and activates a chronic inflammatory response via the PI3K/AKT/STAT3 pathway, which induces an imbalance in oxidative stress, resulting in increased tumor burden and drug resistance. Third, GDF15 modulates the ERK1/2 pathway by regulating TNF-α, leading to enhanced neutrophil infiltration and the onset of lung metastasis. Finally, the small-molecule compound SM-2 inhibits the malignant behavior of tumor cells by blocking the binding of GDF15–GFRAL. These findings offer a novel perspective for a more profound understanding of the molecular mechanisms underlying HNSCC and establish a foundation for potential therapeutic strategies. They underscore the clinical applicability of interventions targeting GDF15 and its downstream signaling pathways in the treatment of HNSCC.

## Materials and Methods

### Sources and processing of single-cell data

The single-cell RNA sequencing (scRNA-seq) data of HNSCC were collected from Gene Expression Omnibus database (https://www.ncbi.nlm.nih.gov/geo/) GSE181919. The “Seurat” program (v4.3.0) was used to analyze scRNA-seq data [39,40]. Initially, the DoubletFinder package (v2.0.3) was utilized to remove potential doublet cells and filter out low-quality cells [41].

### AUCell analysis

We used the scgmt (v0.0.3, https://github.com/ZhaoLabs-SJTU/scgmt) package to calculate the signature activity score. The method parameter selected by this package was AUCell. AUCell is a method for identifying cells in scRNA-seq data that had genes that were active. The “activity” of each cell’s genes was produced by AUCell using a set of genes as input.

### Intercellular communication

Using the CellChat R package (v1.6.1) [42], cell–cell interactions between various cell types were predicted. We examined the patterns of incoming and outgoing signals as well as the strength of each receptor–ligand interaction. Receptor–ligand pairs were examined, and signaling pathways were evaluated.

### Spatial transcriptomic data analysis

The spatial transcriptomics dataset was obtained from the public repository of 10x Genomics (https://www.10xgenomics.com/resources/datasets/human-melanoma-if-stained-ffpe-2-standard). Data preprocessing was performed using the Seurat R package (v4.3.0). Gene expression data were normalized and log-transformed using the “SCTransform” function. Dimensionality reduction was achieved via principal components analysis (PCA) using the “RunPCA” function. Subsequently, transcriptionally similar spatial clusters were identified using the “FindNeighbors” and “FindClusters” functions.

To infer the spatial distribution of different cell populations within tissue sections, we employed the robust cell type decomposition method, using an integrated scRNA-seq reference dataset. Additionally, we conducted a comprehensive analysis of the spatial expression patterns of key genes and signaling pathways to construct a functional landscape of the tissue microenvironment [43].

### Primary fibroblasts

In the same patient, CAFs were obtained from fresh human tissues, while NFs were isolated from the noncancerous area at least 5 cm from the outer tumor edge. Within 30 min after excision, tissues from HNSCC and matched normal tissues were extracted. Fresh tissues were immediately preserved in Dulbecco’s modification of Eagle’s medium supplemented with 10% fetal bovine serum and antibiotics before being sent on ice to the laboratory. The MSCs (bone marrow MSCs) were kindly supplied by Professor Dai Hanren of Anhui Medical University’s School of Pharmacy.

### Flow cytometry analysis

The surface expression of ROS, CD154, and CD95 by HNSCC cells and neutrophils treated with the appropriate conditions was evaluated using flow cytometry analysis.

The other methods can be found in the Materials and Methods section of the Supplementary Materials.

## Ethical Approval

This study was approved by the Ethical Committee of the First Affiliated Hospital of Anhui Medical University (Ethics Approval No. PJ2023-12-45).

## Data Availability

The GEO and TCGA repositories make the single-cell sequencing datasets created and/or examined during this investigation openly accessible. The primary paper and the Supplementary Materials contain all of the unique contributions made throughout this investigation. The associated authors can be contacted with questions about the original data.
