# Supplementary material for: Cancer-Associated Fibroblast-Derived GDF15 Induces Oxidative Stress and Neutrophil Infiltration in Head and Neck Squamous Cell Carcinoma through the PI3K/AKT/STAT3 Axis Cascade
Source: Research (Wash D C). 2025 Sep 30;8:0901. doi: 10.34133/research.0901 (PMC12480759; doi:10.34133/research.0901)
Supplement: Supplementary 1 — Materials and Methods Figs. S1 to S11 Table S1 [file research.0901.f1.zip › Supplementary Figure 5.pdf]

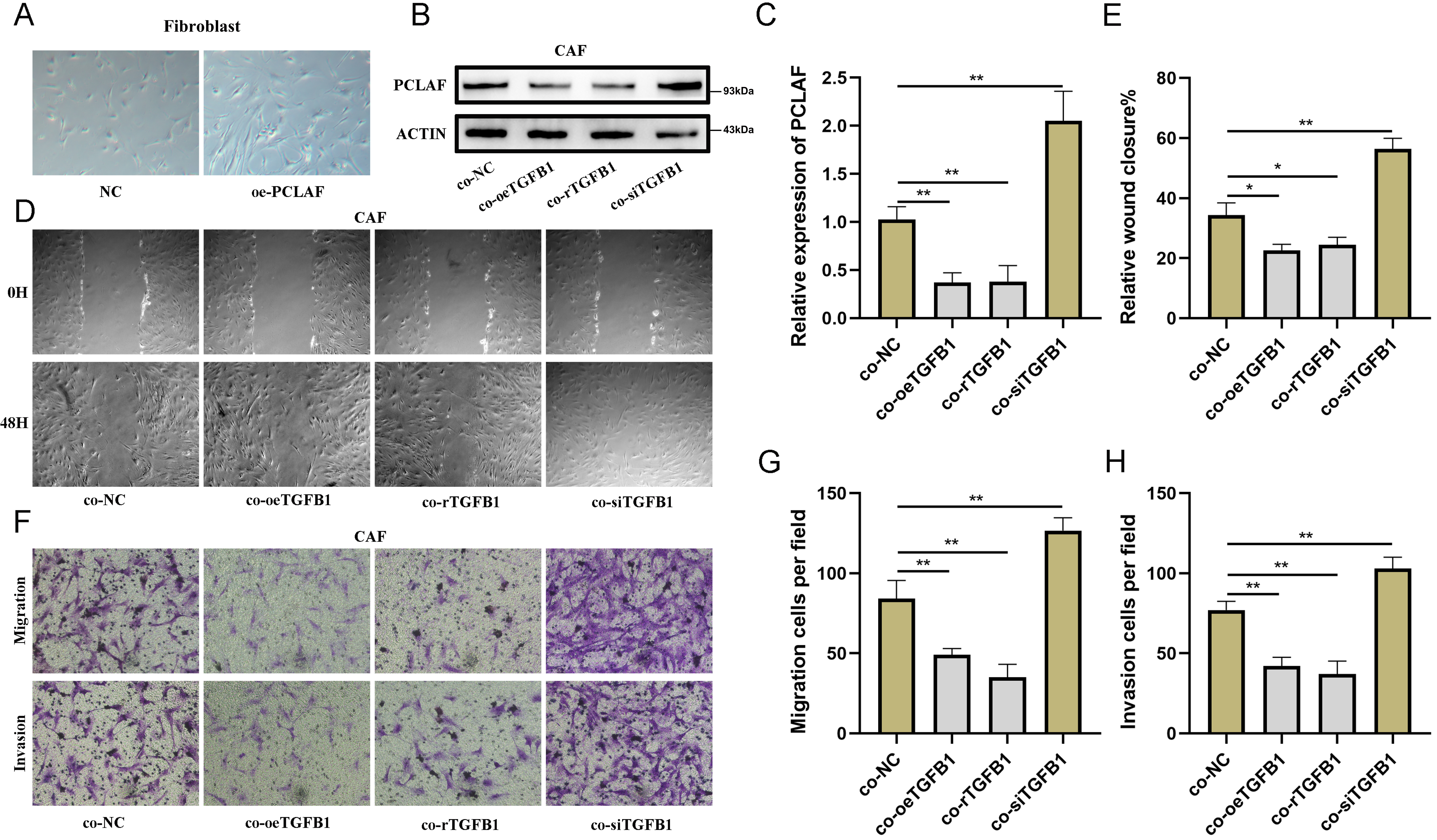

A
Fibroblast
NC
oe-PCLAF
B
CAF
PCLAF
93kDa
ACTIN
43kDa
co-NC
co-oeTGFB1
co-rTGFB1
co-siTGFB1
C
Relative expression of PCLAF
2.5
2.0
1.5
1.0
0.5
0.0
**
E
Relative wound closure%
80
60
40
20
0
*
D
CAF
0H
48H
F
Migration
Invasion
G
Migration cells per field
150
100
50
0
H
Invasion cells per field
