# Supplementary material for: Cancer-Associated Fibroblast-Derived GDF15 Induces Oxidative Stress and Neutrophil Infiltration in Head and Neck Squamous Cell Carcinoma through the PI3K/AKT/STAT3 Axis Cascade
Source: Research (Wash D C). 2025 Sep 30;8:0901. doi: 10.34133/research.0901 (PMC12480759; doi:10.34133/research.0901)
Supplement: Supplementary 1 — Materials and Methods Figs. S1 to S11 Table S1 [file research.0901.f1.zip › Supplementary Materials.docx]

**Cancer-associated fibroblast-derived GDF15 induces oxidative stress and neutrophil infiltration in head and neck squamous cell carcinoma through the PI3K/AKT/STAT3 axis cascade**

Zhijie Zhao^1†^, Huabao Cai^2,3,4†^, Zhenzhen Zhao^5†^, Xiaojing Wang^6†^, Wenyang Nie^5^, Fu Zhao^7^, Yisheng Chen^8^, Yanyu Ding^9^*, Zhiwen Luo^10^*, Zhiheng Lin^11^*, Yantao Ding^12,13^*

**Supplemental information containing the following items:**

**1.Materials and Methods**

**2.Supplementary Figures**

Supplementary Figure 1. C7 PCLAF+ Fibroblasts had high proliferative properties.

Supplementary Figure 2. Biological pathway characterization of C7 PCLAF+ Fibroblasts.

Supplementary Figure 3. Overexpression of PCLAF and TGFB1 in HNSCC.

Supplementary Figure 4. Quantitative analysis.

Supplementary Figure 5. Co-culture system demonstrating the regulatory impact of TGFB1 and PCLAF on fibroblast malignant behavior.

Supplementary Figure 6. Differential gene GSVA results for High PFRS Group and Low PFRS Group.

Supplementary Figure 7. Dual-luciferase reporter assay and CHIP validation demonstrated transcription factor IRF5 regulating PCLAF.

Supplementary Figure 8. Validation of the reciprocal interaction between GDF15 and IRF5 in regulating PCLAF in HNSCC.

Supplementary Figure 9. Visualization of tumor cells subtypes in HNSCC.

Supplementary Figure 10. GDF15 promotes TNF-α–dependent neutrophil recruitment, polarization, and tumor-promoting activity in HNSCC.

Supplementary Figure 11. Validation of Small-Molecule Screening.

**3.Supplementary Table 1.**

**1.Materials and Methods**

**Data processing and cell type identification**

The “NormalizedData” function in Seurat (v4.3.0) was used to normalize the expression matrix. The “FindVariableFeatures” algorithm was used to screen the top 2000 highly variable genes (HVGs)[1, 2]. The "ScaleData" function was used to filter and standardize HVGs. Principal component analysis (PCA) was used to reduce the dimensionality of each cell (as represented by the HVG). The Harmony package (v0.1.1) was used to eliminate batch effects between samples, and the first 30 principal components were selected for dimensionality reduction and clustering [3]. Uniform Manifold Approximation and Projection (UMAP) projected the results for further cell type identification. [4, 5]

Utilizing the CellMarker database (http://xteam.xbio.top/CellMarker/), we obtained relevant cell markers for different cell types. Identified different cell types based on these cell markers and observed their distribution and proportion [5].

**Determination of cell CNV levels**

We used the InferCNV (v1.17.0) algorithm to evaluate the level of chromosomal copy number variation (CNV) in order to identify non-diploid tumor cells and thereby distinguish non-tumor cells from malignant tumor cells. In the analysis, endothelial cells were selected as the reference cell type for InferCNV to determine whether there were significant chromosomal copy number abnormalities in other cell populations.

**Enrichment analysis**

We utilized the "FindAllMarkers" function with default parameters based on the Wilcoxon rank-sum test to identify differentially expressed genes (DEGs) for each cell type. DEGs were selected from clusters with a LogFC value greater than 0.25. We used the clusterProfiler (v4.6.2) and SCP (v0.4.8) packages to conduct enrichment analysis on DEGs in each cell classification group. The functions of DEGs were then ascertained by performing gene set analysis of differential gene enrichment by comparing the outcomes of the differential gene expression study with the Gene Ontology database (http://www.geneontology.org/) [6]. Differential genes were assessed using characteristic pathways described in a molecular characterization database to ascertain which pathways vary the most between subgroups. Gene set enrichment analysis (GSEA) was used to rank these genes from highest to lowest fold difference [7] . Subsequently, we estimated pathway activities for individual cells using the GSEA.

**Pseudotiming of HNSCC cells**

To investigate the differentiation trajectories of fibroblasts, we applied the Slingshot algorithm (v2.6.0). Lineages were inferred by identifying clusters and constructing a minimum spanning tree between them using the getLineages function, followed by the fitting of principal curves along pseudotime with the getCurves function. The relationship between gene expression and pseudotime was modeled using a generalized additive model (GAM) with a negative binomial distribution to account for gene-specific dispersion. Finally, trajectories were visualized by mapping the fitted curves onto a UMAP embedding, providing insights into the dynamic progression of CAF subpopulations[8-10].

Cellular stemness was assessed via the CytoTRACE R package (v0.3.3), which suggested the temporal progression of cell differentiation[11].

**Intercellular communication**

Using the CellChat R package (v1.6.1)[12] , cell-cell interactions between various cell types were predicted. We examined the patterns of incoming and outgoing signals as well as the strength of each receptor-ligand interaction. Receptor-ligand pairs were examined, and signaling pathways were evaluated.

**Constructing the prognostic model for CAFs**

A large amount of transcriptomic data from the TCGA-HNSCC cohort was obtained from the TCGA database (https://portal.gdc.cancer.gov/). First, a univariate Cox regression model was used for modeling. In order to avoid multicollinearity, we then used Least Absolute Shrinkage and Selection Operator (glmnet, v4.1-6) regression (Lambda.min=0.029) to screen these genes. We computed the associated risk coefficients based on each gene's risk coefficients. Subsequently, we established a prognostic model using Multivariate Cox analysis. The risk score for each sample was calculated using the following formula: Riskscore =∑ Xi × Yi (X: coefficient, Y: gene expression level). Each patient's risk score was determined using the procedure above and divide it into different risk score group (the optimal cutoff value). Finally, a fibroblasts-related prognosis model was built[13].

**Kaplan-Meier survival**

To validate the model predictions, we generated ROC curves of the 1-, 3-, and 5-year survival rates using the survival and timeROC R packages (v0.4). Then, we calculated the area under the ROC curve (AUC). Both Kaplan-Meier survival analysis and time-dependent ROC analysis were applied to validate the model's prognostic value. The risk score scoring distribution, heatmap, and scatter plot of the survival status were utilized to further evaluate model performance [14-16].

**Cell lines and animals**

The National Collection of Authenticated Cell Cultures provided the HNSCC cell lines FaDu, Tu212, and Cal-27. In Dulbecco's Modified Eagle Medium (DMEM) (Gibco BRL, USA), the HNSCC cell lines were cultivated. The mesenchymal stem cells (MSCs) FaDu, Tu212, Cal-27, and SK-MEL-28 were cultivated at 37 °C in a humidified environment with 10% fetal bovine serum and 5% CO_2_ (Gibco BRL, USA). CAFs were obtained from HNSCC tumor tissues. CAFs were cultivated at 37°C with 5% CO_2_ in DMEM/F12 supplemented with 10% FBS and 2% penicillin/streptomycin. The Chinese Academy of Medical Sciences' Cancer Institute gave GDF15 knockout naked mice, while Anhui Medical Laboratory Animal Center in China provided the others. All were kept in particular pathogen-free circumstances. The Experimental Animal Welfare and Ethics Committee of the Institute of Health and Medicine, Hefei Comprehensive National Science Center, gave its approval to all animal research (Approval No.IHM-AP-2024-039). The National Academy of Sciences' Guide for the Care and Use of Laboratory Animals' requirements were met by every animal procedure.

**Clinical samples**

One cohort containing 8 tumor and paired normal tissues from HNSCC patients underlying surgery was used for qRT-PCR, another cohort containing 8 paired HNSCC tissues and matched normal samples was selected for western blot(WB) validation. Serum samples from eight patients with allergic dermatitis were collected to serve as ELISA controls. From 2022 to 2023, the tissues were verified by pathology at the First Affiliated Hospital's Department of Otolaryngology & Head and Neck Surgery, Anhui Medical University, China. Anhui Medical University's First Affiliated Hospital provided all of the human samples, and none of them had ever been treated with an anticancer medication. Tumor tissues and matched neighboring normal skin tissues were taken from 30 HNSCC patients who were having surgery between September 2022 and May 2024 in order to isolate CAFs and normal fibroblasts (NFs). The Institutional Review Board of Anhui Medical University's first affiliated hospital authorized the use of human samples that were archived materials collected with patients' informed permission (Approval No. Quick -P J 2023-14-88). Every human experiment was conducted in compliance with the World Medical Association's Code of Ethics (Declaration of Helsinki).

**Primary tumor cells isolation**

Primary tumor cells were isolated from human HNSCC tissues using the Human Tumor cell Isolation Kit (Miltenyi Biotec, Germany), and cell lysis was used for WB.

**Neutrophil isolation and culture**

After being separated from the peripheral blood of healthy volunteers, human neutrophils were centrifuged using the Ficoll density gradient method. After washing the pale-red granulocyte layer, a red blood cell lysis buffer (Sigma-Aldrich) was used to contaminate the erythrocyte layers. following flow cytometry using human anti-CD15 and anti-CD16 antibodies to verify the cell purity (> 90%) (BD Bioscience, San Diego, USA). The cells were then cultured in 1640 RPMI medium supplemented with 10% FBS and antibiotics alone or with 50% indicated culture supernatants from HNSCC cells. Then, cells and culture supernatants were harvested and used for subsequent experiments. For tube formation assay, the Neutrophils were cultured for 24 hours in the indicated serum-free medium, and the culture supernatants were collected.

**Neutrophils migration assay**

Neutrophil migration was measured using a 24-well transwell System with 3.0 um polycarbonate membranes (Corning, NY). 2.0×10^6^ neutrophils that had been resuspended in 100 ul of serum-free RPMI 1640 medium were placed in the top chamber. 600 ul of medium, either alone or with 50% neutrophil culture supernatants, were placed in the bottom chamber. The cells that had moved into the lower chamber were collected and counted using a hemocytometer following a two-hour incubation period at 37°C. Neutrophils that only moved in the direction of RPMI 1640 served as the negative control. By dividing the number of neutrophils moving to conditioned media by the number migrating to RPMI 1640 alone, the chemotactic index was determined.

**Measurement of reactive oxygen species (ROS) levels using DCF-DA assay**

A DCF-DA (2',7'-dichlorofluorescin diacetate) assay kit (Abcam, USA) was used to measure ROS levels in accordance with the manufacturer's instructions. on short, cells were cultivated to around 70–80% confluence after being planted on 6-well plates. Following treatment under the proper experimental conditions, cells were incubated for 30 minutes in the dark at 37°C with 10µM DCF-DA in serum-free media. To get rid of extra DCF-DA, cells were rinsed twice with cold phosphate-buffered saline (PBS). The fluorescence intensity, a marker of intracellular ROS levels, was measured using a fluorescent microplate reader with excitation and emission wavelengths set at 485 nm and 535 nm, respectively. Relative ROS levels were compared between experimental groups after the findings were adjusted to cell number.

**The experiment of colony assay, transwell assay and wound healing**

DMEM, FBS, and penicillin-streptomycin were among the reagents acquired from Sigma-Aldrich for the colony formation experiment. The cells were cultivated in DMEM supplemented with 10% FBS and 1% penicillin-streptomycin after being seeded at a low density (500 cells per well) in 6-well plates. Colonies were dyed with 0.5% crystal violet and preserved with 4% paraformaldehyde after 10–14 days. ImageJ software was used to count colonies with more than 50 cells. Cell growth and clonogenic potential were evaluated by analyzing data from the triplicate experiment.

Corning supplied the reagents for the transwell invasion and migration tests (transwell chambers with 8.0 µm pores). 2×10^4^ FaDu or Cal-27 cells were planted into the transwell's top chamber after being suspended in serum-free DMEM for the migration test. The top chamber was pre-coated with Matrigel (Corning) for the invasion assay. As a chemoattractant, 10% FBS was added to DMEM in the lower compartment. For twenty-four hours, the cells were incubated at 37°C with 5% CO_2_. 1.1% crystal violet was used to stain cells that had invaded or migrated across the membrane after they had been treated with 4% paraformaldehyde. Cells were counted under a microscope in five random fields, and the experiment was performed in triplicate.

Six-well plates were used to cultivate the transfected cells until 95% confluence was achieved. A 200μl pipette tip was used to make linear scratches on the cell monolayer, and any remaining cell debris was cleaned off with PBS. For culture, the serum-free medium was maintained. Before and after 48 hours, pictures were captured, and Image J software was used to quantify them.

**Matrigel invasion assay**

The cell invasion tests were conducted using a Transwell Matrigel Invasion System (Becton Dickinson Labware, Bedford, MA). The upper chamber contained a suspension of 3×10^4^ HNSCC cells in 200 μL of serum-free RPMI 1640 media. 500 μL of control medium or supernatants obtained from neutrophils treated under the specified conditions for a full day were placed in the lower compartment. The cells on the bottom surface were fixed, stained with 1% crystal, and counted under a microscope following a 24-hour incubation period.

**Transient transfection**

The transfections were carried out using the Lipofectamine 3000 Kit (Invitrogen Carlsbad, CA, USA) in accordance with the manufacturer's instructions. The cells were transfected with a plasmid carrying small interfering (si) RNAs after being cultivated in 6-well plates to 50% to 60% confluence. To verify the effectiveness of the transfection, the cells were isolated 48 hours after transfection and subjected to either WB or qRT-PCR.

**Lentivirus vector construction and cell infection**

GenePharma (Shanghai, China) created all of the control and recombinant lentiviral vectors. Virus solution was added to cells in the presence of 5μg/mL polypropylene (Sigma-Aldrich) to accomplish lentiviral infection. Following a 72-hour infection period, cells were chosen while exposed to 2μg/mL puromycin, and puromycin-resistant cells were gathered and cultivated. Stable cell line construction was finished.

**RNA isolation and qRT-PCR**

The TRIzol reagent (Invitrogen Corporation, Waltham, MA, USA) was used to extract total RNA from tissues or cells in accordance with the manufacturer's instructions. We measured the concentration and purity of the RNA using a NanoDrop 2000 equipment (Thermo Scientific, Waltham, MA, USA). Total RNA was converted to first-strand cDNA using a GoScript Reverse Transcription System (Promega, Madison, WI, USA). qRT-PCR was carried out using the GoTaq qRT-PCR Master Mix Kit (Promega, Madison, WI, USA). For normalization, GAPDH was employed as an endogenous control. Genscript was used to customize all of the primers used in this investigation. **Supplementary Table 1** contains the primer sequences.

**HE staining**
The HE staining method was performed according to standard histological staining techniques. Firstly, tissue sections were subjected to deparaffinization and dehydration, followed by deparaffinization in xylene and dehydration in different concentrations of ethanol. Subsequently, the sections were air-dried in a 70°C oven. Next, the sections were immersed in a staining dish containing hematoxylin dye and stained at room temperature for 10 minutes. Then, the sections were rinsed in tap water until a blue reaction appeared. Afterwards, the sections were transferred to an acidic rinse solution, followed by rinsing in tap water again. Subsequently, the sections were immersed in a staining dish containing eosin dye and stained at room temperature for 5 minutes. Finally, the sections were washed and dehydrated in different concentrations of ethanol, followed by clarification in xylene. The stained sections were then observed and photographed under a microscope.

**Immunofluorescence staining**

CAFs were identified by immunofluorescence labeling, which detected fibroblast activation protein (FAP) and alpha-smooth muscle actin (A-SMA). Sections of tumor tissue were fixed for 15 minutes with 4% paraformaldehyde and then permeabilized for 10 minutes with 0.3% Triton X-100. Following an hour of blocking with 5% bovine serum albumin (BSA), the sections were incubated with primary antibodies against A-SMA (1:200, Abcam) and FAP (1:200, Abcam) for an entire night at 4°C. The next day, sections were treated with the appropriate fluorescently tagged secondary antibodies (Alexa Fluor 488 and 594, Invitrogen) for an hour at room temperature in the dark.DAPI was used to counterstain the nuclei, and a fluorescence microscope was used to take pictures. A-SMA and FAP co-expression was utilized to verify that CAFs were present in the TME.

**WB**

Total cell proteins were extracted using the same lysis solution utilized for the radioimmunoprecipitation experiment. The protein content was determined using the bicinchoninic acid test technique (Biyuntian, Jiangsu, China). Equal quantities of proteins were added to a 10% SDS-polyacrylamide gel electrophoresis gel. Following electrophoresis, the proteins were blotted onto a polyvinylidene fluoride membrane. Primary antibodies were incubated overnight at 4°C after the membrane was blocked in 5% nonfat milk. The secondary antibody was then treated for two hours at 37°C. The following primary antibodies were employed: GDF15 (Abcam, Cambridge, MA, USA, 1:1000), SOD2 (Cell Signaling Technology, Boston, MA, USA, 1:500), GPX1 (Cell Signaling Technology, 1:500), Nrf2 (Cell Signaling Technology, 1:500), GFRAL (Abcam, 1:500), TNF-α (Abcam, 1:1000), EGF (Abcam, 1:1000), IL6 (Abcam, 1:1000), IRF5 (Abcam, 1:1000), Bcl-2 (Abcam, 1:1000), Bax (Abcam, 1:1000), Cleaved caspase3 (Abcam, 1:1000), Akt (Cell Signaling Technology, 1:1000), phospho-Akt (Cell Signaling Technology, 1:1000), ERK1/2 (Cell Signaling Technology, 1:1000), phospho-ERK1/2 (Cell Signaling Technology, 1:1000), PI3K (Cell Signaling Technology, 1:1000), phospho- PI3K (Cell Signaling Technology, 1:1000), CTCF (Abcam, 1:1000), TBP (Proteintech Group, 1:1000), PCLAF (Abcam, ab96510), p-TGFBR2 (Abcam, ab183037), TGFBR2 (Abcam, ab186838) at a 1:5000 dilution, Phospho-STAT3 (Cell Signaling, Ser465/467), STAT3 (Cell Signaling, #5339) at a 1:1000 dilution and β-actin (Cell Signaling Technology,1:5000).

**Co-immunoprecipitation**

After being cleaned with cold PBS, the cells were rinsed with either PBS or RIPA buffer, a cell lysis solution that contains protease inhibitors. After adding enough cell lysis solution, the cells were kept on ice for half an hour to guarantee full cell lysis. After scraping off the cells, the supernatant was collected and placed in centrifuge tubes. After removing the nuclei and cell debris by centrifugation, the supernatant was collected as the cell lysate. Pre-prepared antibodies GDF15 for Flag, IRF5 for His were mixed to allow them to bind. An appropriate amount of the mixture was added to the extracted cell lysate, gently shaken, and incubated overnight at 4°C to allow the antibodies to bind to the target protein. Magnetic bead separator or centrifuge was used to remove nonspecifically bound proteins, and the supernatant was collected. The precipitated magnetic beads were washed with an appropriate amount of washing buffer (such as PBS or TBST) to remove nonspecifically bound proteins. The washing steps were repeated 2-3 times to ensure thorough washing. The magnetic bead precipitate was added to the SDS-PAGE gel, and protein separation was performed. The target protein and its associated proteins were shown to be present and abundant using the WB approach. WB images were analyzed to determine the presence and abundance of the target protein and its interacting proteins.

**Dual-luciferase reporter assay**

A dual-luciferase reporter experiment was used to determine whether CTCF binds to the promoter regions of SOD2, GPX1, and NRF2 in HNSCC cells. The pGL3-basic luciferase reporter vector (Promega, USA) was cloned using the PCR-amplified promoter sequences of SOD2, GPX1, and NRF2. The reporter plasmids and either CTCF overexpression plasmids or control vectors were co-transfected into HNSCC cells using Lipofectamine 3000 (Thermo Fisher Scientific, USA). Co-transfection of the pRL-TK Renilla luciferase plasmid served as an internal check for normalization. The Dual-Luciferase Reporter Assay System (Promega, USA) was used 48 hours later to measure luciferase activity in accordance with the manufacturer's instructions. The relative luciferase activity was calculated by adjusting Firefly luciferase activity to Renilla luciferase activity in order to determine CTCF binding effects.

**Chromatin immunoprecipitation (ChIP) assay**

Following the manufacturer's instructions, the ChIP test was completed using a ChIP assay kit (Beyotime, China). To put it briefly, 200–1000 bp DNA fragments were produced by sonicating HNSCC cells treated with 1% paraformaldehyde. After that, the lysate was immunoprecipitated overnight using either IgG or anti-CTCF antibodies. After removing the cross-linking between DNA and protein for four hours at 65°C, qRT-PCR was used to evaluate the DNA.

**Flow cytometry analysis**

The surface expression of ROS, CD154, and CD95 by HNSCC cells and neutrophils treated with the appropriate conditions was evaluated using flow cytometry analysis.

**Apoptosis assay**

At 4°C, neutrophils were labeled with anti-human Annexin V antibody and PI (BioLegend). Within 15 minutes, flow cytometry was used to assess the cells' apoptosis. For xenograft-derived neutrophils, CD15 and CD16 antibodies were used to identify them and then we assessed cell apoptosis by Annexin V and PI staining.

**ELISA**

Following the manufacturer's instructions, ELISA kits (BioLegend and Abcam) were used to analyze the secretion of GDF15, PCLAF, TGF-β1, TNF-α-EGF, IL-6, sTRAIL, VEGFA, and MCP1 in the serum or culture supernatants. The median concentration value was taken into consideration as the cut-off value to determine low/high GDF15 expression based on ELISA data.

**Animal model construction**

To create cell-derived xenograft (CDX), patient-derived xenograft (PDX), and patient-derived organoid (PDO) models of HNSCC, naked female mice (4-6 weeks old) were used. HNSCC cell lines were obtained for the CDX model, reconstituted in a 1:1 solution of Matrigel (Corning) and PBS at a density of 1×10^7^ cells/mL, and subsequently administered subcutaneously (1×106 cells per location) to the flank regions of the animals. For the PDX model, freshly resected HNSCC tumor tissues from patients were minced into small fragments (approximately 1mm³) and similarly mixed with Matrigel before subcutaneous implantation into the flanks of nude mice. The PDO model was generated by culturing patient-derived HNSCC organoids in a suitable extracellular matrix, followed by subcutaneous injection of the organoid-Matrigel mixture (1×10^6^ organoid cells per site) into the mice. Caliper measurements were used to track tumor development every two weeks, and the formula Volume = (length×width²)/2 was used to determine the tumor's volume. Mice were put down after the tumors had grown to around 2 cm³, and the tumors were removed for additional examination. Every animal procedure was authorized by the appropriate animal care and use committees and carried out in compliance with institutional ethical standards.

**Molecular dynamics simulation**

Small molecules approved by the U.S. Food and Drug Administration (US FDA) were retrieved from the ZINC database[17] as a ligand screening library. All downloaded structures were separated into individual mol2 files using OpenBabel v3.0.0. Both the protein receptor (GDF15, PDB ID: 5VT2) and small molecule ligands were prepared using MGLTools v1.5.6[18], based on AutoDock v4.2.6. Docking was performed with AutoDock Vina v1.1.2 [19, 20] using the catalytic pocket coordinates of GDF15 predicted by Caver[21]. The docking grid was centered at (23.4, 11.9, -12.8) with a cubic box size of 24 × 24 × 24 Å³. For each docking, five conformations were retained with binding energy differences less than 3 kcal/mol, and an exhaustiveness parameter set to 16.

Molecular dynamics simulations were conducted using Desmond v6.6 with the OPLS4 force field[22-24] to construct and simulate GDF15-GFRAL, GDF15-GFRAL-SM-1, and GDF15-GFRAL-SM-2 systems. System preparation was carried out in Maestro. Based on protein annotations from the UniProt database and referencing PDB structure 6Q2J, the systems were embedded in a conventional palmitoyl-oleoyl-phosphatidylcholine (POPC) membrane, solvated with TIP3P water molecules on both sides of the membrane, and neutralized with 0.15 M NaCl. The system underwent 500 ps of energy minimization, followed by 1000 ps each of NVT and NPT equilibration. Subsequently, a 200 ns production simulation was performed with a 2 fs timestep. Binding free energies between small molecules and proteins were further evaluated using the MM/GBSA method. Trajectory analyses including root mean square deviation (RMSD), root mean square fluctuation (RMSF), radius of gyration (Rg), and solvent-accessible surface area (SASA) were visualized using Origin v2021. Protein three-dimensional structures were visualized and analyzed using PyMOL v3.0.0. Free energy landscape (FEL) plots based on RMSD and Rg were generated to investigate protein conformational dynamics.

The dynamic cross-correlation matrix (DCCM) was employed to explore the interaction correlations between GDF15 and GFRAL. DCCM analysis involved dimensionality reduction of the simulation trajectory by aligning atomic coordinates of each frame to a reference structure, obtaining aligned atomic positions per frame. For each Cα atom, the average position across the trajectory was calculated, followed by computation of displacement vectors (x, y, z) relative to this mean for every frame. Pairwise dot products of displacement vectors between Cα atoms were then calculated per frame to generate covariance matrices, which were averaged across frames. Correlation coefficients derived from the covariance matrices yielded the final DCCM, reflecting correlated and anti-correlated motions within the protein complex.

**Animal experiment**

Female BALB/c naked mice aged five and six weeks were acquired from Anhui Medical University's Laboratory Animal Center, kept in a pathogen-free environment, and divided into experimental groups at random. 1×10^6^ HNSCC cells were injected into the tail vein of each animal. BALB/c nude mice were given 100 mg/kg D-luciferin (Xenogen, Hopkinton, MA) two weeks later, and they were then bioluminescently scanned with an IVIS 100 Imaging System (Xenogen). The naked mice were then put to death by euthanasia. In each group, the experiment was conducted three times.

To look at how oxidative stress affects HNSCC, we employed PDX, CDX, and PDO models. Xenografts were subjected to SF-induced oxidative stress and cisplatin treatment. Tumor formation rates in mice were measured, tumor volume and viability of tumor cells were assessed, and in vivo imaging evaluations were conducted.

To explore the mechanism of CAFs-derived GDF15 on tumor growth in xenograft models, FaDu and Cal-27 cells (1×10^7^ cells in 100 μL PBS) treated with rhTNF-α and si-p53 subcutaneously implanted into the ventral region of mice (female NOD/SCID mice), aged 6 weeks (n =6 per group).

In order to investigate the impact of CAF-derived GDF15 on tumor growth in xenograft models, mice (female NOD/SCID mice) aged 6 weeks (n = 6 per group) had their ventral regions subcutaneously implanted with NC and GDF15-Ko FaDu cells (1×10^7^ cells in 100 μL PBS) co-injected with or without the MSCs = 1: 1: 1. The PerkinElmer in vivo imaging system was used to track tumors.

To determine how long could the intratumoral Neutrophils live in mice, FaDu cells (1×10^7^ cells in 100 μL PBS) were subcutaneously implanted into the ventral region of mice (female BALB/c nude mice), aged 5 weeks (n = 20 per group). After 4 weeks, the tumors volumes reached 500mm^3^ in all mice. The mice were further intravenously injected with Neutrophils (1×10^7^ cells in 200 μL PBS) isolated from healthy donors via the tail vein. A total of 20 xenografts were harvested at day 29, 30, 31, and 32, respectively (n = 5 per day), to analyze the apoptosis of intratumoral Neutrophils by flow cytometry.

FaDu cells (1×10^7^ cells in 100 μL PBS) transfected with shControl and rhGDF15 co-injected with or without the MSCs = 1: 1: 1 were implanted into the tail vein of mice (female BALB/c nude mice), aged 5 weeks (n = 10 per group), or GDF15 knockout mice in order to study the role of GDF15 in the crosstalk between CAFs, tumor cells, and neutrophils in vivo using the lung metastasis tumor model. All mice had tumor volumes of 200 mm3 after two weeks. Every group was split into two groups at random, for a total of eight groups (n = 5 each group). The mice were randomly injected with 200μL PBS or Neutrophils (1×10^7^ cells in 200μL PBS) isolated from healthy donors and labeled with Neutrophil-specific NIR fluorescent imaging agent (cFLFLFK-PEG3400-cyanine 7, Kerafast, Virginia, USA) via the tail vein at 2nd week. In vivo human Neutrophils were imaged using an IVIS Spectrum (PerkinElmer) 48 hours after infusion. Images were obtained using Living Image (PerkinElmer) under autoexposure, with the fluorescent filter setting at 745nm for excitation and 800nm for emission. The PerkinElmer in vivo imaging system was used to track tumor progression concurrently with neutrophil imaging. For the purpose of measuring data, all animal studies were blinded.

**References:**

1. Wu F, Fan J, He Y, Xiong A, Yu J, Li Y, et al. Single-cell profiling of tumor heterogeneity and the microenvironment in advanced non-small cell lung cancer. Nat Commun. 2021 2021 May 5;12(1):2540. Available from: http://www.ncbi.nlm.nih.gov/entrez/query.fcgi?cmd=Retrieve&db=pubmed&dopt=Abstract&list_uids=33953163&query_hl=1 doi: 10.1038/s41467-021-22801-0

2. Liu Q, Long Q, Zhao J, Wu W, Lin Z, Sun W, et al. Cold-induced reprogramming of subcutaneous white adipose tissue assessed by single-cell and single-nucleus rna sequencing. Research (Wash D C). 2023 2023;6:182. Available from: http://www.ncbi.nlm.nih.gov/entrez/query.fcgi?cmd=Retrieve&db=pubmed&dopt=Abstract&list_uids=37398933&query_hl=1 doi: 10.34133/research.0182

3. Zhou Y, Yang D, Yang Q, Lv X, Huang W, Zhou Z, et al. Single-cell rna landscape of intratumoral heterogeneity and immunosuppressive microenvironment in advanced osteosarcoma. Nat Commun. 2020 2020 Dec 10;11(1):6322. Available from: http://www.ncbi.nlm.nih.gov/entrez/query.fcgi?cmd=Retrieve&db=pubmed&dopt=Abstract&list_uids=33303760&query_hl=1 doi: 10.1038/s41467-020-20059-6

4. Korsunsky I, Millard N, Fan J, Slowikowski K, Zhang F, Wei K, et al. Fast, sensitive and accurate integration of single-cell data with harmony. Nat Methods. 2019 2019 Dec;16(12):1289-96. Available from: http://www.ncbi.nlm.nih.gov/entrez/query.fcgi?cmd=Retrieve&db=pubmed&dopt=Abstract&list_uids=31740819&query_hl=1 doi: 10.1038/s41592-019-0619-0

5. Becht E, McInnes L, Healy J, Dutertre CA, Kwok I, Ng LG, et al. Dimensionality reduction for visualizing single-cell data using umap. Nat Biotechnol. 2018 2018 Dec 3. Available from: http://www.ncbi.nlm.nih.gov/entrez/query.fcgi?cmd=Retrieve&db=pubmed&dopt=Abstract&list_uids=30531897&query_hl=1 doi: 10.1038/nbt.4314

6. Gene ontology consortium: going forward. Nucleic Acids Res. 2015 2015 Jan;43(Database issue):D1049-56. Available from: http://www.ncbi.nlm.nih.gov/entrez/query.fcgi?cmd=Retrieve&db=pubmed&dopt=Abstract&list_uids=25428369&query_hl=1 doi: 10.1093/nar/gku1179

7. Subramanian A, Tamayo P, Mootha VK, Mukherjee S, Ebert BL, Gillette MA, et al. Gene set enrichment analysis: a knowledge-based approach for interpreting genome-wide expression profiles. Proc Natl Acad Sci U S A. 2005 2005 Oct 25;102(43):15545-50. Available from: http://www.ncbi.nlm.nih.gov/entrez/query.fcgi?cmd=Retrieve&db=pubmed&dopt=Abstract&list_uids=16199517&query_hl=1 doi: 10.1073/pnas.0506580102

8. Gao J, Wu Z, Zhao M, Zhang R, Li M, Sun D, et al. Allosteric inhibition reveals shp2-mediated tumor immunosuppression in colon cancer by single-cell transcriptomics. Acta Pharm Sin B. 2022 2022 Jan;12(1):149-66. Available from: http://www.ncbi.nlm.nih.gov/entrez/query.fcgi?cmd=Retrieve&db=pubmed&dopt=Abstract&list_uids=35127377&query_hl=1 doi: 10.1016/j.apsb.2021.08.006

9. Qiu X, Hill A, Packer J, Lin D, Ma YA, Trapnell C. Single-cell mrna quantification and differential analysis with census. Nat Methods. 2017 2017 Mar;14(3):309-15. Available from: http://www.ncbi.nlm.nih.gov/entrez/query.fcgi?cmd=Retrieve&db=pubmed&dopt=Abstract&list_uids=28114287&query_hl=1 doi: 10.1038/nmeth.4150

10. Qiu X, Mao Q, Tang Y, Wang L, Chawla R, Pliner HA, et al. Reversed graph embedding resolves complex single-cell trajectories. Nat Methods. 2017 2017 Oct;14(10):979-82. Available from: http://www.ncbi.nlm.nih.gov/entrez/query.fcgi?cmd=Retrieve&db=pubmed&dopt=Abstract&list_uids=28825705&query_hl=1 doi: 10.1038/nmeth.4402

11. Van den Berge K, Roux DBH, Street K, Saelens W, Cannoodt R, Saeys Y, et al. Trajectory-based differential expression analysis for single-cell sequencing data. Nat Commun. 2020 2020 Mar 5;11(1):1201. Available from: http://www.ncbi.nlm.nih.gov/entrez/query.fcgi?cmd=Retrieve&db=pubmed&dopt=Abstract&list_uids=32139671&query_hl=1 doi: 10.1038/s41467-020-14766-3

12. Jin S, Guerrero-Juarez CF, Zhang L, Chang I, Ramos R, Kuan CH, et al. Inference and analysis of cell-cell communication using cellchat. Nat Commun. 2021 2021 Feb 17;12(1):1088. Available from: http://www.ncbi.nlm.nih.gov/entrez/query.fcgi?cmd=Retrieve&db=pubmed&dopt=Abstract&list_uids=33597522&query_hl=1 doi: 10.1038/s41467-021-21246-9

13. Friedman J, Hastie T, Tibshirani R. Regularization paths for generalized linear models via coordinate descent. J Stat Softw. 2010 2010;33(1):1-22. Available from: http://www.ncbi.nlm.nih.gov/entrez/query.fcgi?cmd=Retrieve&db=pubmed&dopt=Abstract&list_uids=20808728&query_hl=1

14. Aste N, Pau M, Cordaro CI, Biggio P. Double-blind study with fenticonazole or bifonazole lotions in pityriasis versicolor. Int J Clin Pharmacol Res. 1988 1988;8(4):271-73. Available from: http://www.ncbi.nlm.nih.gov/entrez/query.fcgi?cmd=Retrieve&db=pubmed&dopt=Abstract&list_uids=3182117&query_hl=1

15. Chen H, Luo J, Guo J. Development and validation of a five-immune gene prognostic risk model in colon cancer. Bmc Cancer. 2020 2020 May 6;20(1):395. Available from: http://www.ncbi.nlm.nih.gov/entrez/query.fcgi?cmd=Retrieve&db=pubmed&dopt=Abstract&list_uids=32375704&query_hl=1 doi: 10.1186/s12885-020-06799-0

16. Lee JH, Jung S, Park WS, Choe EK, Kim E, Shin R, et al. Prognostic nomogram of hypoxia-related genes predicting overall survival of colorectal cancer-analysis of tcga database. Sci Rep. 2019 2019 Feb 12;9(1):1803. Available from: http://www.ncbi.nlm.nih.gov/entrez/query.fcgi?cmd=Retrieve&db=pubmed&dopt=Abstract&list_uids=30755640&query_hl=1 doi: 10.1038/s41598-018-38116-y

17. Sterling T, Irwin JJ. Zinc 15--ligand discovery for everyone. J Chem Inf Model. 2015 2015 Nov 23;55(11):2324-37. Available from: http://www.ncbi.nlm.nih.gov/entrez/query.fcgi?cmd=Retrieve&db=pubmed&dopt=Abstract&list_uids=26479676&query_hl=1 doi: 10.1021/acs.jcim.5b00559

18. Morris GM, Huey R, Lindstrom W, Sanner MF, Belew RK, Goodsell DS, et al. Autodock4 and autodocktools4: automated docking with selective receptor flexibility. J Comput Chem. 2009 2009 Dec;30(16):2785-91. Available from: http://www.ncbi.nlm.nih.gov/entrez/query.fcgi?cmd=Retrieve&db=pubmed&dopt=Abstract&list_uids=19399780&query_hl=1 doi: 10.1002/jcc.21256

19. Eberhardt J, Santos-Martins D, Tillack AF, Forli S. Autodock vina 1.2.0: new docking methods, expanded force field, and python bindings. J Chem Inf Model. 2021 2021 Aug 23;61(8):3891-98. Available from: http://www.ncbi.nlm.nih.gov/entrez/query.fcgi?cmd=Retrieve&db=pubmed&dopt=Abstract&list_uids=34278794&query_hl=1 doi: 10.1021/acs.jcim.1c00203

20. Trott O, Olson AJ. Autodock vina: improving the speed and accuracy of docking with a new scoring function, efficient optimization, and multithreading. J Comput Chem. 2010 2010 Jan 30;31(2):455-61. Available from: http://www.ncbi.nlm.nih.gov/entrez/query.fcgi?cmd=Retrieve&db=pubmed&dopt=Abstract&list_uids=19499576&query_hl=1 doi: 10.1002/jcc.21334

21. Jurcik A, Bednar D, Byska J, Marques SM, Furmanova K, Daniel L, et al. Caver analyst 2.0: analysis and visualization of channels and tunnels in protein structures and molecular dynamics trajectories. Bioinformatics. 2018 2018 Oct 15;34(20):3586-88. Available from: http://www.ncbi.nlm.nih.gov/entrez/query.fcgi?cmd=Retrieve&db=pubmed&dopt=Abstract&list_uids=29741570&query_hl=1 doi: 10.1093/bioinformatics/bty386

22. Lu C, Wu C, Ghoreishi D, Chen W, Wang L, Damm W, et al. Opls4: improving force field accuracy on challenging regimes of chemical space. J Chem Theory Comput. 2021 2021 Jul 13;17(7):4291-300. Available from: http://www.ncbi.nlm.nih.gov/entrez/query.fcgi?cmd=Retrieve&db=pubmed&dopt=Abstract&list_uids=34096718&query_hl=1 doi: 10.1021/acs.jctc.1c00302

23. Shivakumar D, Williams J, Wu Y, Damm W, Shelley J, Sherman W. Prediction of absolute solvation free energies using molecular dynamics free energy perturbation and the opls force field. J Chem Theory Comput. 2010 2010 May 11;6(5):1509-19. Available from: http://www.ncbi.nlm.nih.gov/entrez/query.fcgi?cmd=Retrieve&db=pubmed&dopt=Abstract&list_uids=26615687&query_hl=1 doi: 10.1021/ct900587b

24. Jorgensen WL, Tirado-Rives J. The opls [optimized potentials for liquid simulations] potential functions for proteins, energy minimizations for crystals of cyclic peptides and crambin. J Am Chem Soc. 1988 1988 Mar 1;110(6):1657-66. Available from: http://www.ncbi.nlm.nih.gov/entrez/query.fcgi?cmd=Retrieve&db=pubmed&dopt=Abstract&list_uids=27557051&query_hl=1 doi: 10.1021/ja00214a001

**2. Supplementary Figures**

**
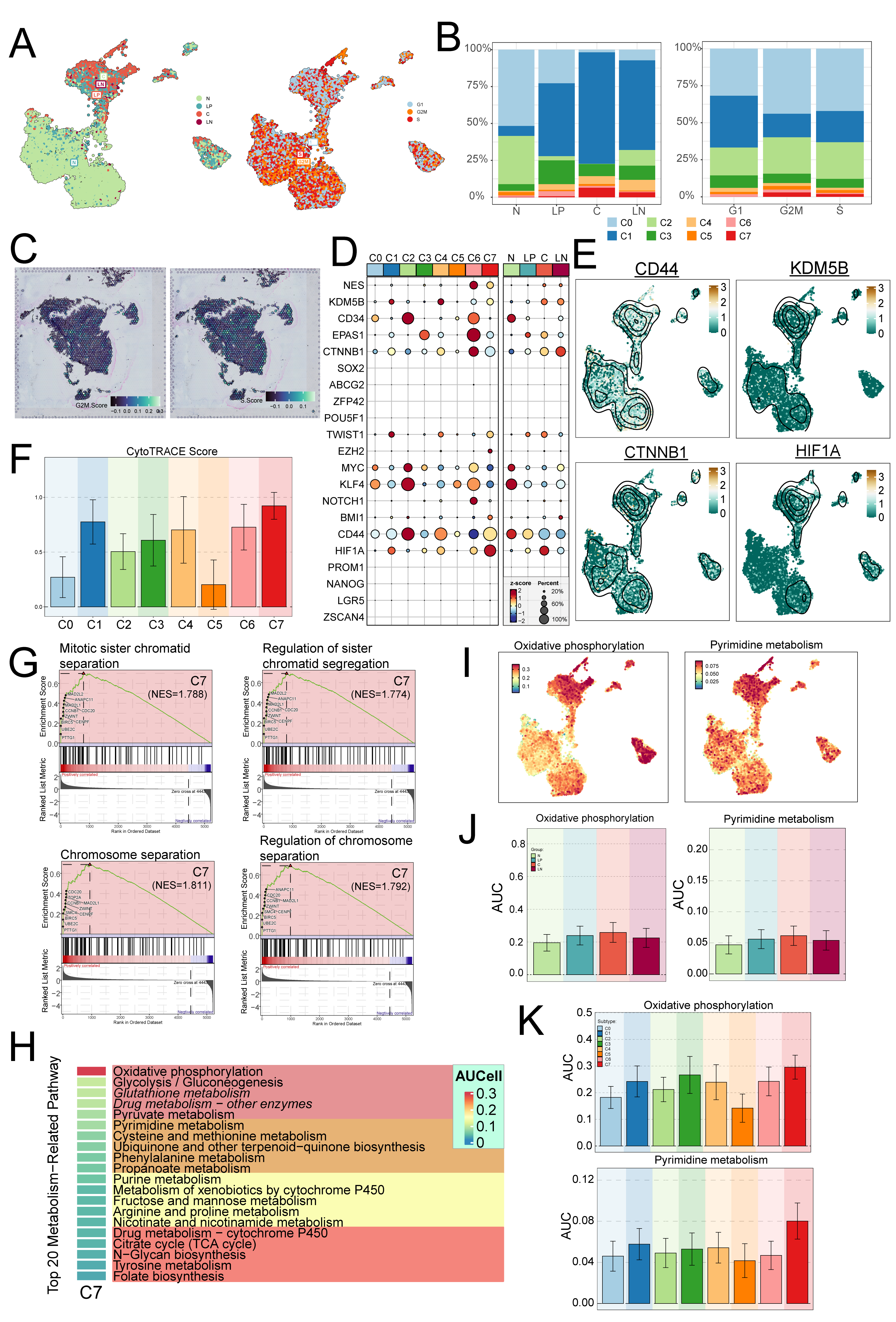
**

**Supplementary Figure 1. C7 PCLAF^+^ fibroblasts had high proliferative properties.**  (A)Utilizing UMAP plots visualization to illustrate the distribution of fibroblasts across different tissue types and cell cycle phases. Left: Group (normal tissue (NL), precancerous leukoplakia (LP), primary carcinoma (C), lymph node metastases (LN)), Right: phase (G1, G2/M, S). (B)visualization of bar graphs showed the percentage of fibroblast subtypes in different tissue types and cell cycle stages. (C) TransferData illustrated the spatial transcriptomic landscape of the G2/M score and S score in HNSCC. (D) The bubble plot demonstrated the differential expression of stemness genes in fibroblast subtypes and tissue sources. Colors are derived from normalized data, and bubble sizes represent gene expression scores. (E) UMAP plots (shown with outlines) displayed the distribution of stemness genes (CD44, CTNNB1, KDM5B, and HIF1A) in HNSCC fibroblast subtypes. (F) CytoTRACE analysis depicted CytoTRACE scores for different fibrobloast clusters, where higher scores indicate cells with greater stemness and differentiation potential. (G) The GSEA enrichment analysis results of top 4were presented in the C7 subtype. (H) Heatmap showed the regulatory differences of the top20 metabolism-related pathway in the C7 PCLAF^+^ fibroblasts. (I-K) The UMAP plots and bar graphs showed the enrichment differences of different metabolism pathway in different tissue sources and fibroblast subtypes.


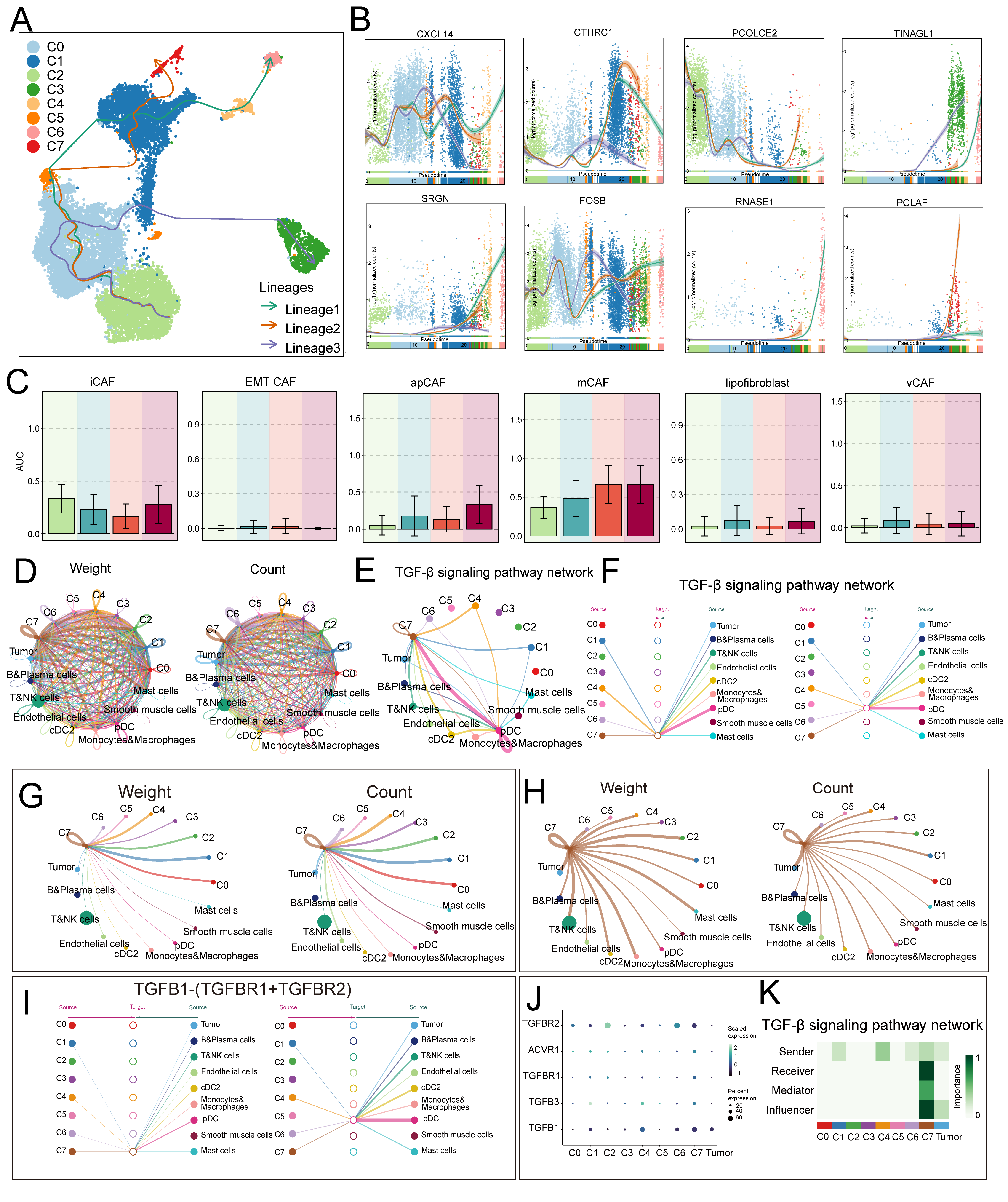


**Supplementary Figure 2. Biological pathway characterization of C7 PCLAF^+^ fibroblasts.** (A) Slingshot inferred three differentiation trajectories of fibroblast subtypes. The solid line indicates the differentiation trajectory, and the arrow indicated the direction of differentiation (from naive to mature). (B) Scatterplots showed the variation and distribution of marker genes in 8 fibroblast subtypes in 3 differentiation trajectories, arranged in pseudo-chronological order. Curves represented gene expression profiles fitted based on normalized data, and scatter colors indicated different fibroblast clusters. Color-coded by cell types. (C) The bar graphs illustrated the AUCell scores of apCAF, vCAF, mCAF, lipofibroblast, iCAF and EMT-CAF related genes in different tissue sources. (D) Circular plots displayed the weight (left) and count (right) of receptor-ligand interactions within the entire cell population. (E) Circular plot screened the receiver of Transforming Growth Factor – β (TGF-β) signaling pathway as fibroblasts. (F) Hierarchical plot depicted the interactions among cells in the TGF-β signaling pathway. (G-H) Circular plots illustrated the quantity of cell interactions where fibroblasts were the target and source and the strength of these interactions. (I) Hierarchical plot depicted the interactions among cells in the TGFB1(TGFBR1+TGFBR2) receptor ligand pair. (J) The interactions between cells in the TGF-β signaling pathway were shown in a bubble plot. (K) The proportional significance of each type of cell as a transmitter, receiver, mediator, and influencer was depicted in a heatmap based on four network centrality measurements of the TGF-β signal.


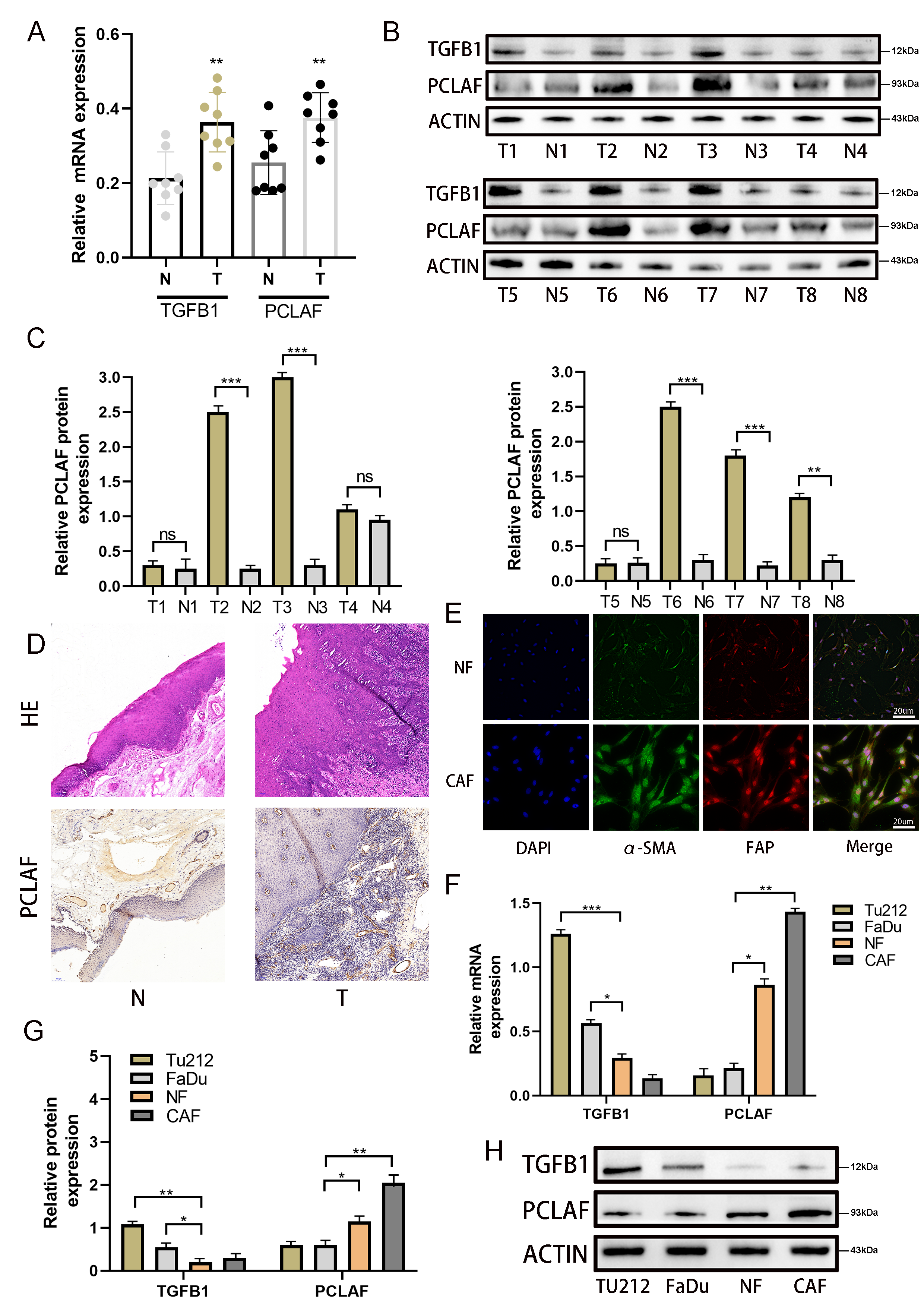
**Supplementary Figure 3. Overexpression of PCLAF and TGFB1 in HNSCC.** (A) Relative mRNA expression of TGFB1 and PCLAF in paired HNSCC tumor (T) and adjacent normal (N) tissues (n = 10 pairs). (B) WB analysis of TGFB1 and PCLAF protein levels in representative tumor and normal samples from 8 patients. (C) Quantification of PCLAF protein expression from (B), shown separately for T1–T4 (left) and T5–T8 (right) sample groups. (D) Representative images of HE staining (top) and PCLAF immunohistochemistry (bottom) in adjacent normal (N) and tumor (T) tissues. (E) Immunofluorescence staining of normal fibroblasts (NF) and CAFs for α-SMA and FAP. DAPI indicates nuclear staining. Scale bar = 20 μm. (F) qRT-PCR analysis of TGFB1 and PCLAF mRNA levels in Tu212, FaDu, NF, and CAFs cells. (G) Quantification of TGFB1 and PCLAF protein expression from (H). (H) WB analysis of TGFB1 and PCLAF protein levels in Tu212, FaDu, NF, and CAFs. Data are presented as mean ± SD. **p < 0.05, **p < 0.01, ***p < 0.001* by paired or unpaired t-test as appropriate.


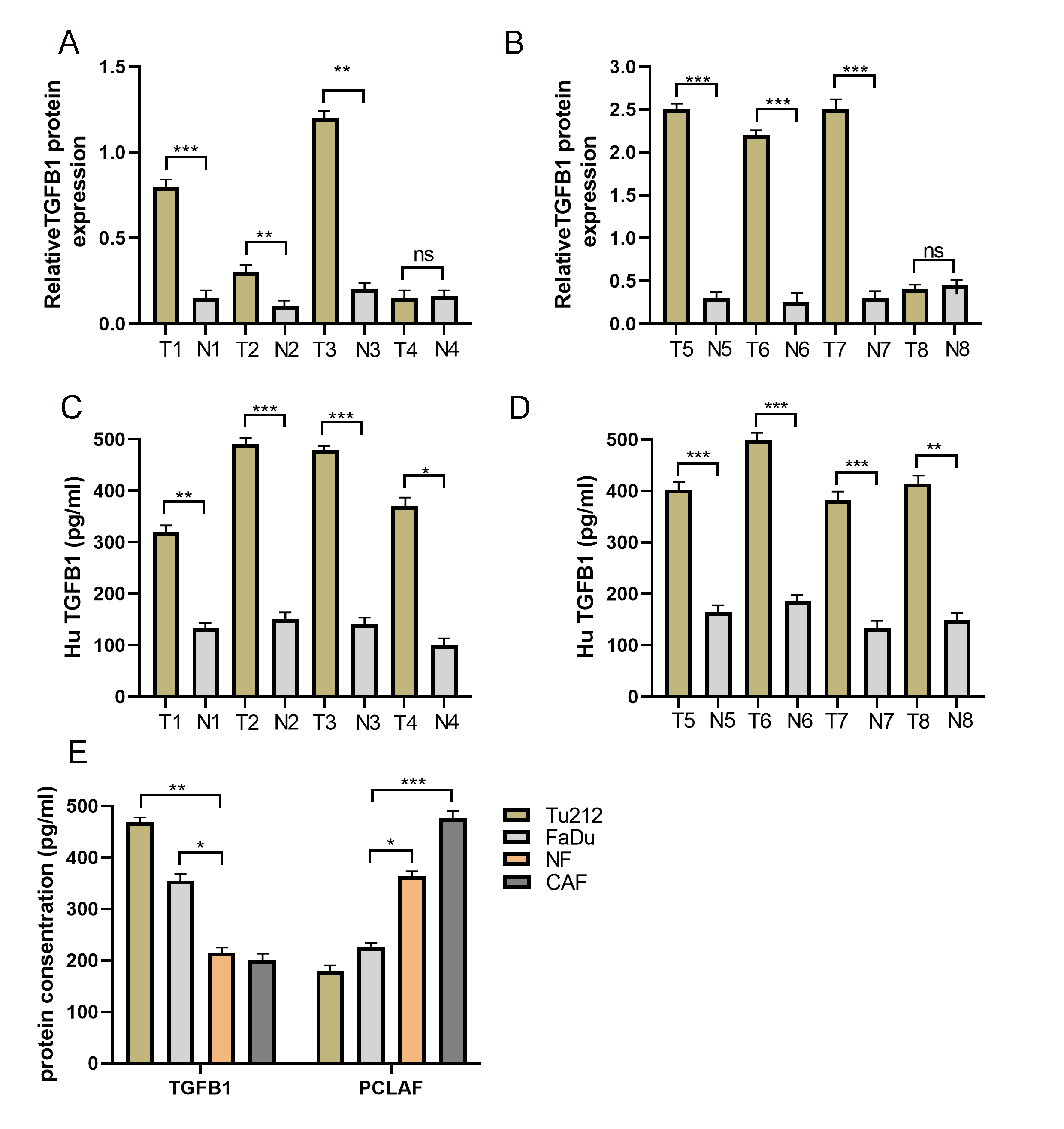


**Supplementary Figure 4.** **Quantitative analysis.** (A, B) Quantitative analysis of WB confirmed increased expression of TGFB1 in HNSCC tissues compared to normal tissues. (C, D) Quantitative analysis of ELISA confirming increased expression of TGFB1 in HNSCC tissues compared to normal tissues. (E) ELISA quantification of TGFBR1 and PCLAF in Different HNSCC Cell Lines and CAFs.

**
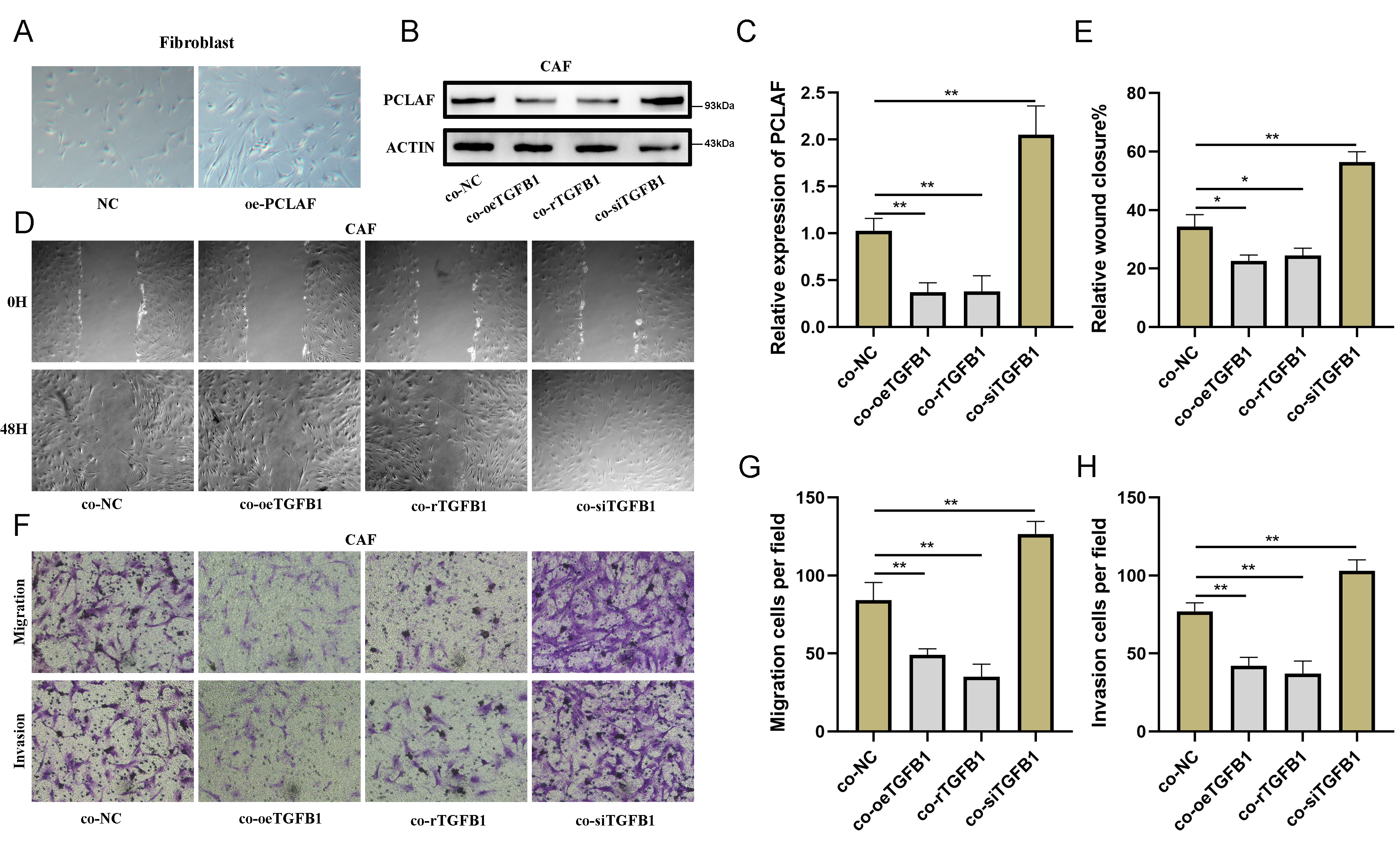
**

**Supplementary Figure 5. Co-culture system demonstrating the regulatory impact of TGFB1 and PCLAF on fibroblast malignant behavior.** (A) Representative micrographs illustrated enhanced invasiveness following PCLAF overexpression in normal fibroblasts. (B, D, F) After CAFs were co-cultured with TGFB1-overexpressing Tu212 cells (co-oeTGFB1), TGFB1-knockdown Tu212 cells (co-siTGFB1), and medium supplemented with recombinant TGFB1 (co-rTGFB1), the protein expression of PCLAF and the malignant behavior of CAFs were altered. (C) Relative quantification of protein expression by WB. (E) Relative quantification of scratch assay results. (G, H) Relative quantification of transwell assay results.


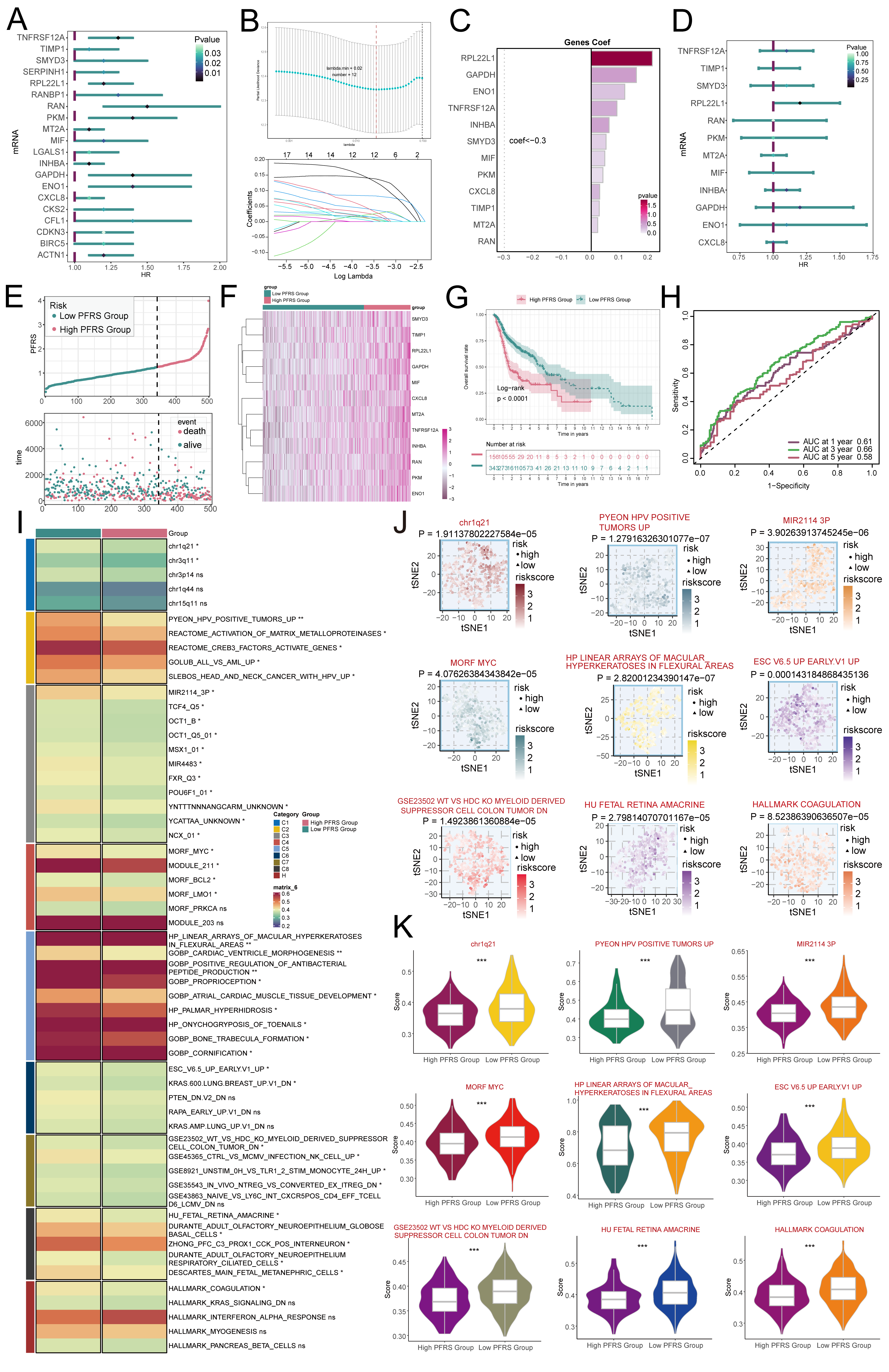


**Supplementary Figure 6. Prognostic analysis of the High PFRS Group and the Low PFRS Group.** High PFRS group: high C7 PCLAF^+^ fibroblasts risk score group; low PFRS group: low C7 PCLAF^+^ fibroblasts risk score group. (A) Forest plot showed the results of univariate Cox regression analysis of genes constituting the risk score, where 20 genes with P < 0.05 were displayed. Null line HR = 1, HR < 1: protective factor, HR > 1: risk factor. (B) Twelve genes associated with prognosis were identified by lasso regression analysis (Lambda.min=0.02). (C) Bar graph demonstrated the Genes Coef values for the constructed model genes. (D) Multivariate Cox regression analysis results of 12 risk score genes. (E) A curve graph illustrated the hazard scores for both the high PFRS group and the low PFRS group (above). The scatter plot displayed the survival and death events over time for both the high PFRS group and the low PFRS group (below). (F) A heatmap depicted the differential expression of modeling genes between the high PFRS group and the low PFRS group. (G) The survival curve illustrated the survival differences among different risk score groups. (H) Calculated theAUC for predicting outcomes at the 1st, 3rd, and 5th years in the queue. (I) GSVA delineated the biological attributes of the two PFRS groups. *ns no significance, *p < 0.05, **p < 0.01*. (J)The t-SNE plots illustrated the distribution of risk scores, obtained from the top-ranked GSVA enrichment entries for all differential gene sets, within the high PFRS group and low PFRS group. (K) The violin plots compared the score differences of the aforementioned enrichment entries between the high PFRS group and low PFRS group. ****p < 0.001*.


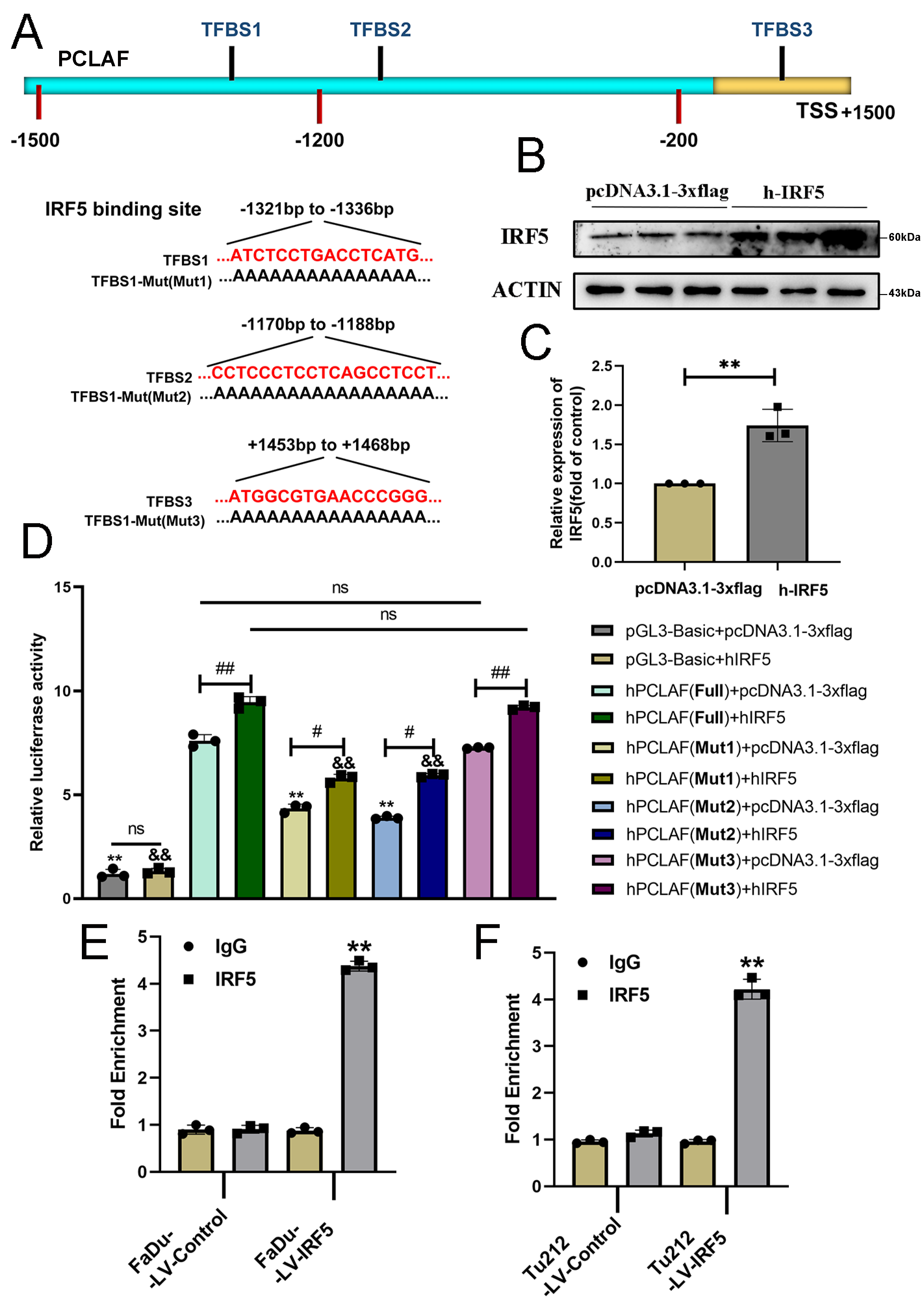


**Supplementary Figure 7. Dual-luciferase reporter assay and CHIP validation demonstrated transcription factor IRF5 regulating PCLAF.** (A) Schematic representation of the predicted IRF5 binding sites (TFBS1–3) in the PCLAF promoter region. Mutated sequences (Mut1–3) are shown in red. (B) WB confirmed IRF5 overexpression in 3×Flag-tagged hIRF5-transfected cells. (C) QRT-PCR quantification of IRF5 mRNA expression in control and hIRF5-overexpressing cells. (D) Dual-luciferase reporter assay showed relative PCLAF promoter activity in cells transfected with full-length (Full) or TFBS-mutated (Mut1, Mut2, Mut3) promoter constructs, with or without IRF5 overexpression. (E–F) ChIP-qPCR analysis of IRF5 binding to the PCLAF promoter in FaDu (E) and Tu212 (F) cells using anti-IRF5 or control IgG antibodies. Results were presented as fold enrichment relative to IgG. Data represented mean ± SD from three independent experiments. ***p < 0.01, ##p < 0.01* vs. control group, *&&p < 0.01* vs. hPCLAF (FULL)+IRF5 group, ns: not significant.


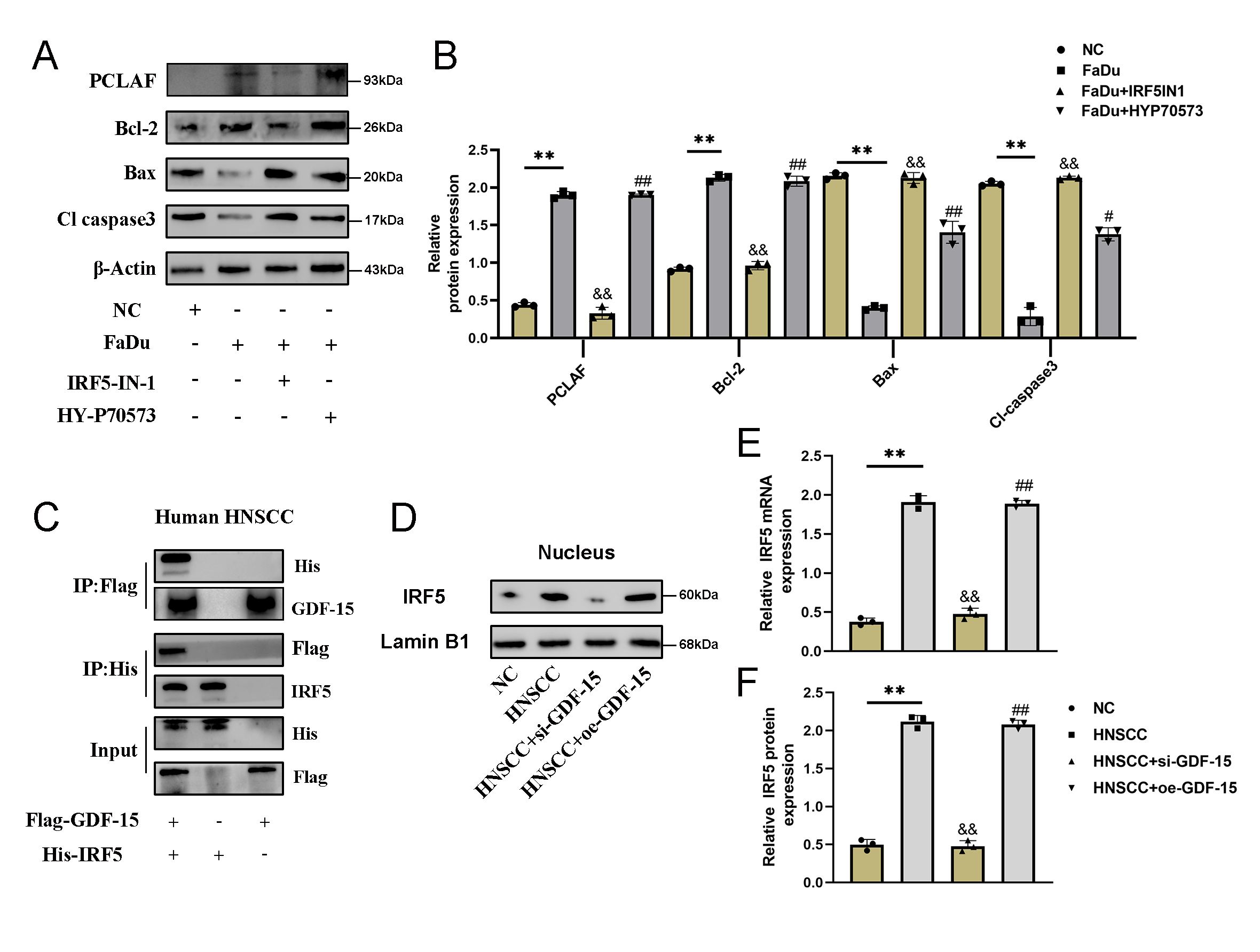


**Supplementary Figure 8. Validation of the reciprocal interaction between GDF15 and IRF5 in regulating PCLAF in HNSCC.** (A) WB analysis of PCLAF, Bcl-2, Bax, and cleaved caspase-3 in FaDu cells treated with IRF5 inhibitor (IRF5-IN-1) or recombinant IRF5 protein (HY-P70573). (B) Quantification of protein expression from (E), normalized to β-actin. (C) Co-IP of Flag-tagged GDF15 and His-tagged IRF5 in human HNSCC cells. Whole cell lysates (input) and IP fractions were immunoblotted with the indicated antibodies. (D) WB of nuclear IRF5 expression in HNSCC tissues or cells transfected with GDF15 overexpression vector (oe-GDF15) or GDF15 siRNA (si-GDF15). Lamin B1 served as the nuclear loading control. (E, F) qRT-PCR (E) and WB (F) quantification of IRF5 expression in control, HNSCC, GDF15-knockdown, and GDF15-overexpressing cells. Data were shown as mean ± SD from three independent experiments. **p < 0.05, **p < 0.01, ***p < 0.001*; *&&p < 0.01* vs. FaDu; *##p < 0.01* vs. IRF5-IN-1 or HYP70573 group.

**
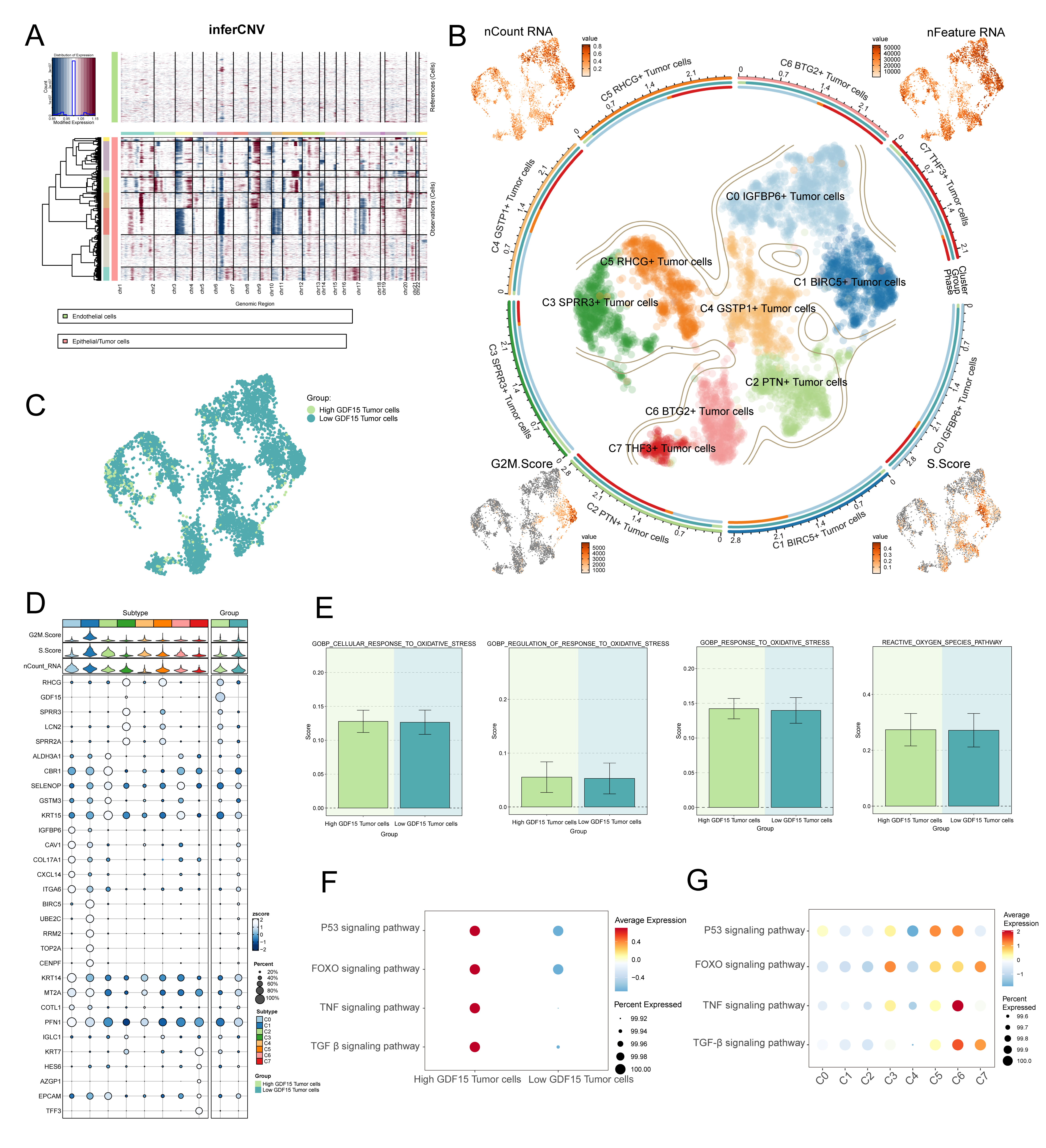
**

**Supplementary Figure 9. Visualization of tumor cells subtypes in HNSCC.** (A) Unsupervised clustering of CNVs to identify cancer cells from epithelia cells. Epithelial cells and endothelial cells were in the y-axis and chromosomal regions in the x-axis. (B) The circular plot visualized the clustering distribution of tumor cells within HNSCC, with tumor cells colored based on their respective categories. Three circular orbits (from outer to inner) sequentially represent cell types , cell source groups and cell cycle stage, and were stained according to the cell categories. Starting from the upper left corner and proceeding clockwise, the four illustrations displayed the nCount-RNA and nFeature-RNA for all cells, along with the scores for the S phase and G2/M phase of the cells. (C) UMAP plot showed that tumor cells were divided into high GDF15 tumor cells and low GDF15 tumor cells according to the expression level of GDF15 gene. (D) The bubble plot presented the expression of differentially expressed genes in 8 cell types and across various GDF groups. The color of the bubbles indicated their expression levels, while the size of the bubbles represented the percentage of their expression levels. (E) Bar graphs showed the scores of the cellular response to oxidative stress, regulation of response to oxidative stress, response to oxidative stress, and the restive oxygen species pathway in different GDF15 groups. (F, G) The bubble plots respectively showed the differences in the regulation of the GDF15 downstream pathway by different GDF15 groups and different tumor cell subtypes.

**Supplementary Figure 10. GDF15 promotes TNF-α–dependent neutrophil recruitment, polarization, and tumor-promoting activity in HNSCC.** (A) Nuclear translocation of CTCF was significantly enhanced by treatment with rhTNF-α and rhGDF15 in HNSCC cells, but reduced upon blocking TNF-α. (B) Relative mRNA expression levels of inflammatory cytokines, chemokines, and immunomodulatory genes were assessed by qRT-PCR in HNSCC cells treated with vehicle control (Ctrl), rhTNF-α, or rhGDF15. rhGDF15 treatment significantly upregulated MCP1, CCL5, VEGFA, VEGFB, TGFB1, IL6, IL8, IL10, and CSF3, whereas expression of MPO, ELA2, ARG1, and ICAM1 remained unchanged. rhTNF-α induced moderate increases in VEGFA, VEGFB, and TGFB1. (C) CD15^+^ and CD16^+^ neutrophils from healthy donors were identified, with over 50% surviving in vitro for 24 hours. (D, E) RhTNF-α enhanced neutrophil recruitment, an effect reversed by blocking TNF-α. (F) Experimental design. (G) Flow cytometry showing neutrophil activation marker expression (CD154 and CD95) following exposure to rhGDF15, CAF-conditioned media (CAF-sup. and CAF-coc.), or anti–TNF-α antibody. (H) WB analysis showed ERK1/2 phosphorylation in FaDu and Cal-27 cells treated with anti-EGF or anti-TNF-α. (I) ELISA quantitative analysis of changes in neutrophil-related factors secreted by tumor cells after rhGDF15, CAF-conditioned medium, or TNF-α blockade. (J) In in vivo experiments, mice injected with shCtrl-FaDu, rhGDF15, and MSCs simultaneously showed faster tumor progression compared with the control group. (K) Neutrophil infusion promoted lung metastasis in shCtrl-FaDu cells injected with MSCs.


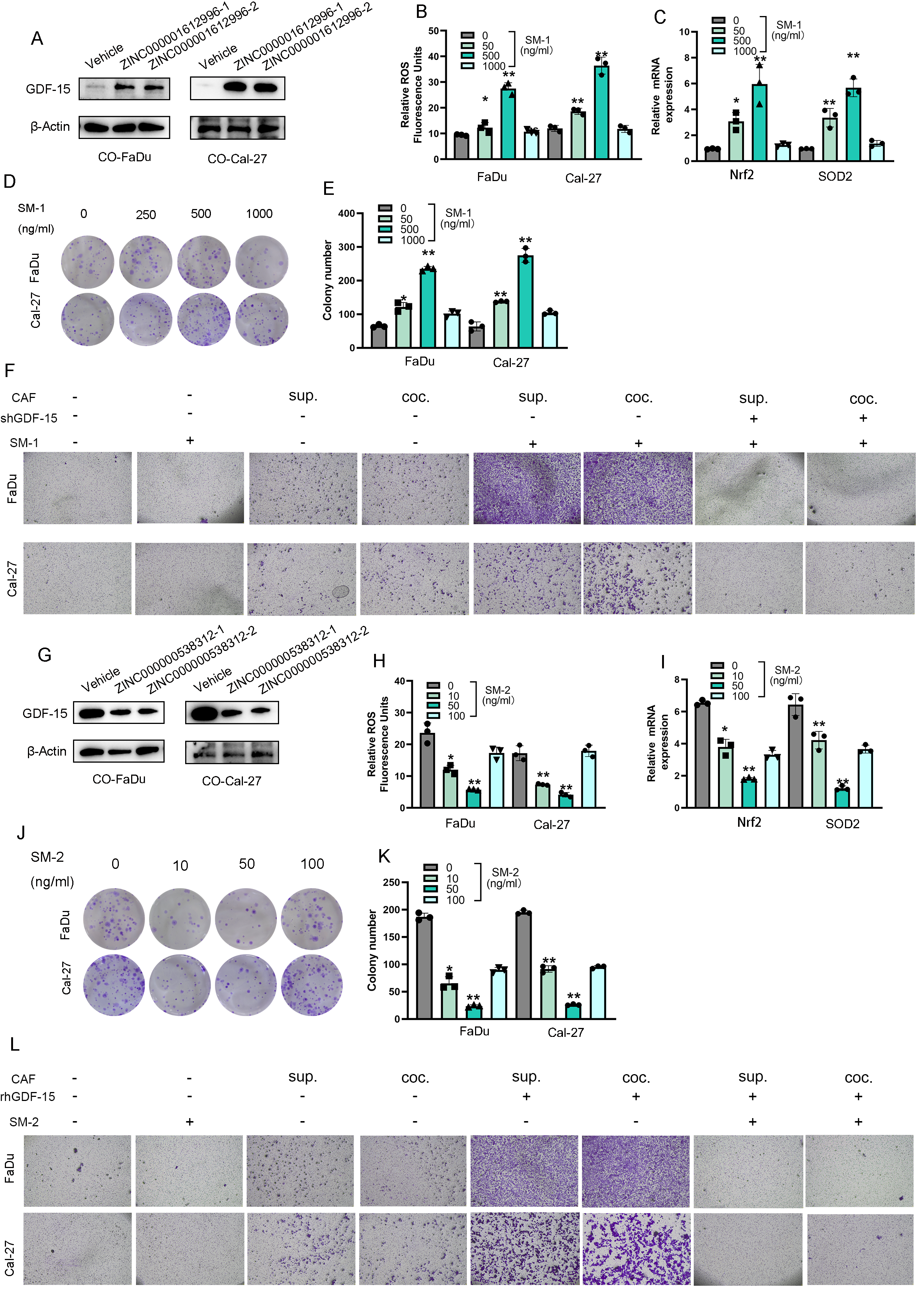


**Supplementary Figure 11. Validation of small-molecule screening.** (A) WB analysis showed that SM-1 increases GDF15 expression in cocultured (CO) FaDu and Cal-27 cells. (B) ROS fluorescence quantification in FaDu and Cal-27 cells treated with increasing concentrations of SM-1. (C) qRT-PCR analysis of Nrf2 and SOD2 mRNA levels following SM-1 treatment. (D) Colony formation assays showed enhanced clonogenicity in SM-1–treated FaDu and Cal-27 cells. (E) Quantification of colony numbers from (D). (F) Transwell assays showed SM-1–induced migration in CAF supernatant (sup.) and coculture (coc.) conditions, which is reduced upon GDF15 knockdown in CAFs. (G) WB analysis showed that SM-2 reduces GDF15 expression in cocultured FaDu and Cal-27 cells. (H) ROS quantification in response to SM-2 treatment. (I) qRT-PCR analysis of Nrf2 and SOD2 mRNA after SM-2 treatment. (J) Colony formation assays showed suppression of proliferation by SM-2. (K) Quantification of colonies from (J). (L) Transwell assays showed that SM-2 suppresses CAFs/rhGDF15-induced migration. Data represented mean ± SD from three independent experiments. **p < 0.05, **p < 0.01* by one-way ANOVA or unpaired t-test as appropriate.

**3.Supplementary Table 1**: Oligonucleotides used in research.

| **Oligonucleotides** | **Nucleotide sequence (5'-3')** |
| --- | --- |
| **siRNA** |  |
| Scramble control | GCUUCGCGCCGUAGUCUUA |
| Si-GDF15-1 | CCGGATACTCACGCCAGAAGT |
| Si-GDF15-2 | CCCTCAGAGTTGCACTCCGAA |
|  |  |
| **Primer** |  |
| GAPDH | GGCCTCCAAGGAGTAAGACC (forward) |
|  | AGGGGAGATTCAGTGTGGTG (reverse) |
| GDF15 | GCAAGAACTCAGGACGGTGA (forward) |
|  | TGGAGTCTTCGGAGTGCAAC (reverse) |
|  |  |
