# Supplementary figures and images for: Cancer-Associated Fibroblast-Derived GDF15 Induces Oxidative Stress and Neutrophil Infiltration in Head and Neck Squamous Cell Carcinoma through the PI3K/AKT/STAT3 Axis Cascade
Source: Research (Wash D C). 2025 Sep 30;8:0901. doi: 10.34133/research.0901 (PMC12480759; doi:10.34133/research.0901)

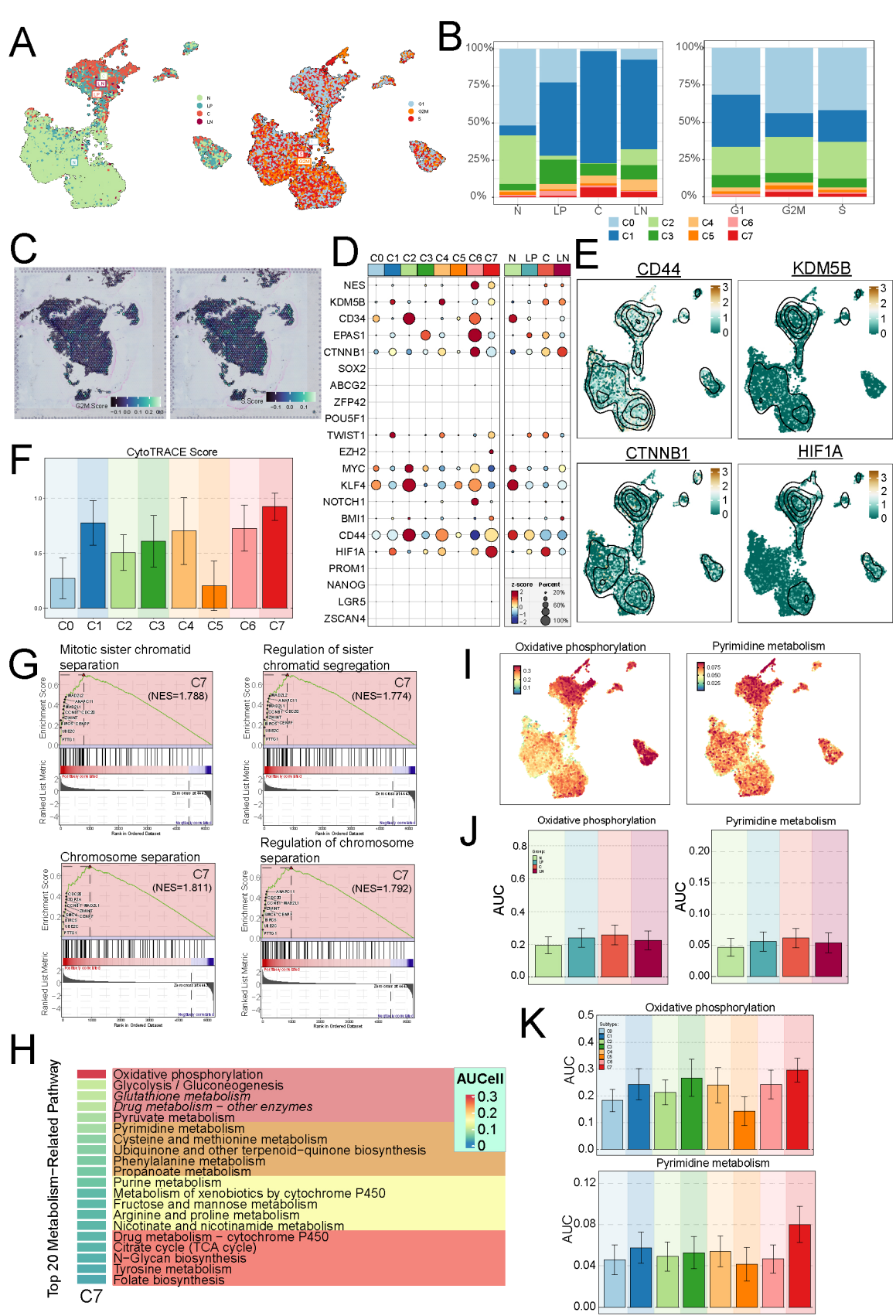

Supplement: Supplementary 1 — Materials and Methods Figs. S1 to S11 Table S1 [file research.0901.f1.zip › Supplementary Figure 1.pdf]

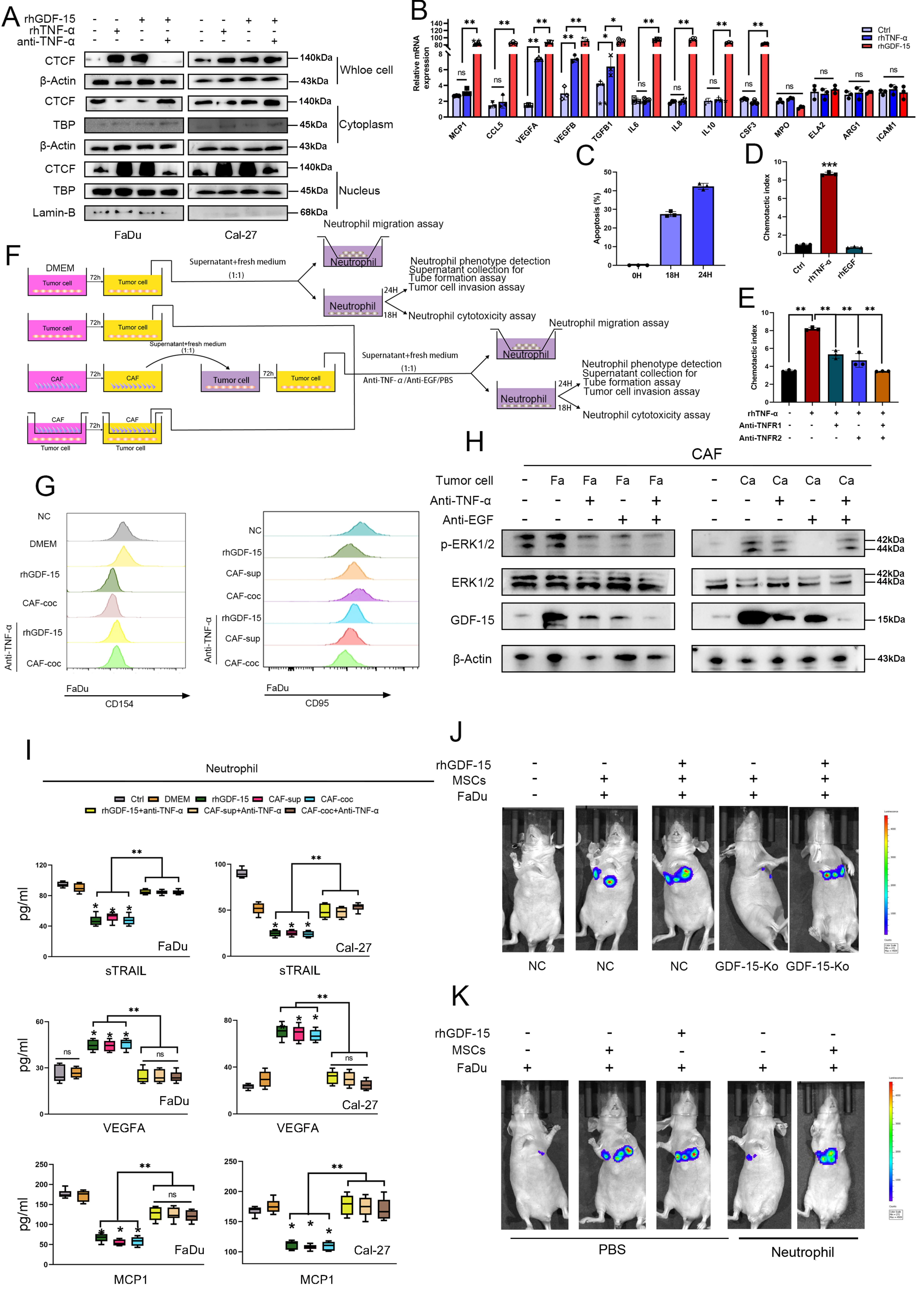

Supplement: Supplementary 1 — Materials and Methods Figs. S1 to S11 Table S1 [file research.0901.f1.zip › Supplementary Figure 10.pdf]

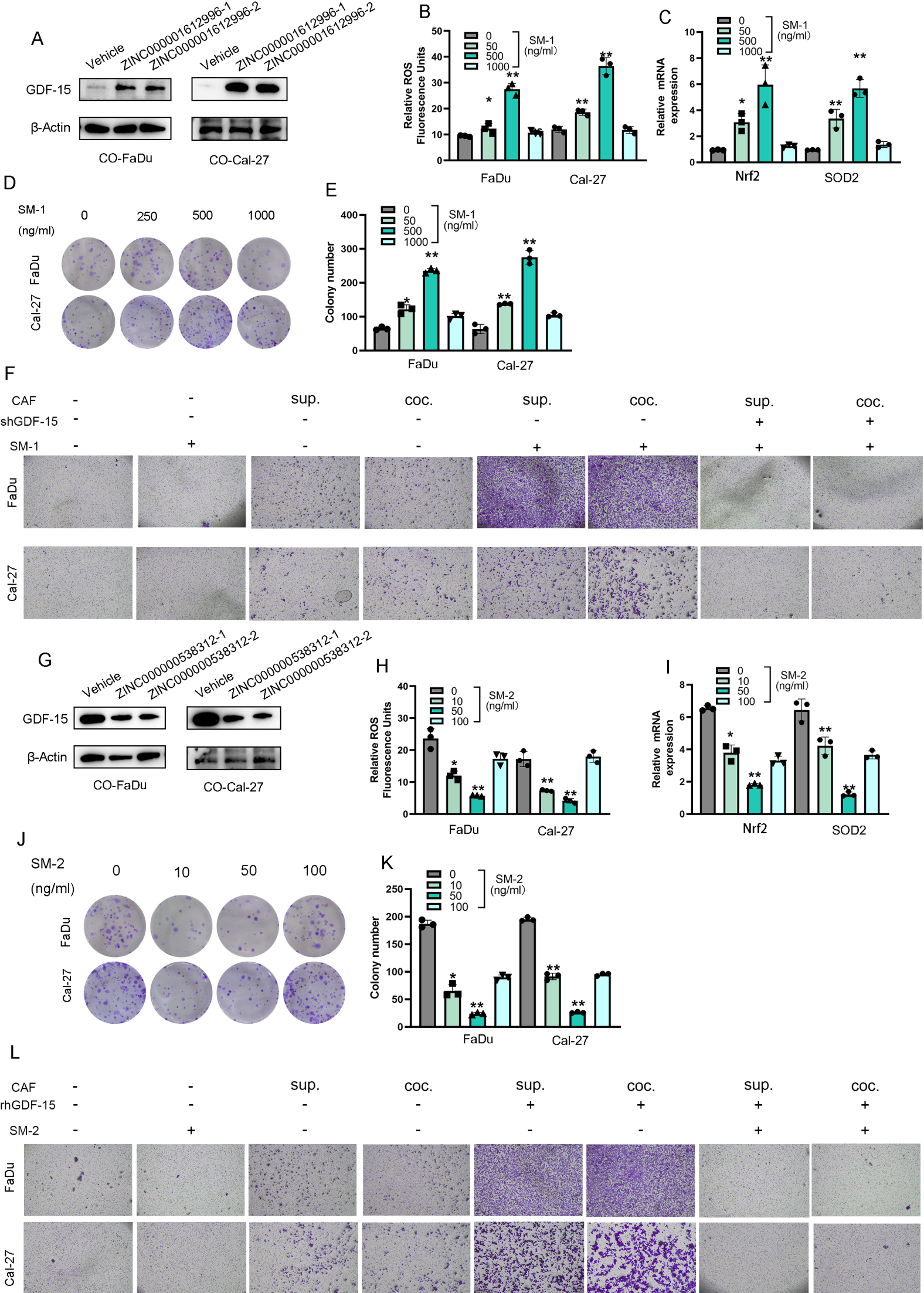

Supplement: Supplementary 1 — Materials and Methods Figs. S1 to S11 Table S1 [file research.0901.f1.zip › Supplementary Figure 11.pdf]

**A**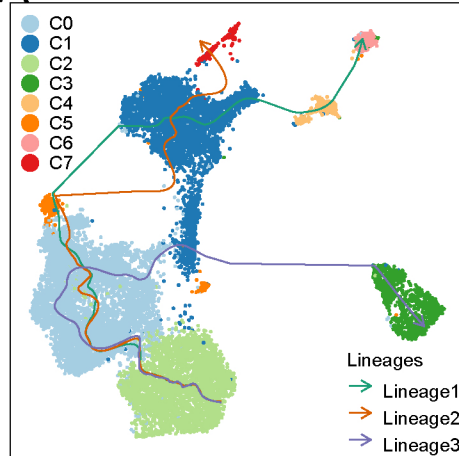**B**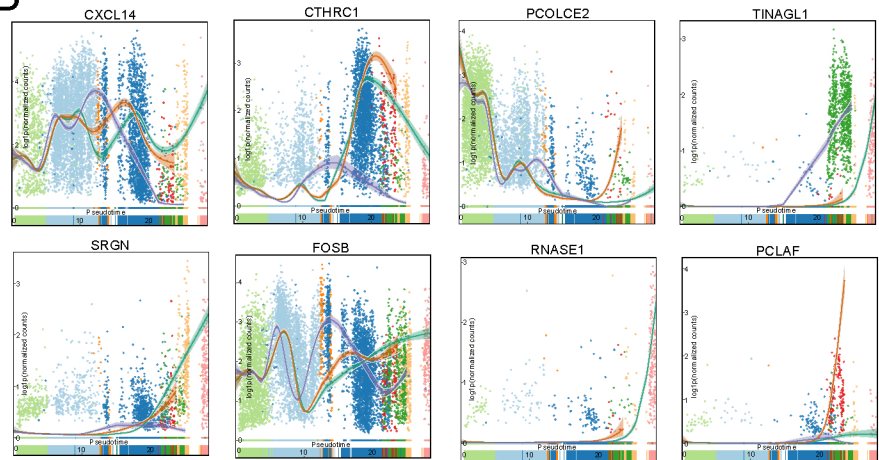**C**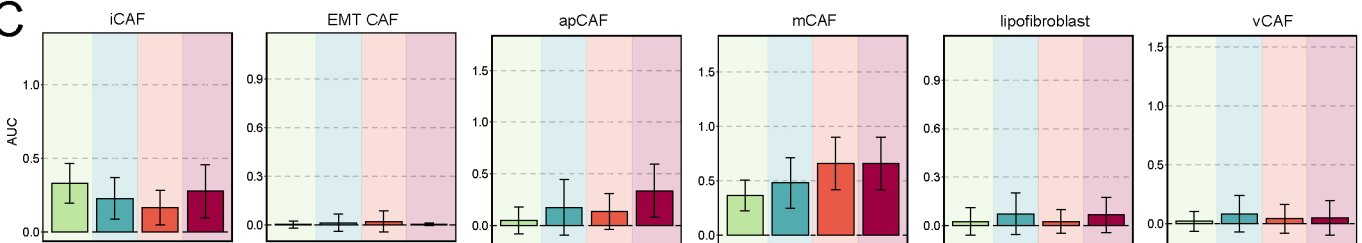**D**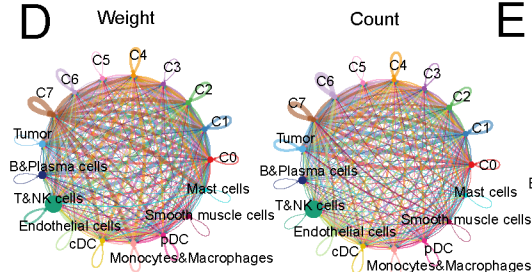**E**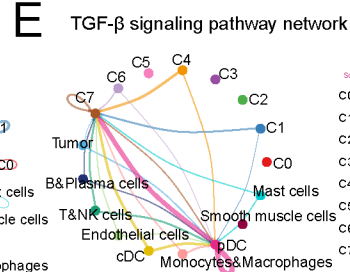**F**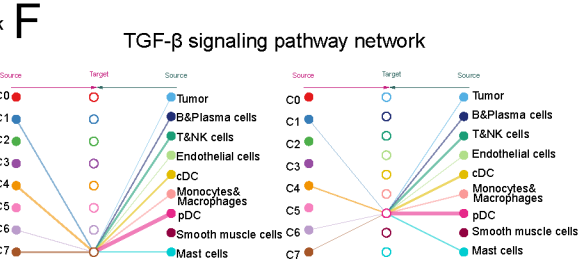**G**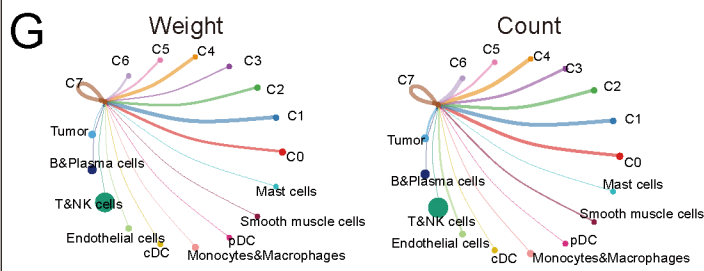**H**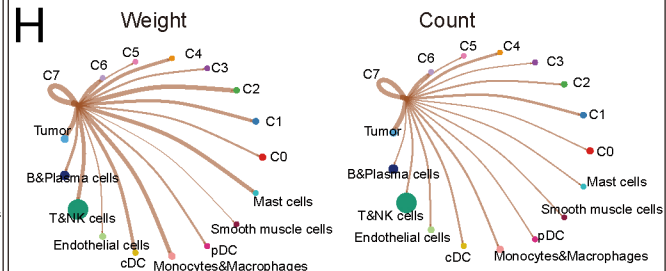**I**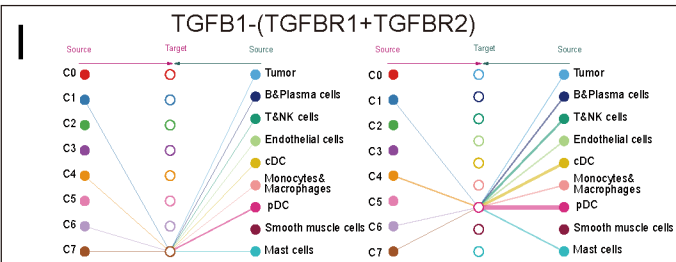**J**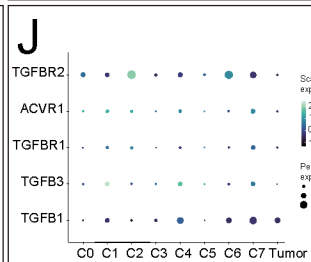**K**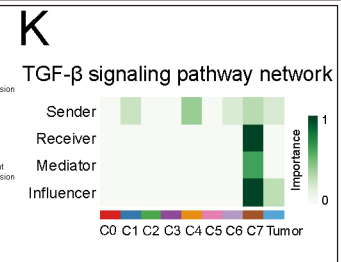

Supplement: Supplementary 1 — Materials and Methods Figs. S1 to S11 Table S1 [file research.0901.f1.zip › Supplementary Figure 2.pdf]

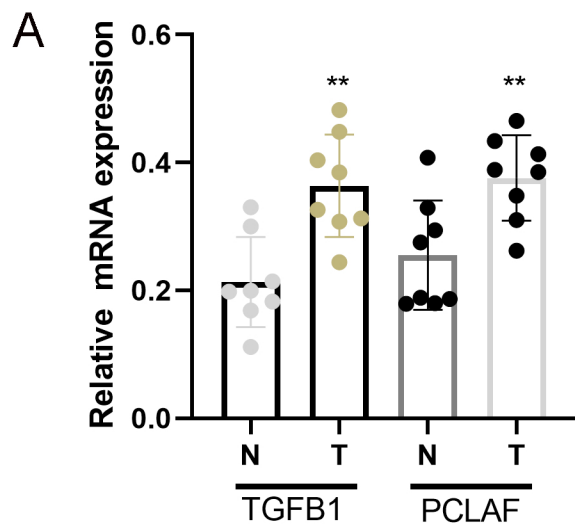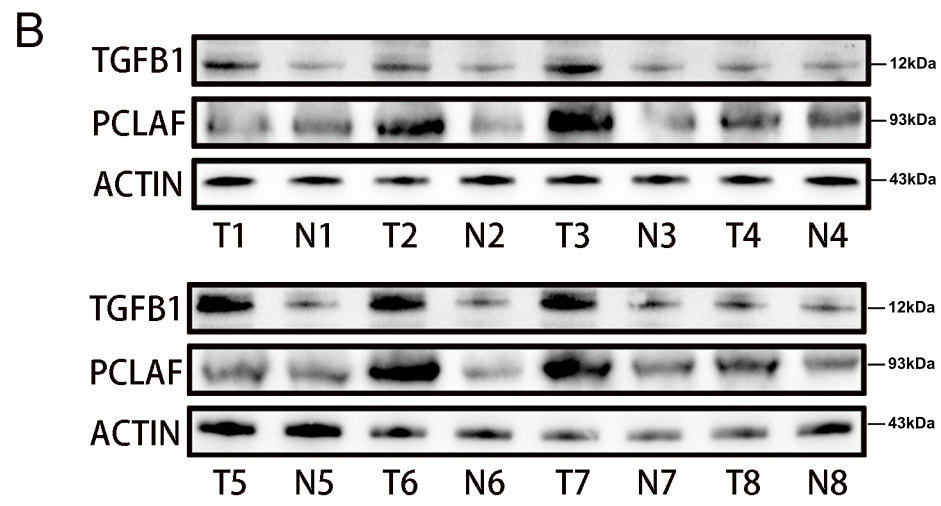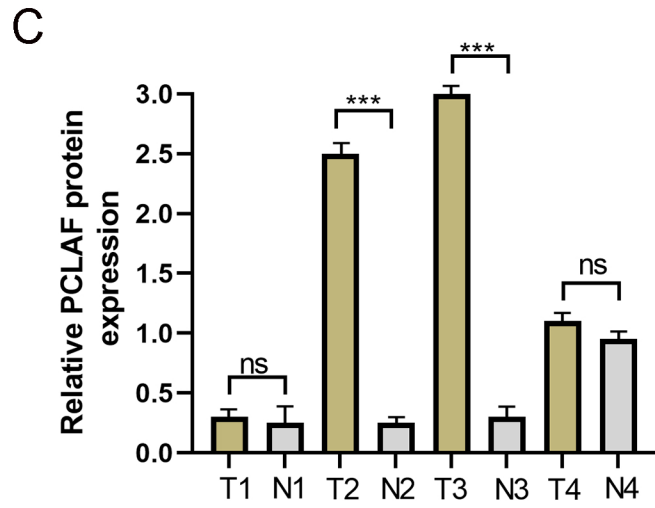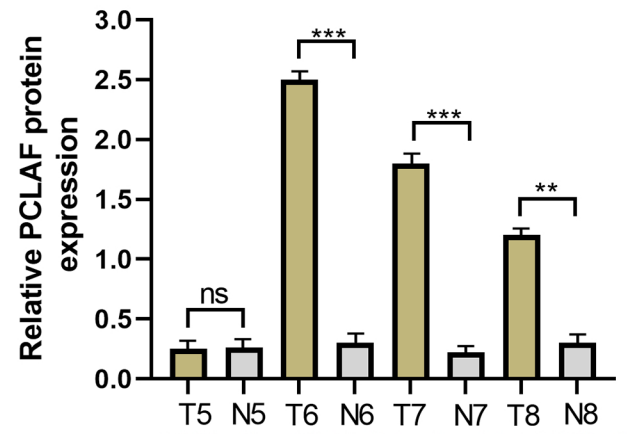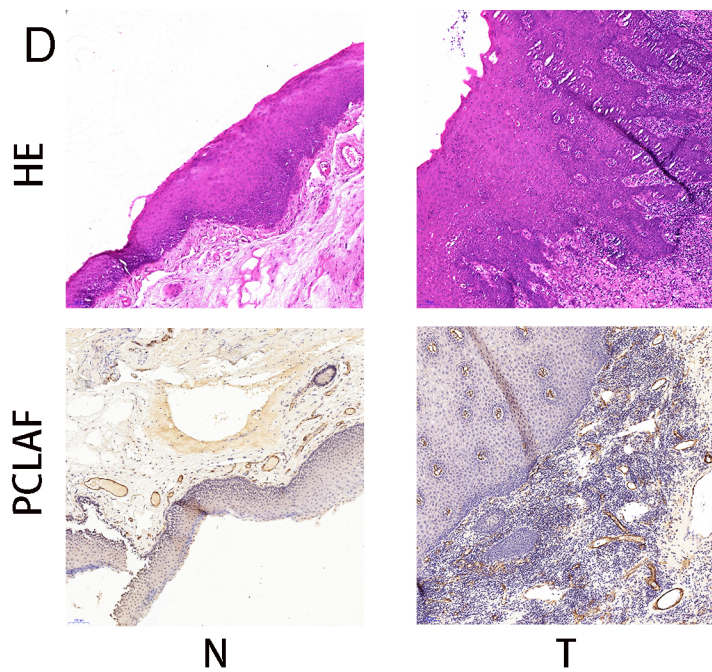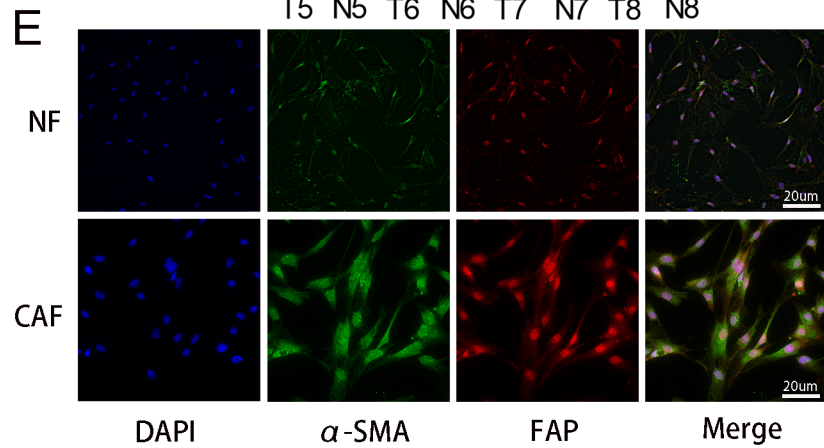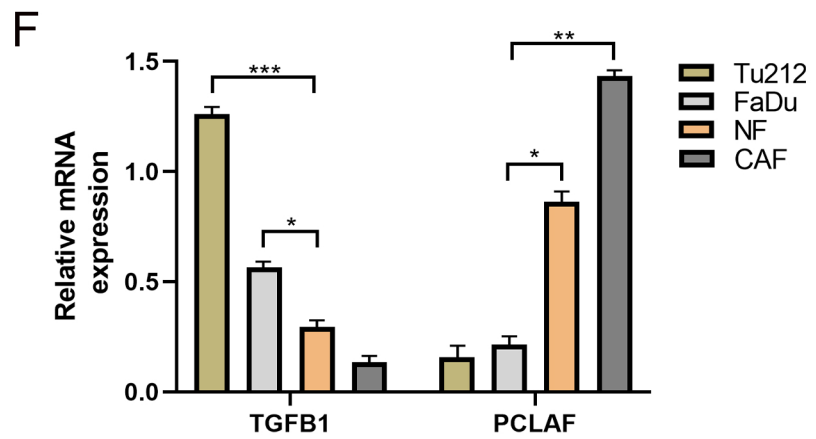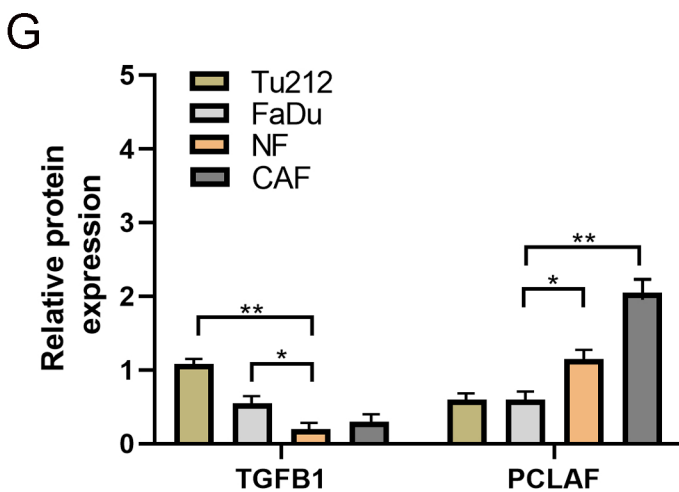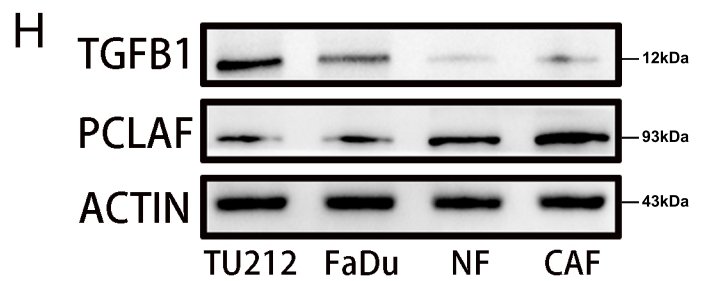

Supplement: Supplementary 1 — Materials and Methods Figs. S1 to S11 Table S1 [file research.0901.f1.zip › Supplementary Figure 3.pdf]

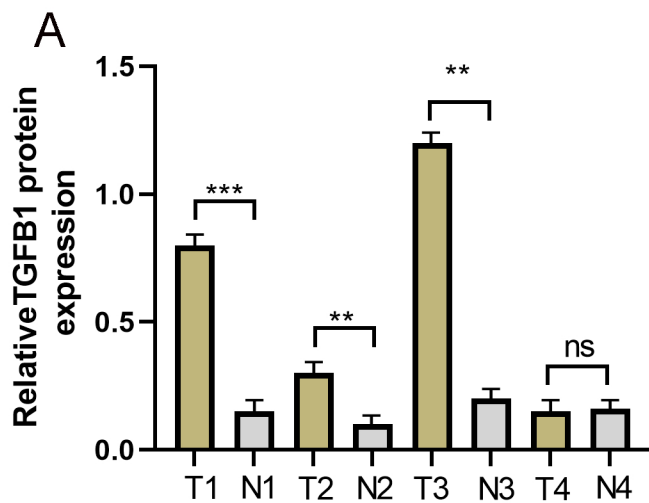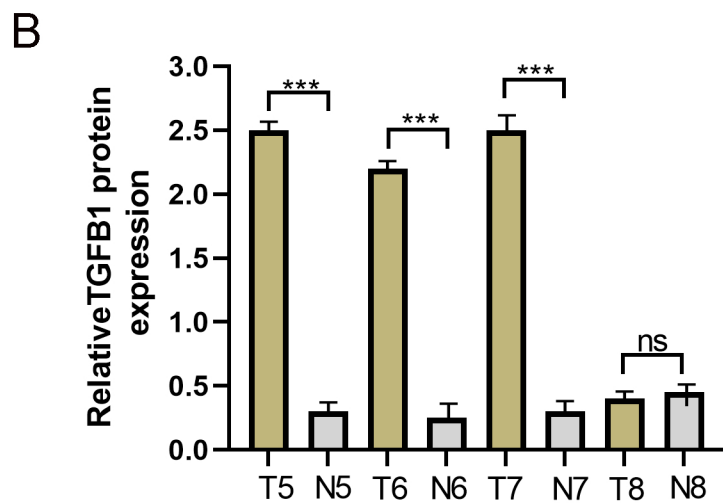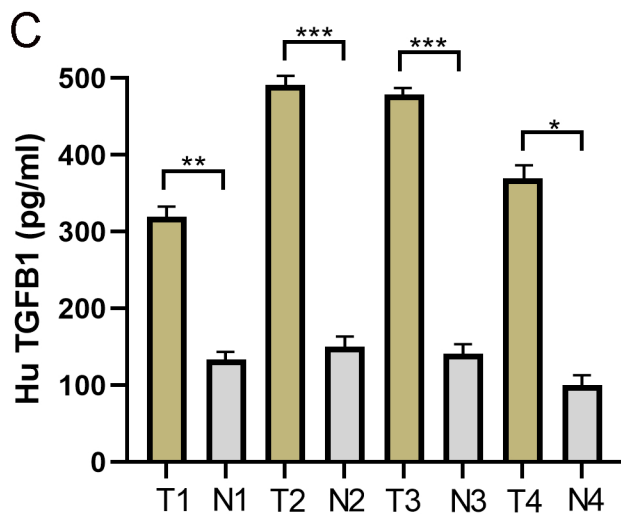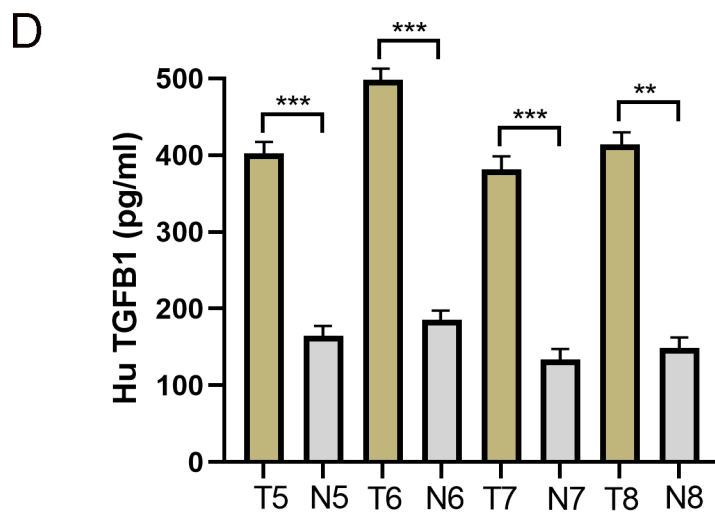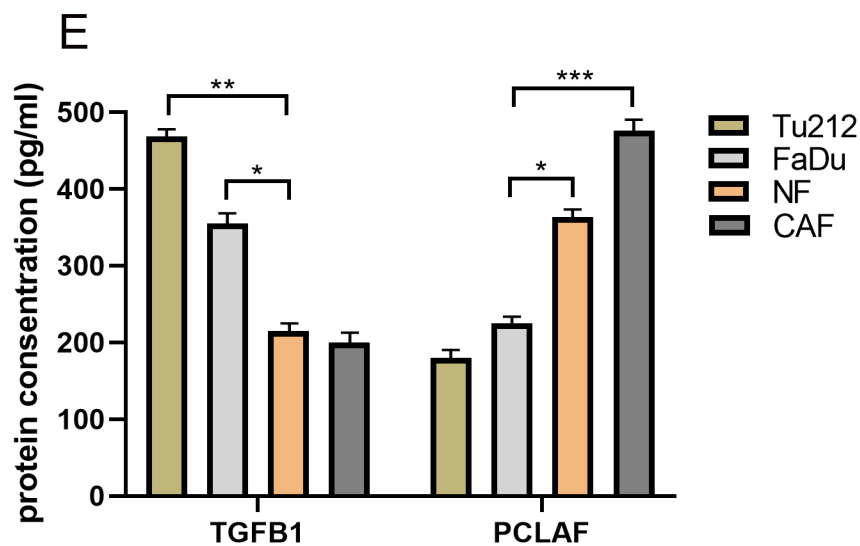

Supplement: Supplementary 1 — Materials and Methods Figs. S1 to S11 Table S1 [file research.0901.f1.zip › Supplementary Figure 4.pdf]

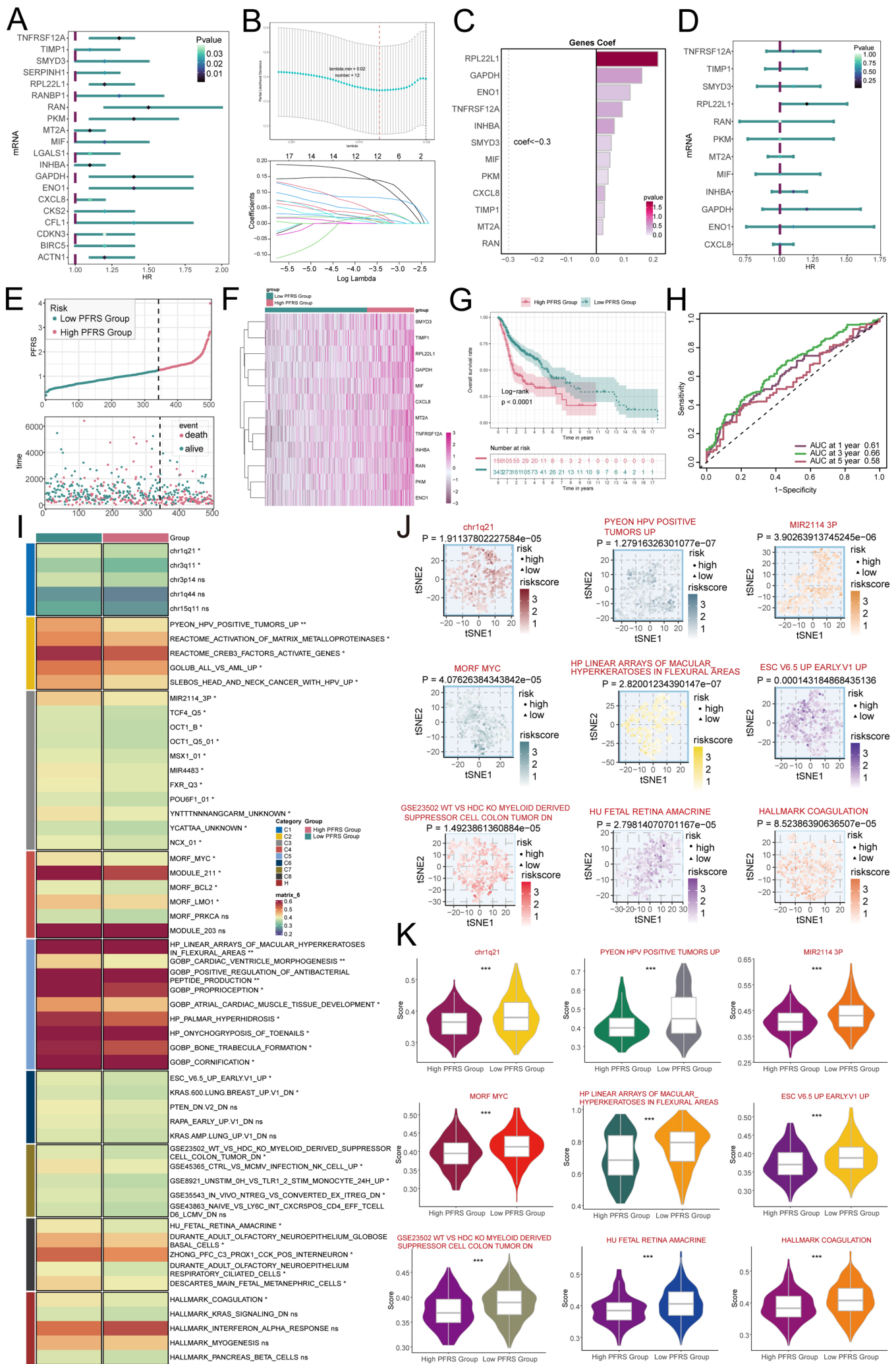

Supplement: Supplementary 1 — Materials and Methods Figs. S1 to S11 Table S1 [file research.0901.f1.zip › Supplementary Figure 6.pdf]

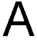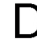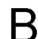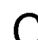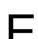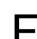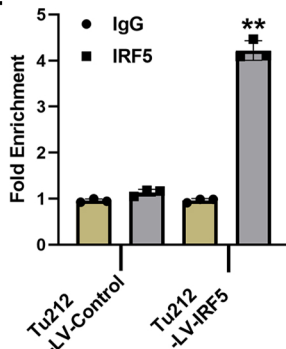

Supplement: Supplementary 1 — Materials and Methods Figs. S1 to S11 Table S1 [file research.0901.f1.zip › Supplementary Figure 7.pdf]

A

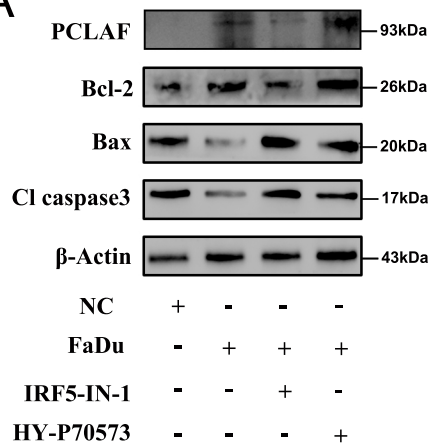

B

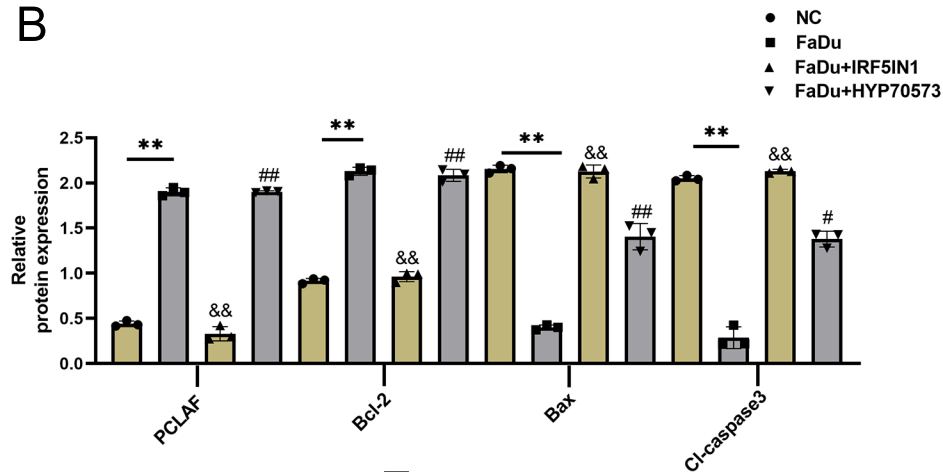

C

Human HNSCC

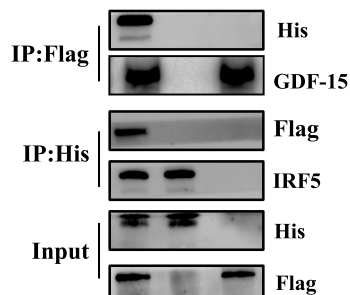

| Flag-GDF-15 | His-IRF5 |
|-------------|----------|
| +           | +        |
| -           | +        |
| +           | -        |

D

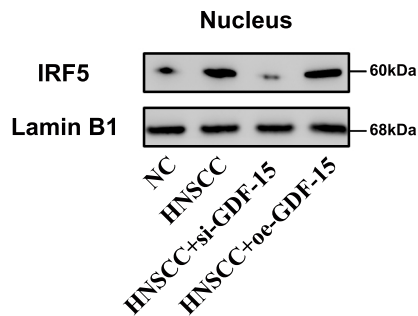

E

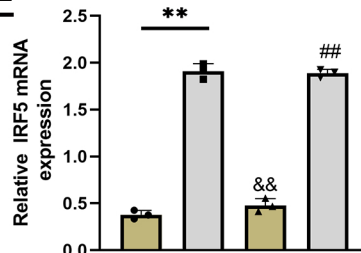

F

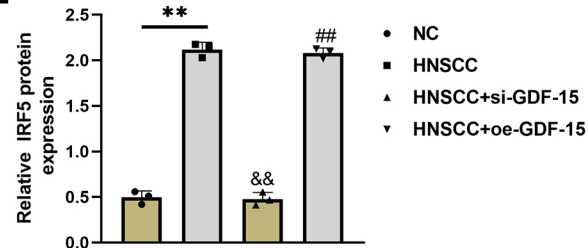

Supplement: Supplementary 1 — Materials and Methods Figs. S1 to S11 Table S1 [file research.0901.f1.zip › Supplementary Figure 8.pdf]

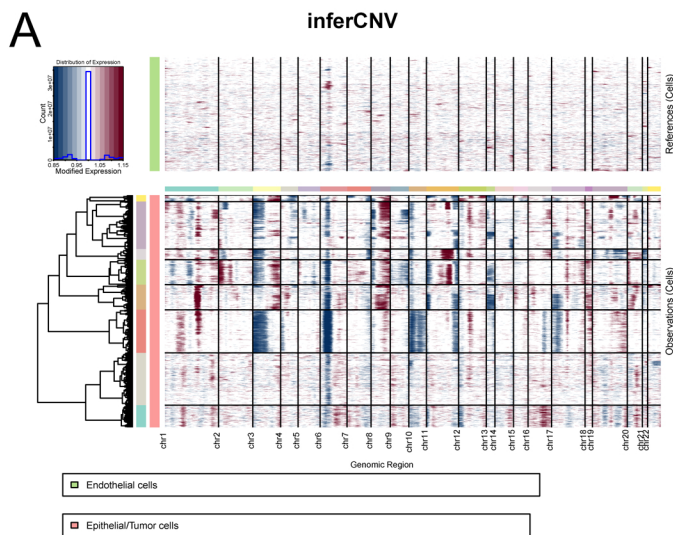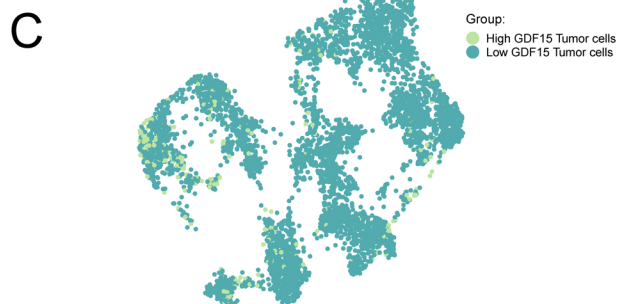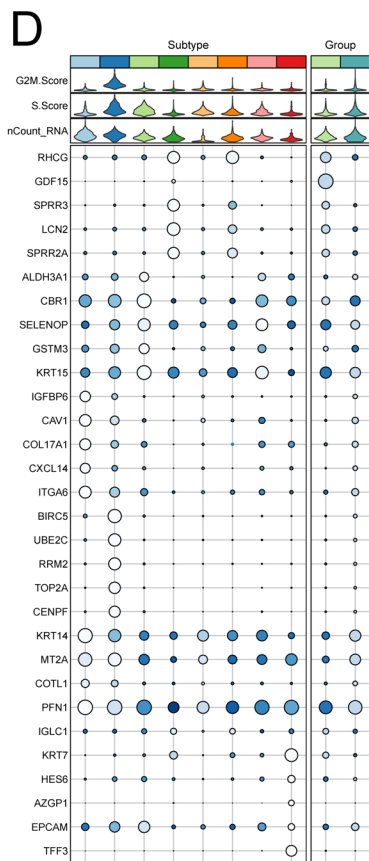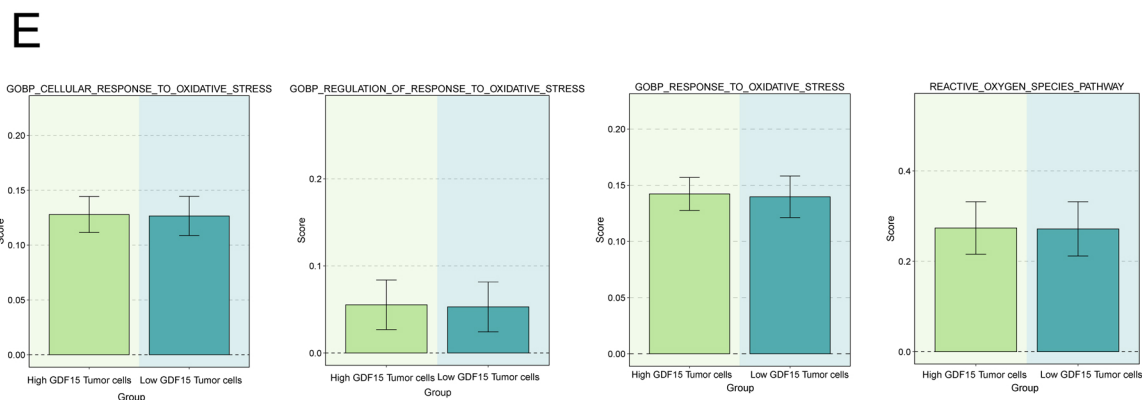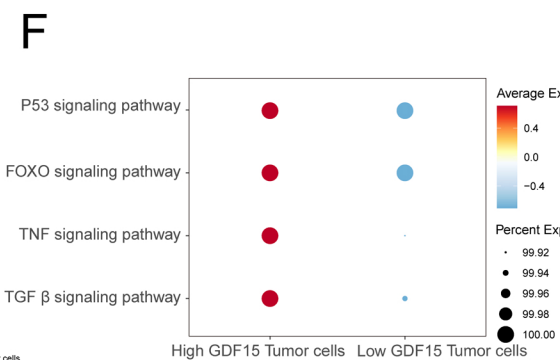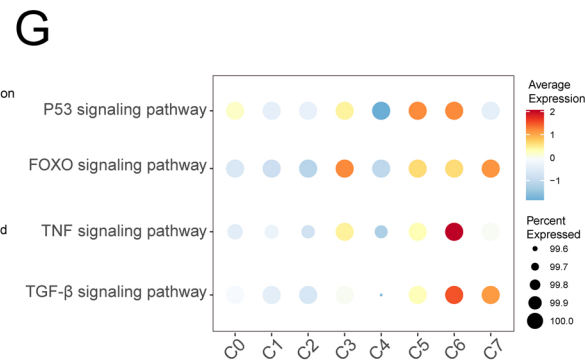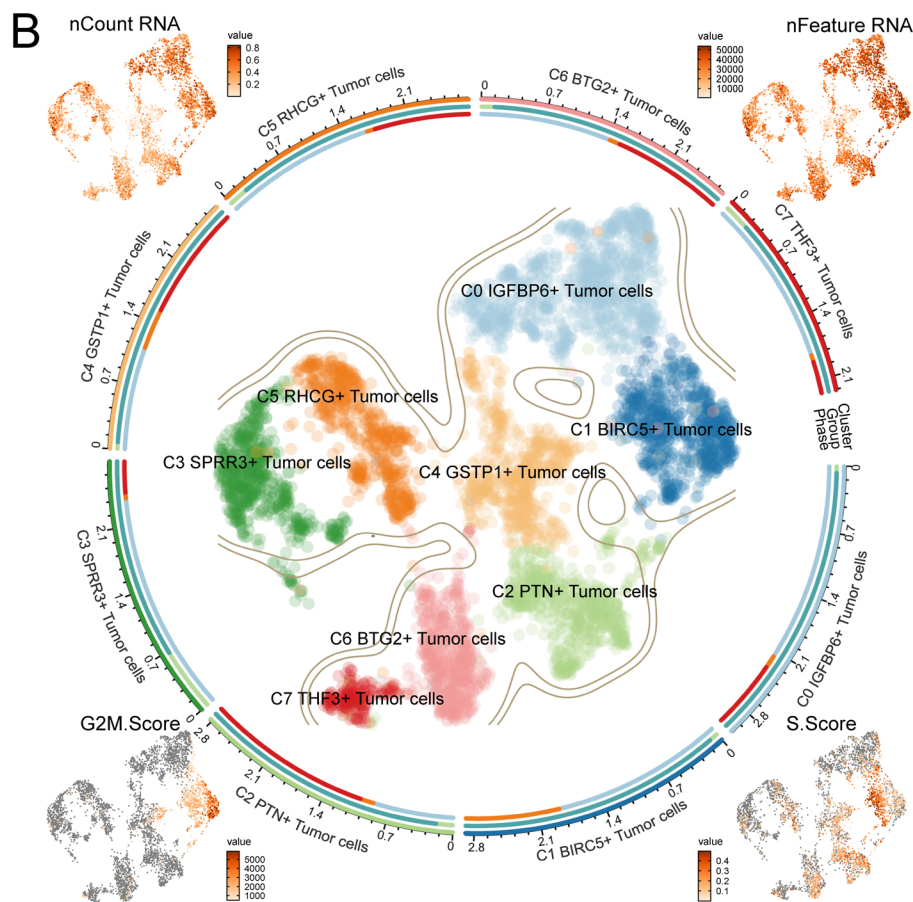

Supplement: Supplementary 1 — Materials and Methods Figs. S1 to S11 Table S1 [file research.0901.f1.zip › Supplementary Figure 9.pdf]
